# Shape-Matching and Halogen Bonding in Chiral Pyrazine-Allene Hosts: Confining an Unstable Guest Conformation

**DOI:** 10.1021/acs.orglett.5c02075

**Published:** 2025-06-24

**Authors:** Víctor Rubio-Pisabarro, Jonathan Álvarez-García, María Magdalena Cid

**Affiliations:** Departamento de Química Orgánica, Edificio Ciencias Experimentais, 16784 Universidade de Vigo, E-36310 Vigo, Spain

## Abstract

A novel chiral host with axially chiral allenes and pyrazine units was synthesized in high yield via a three-step sequence. In the solid-state, it adopts a twist-boat conformation, forming discrete double chains in an extended network. Host–guest studies with halogen bond donors, analyzed by NMR and X-ray diffraction, revealed size-selective inclusion within the macrocyclic cavity. Remarkably, a syn-gauche conformer of diiodoperfluorobutane was crystallized inside the host, highlighting the role of guest structure, host flexibility, and crystallization conditions.

## Graphical abstract

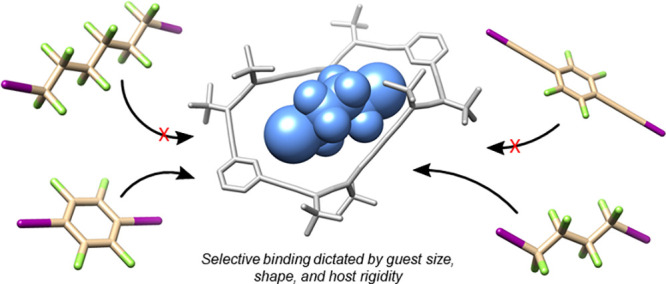

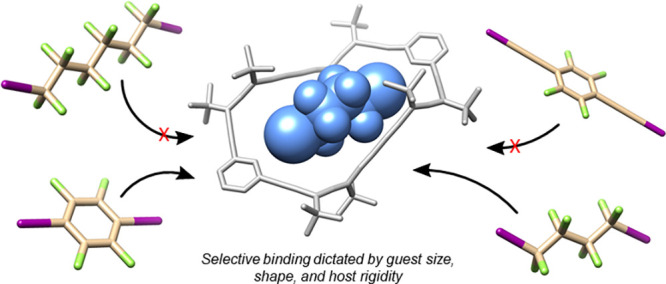

Halogen bonding has gained increasing importance across various fields of chemistry, thanks to its unique properties—such as directionality, strength, and tunability—that make it an ideal tool for designing sophisticated molecular systems.^1,2^ This interaction has shown exceptional utility in constructing supramolecular architectures, including interlocked structures,^3,4^ supramolecular polymers,^5^ and host–guest complexes.^6,7^ Notably, chiral host–guest complexes hold immense promise for diverse applications.^8^ When the host molecules are chiral, they can facilitate the development of innovative chiral switches and sensors,^9^ chiral chromatographic layers for separating racemic halogenated compounds,^10^ and tools for asymmetric catalysis.^11^

Due to the strong electron-withdrawing effect of fluorine atoms, per- or polyfluorinated compounds (PFCs) effectively form halogen bonding interactions. Their high chemical stability makes them resistant to biological degradation, classifying them as environmentally persistent compounds prone to bioaccumulation.^12^ Concurrently, PFCs are linked to a range of toxic effects, including hepatotoxicity, reproductive toxicity, and carcinogenicity.^13–15^ Their widespread presence has raised global concerns, underscoring the importance of their selective capture and detection.^16,17^

Pyridines, pyrimidines, and pyrazines are among the most commonly used six-membered nitrogen heterocycles as acceptors in the formation of halogen-bonded adducts.^18–20^ Building on our previous work, which demonstrated the formation of an inclusion complex between the axially chiral host [14_2_]-pyridoallenophane **1** (Scheme 1) and 1,4-diiodooctafluorobutane,^21^ we identified a compelling opportunity to enhance the system’s functionality. By replacing the aromatic pyridine ring with a pyrazine ring (as in **2**, Scheme 1), we can harness the two nitrogen atoms to introduce additional coordination sites in a shape-persistent macrocycle, significantly boosting its capabilities.

**Scheme 1:**
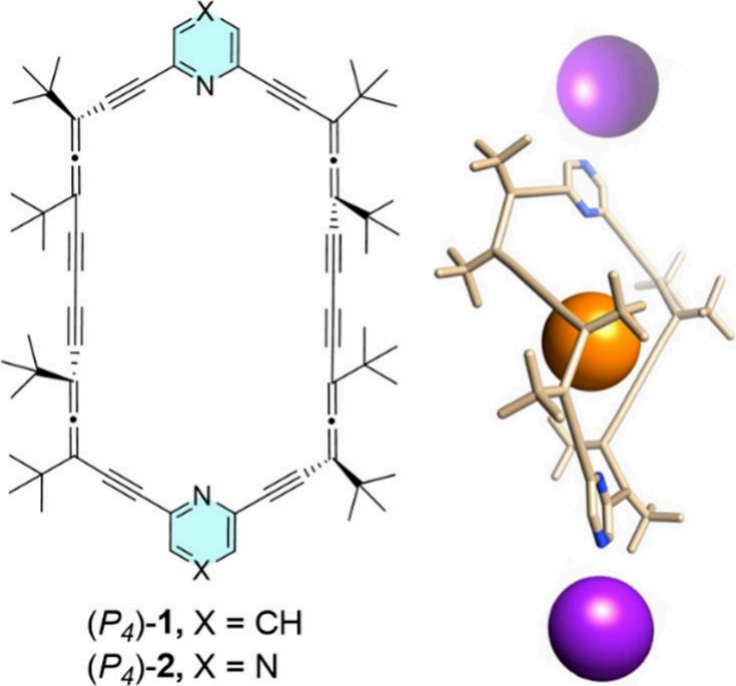
Structure of **1** and **2** ^a^

a Recognition zones of allenophane **2** represented by orange/purple spheres.

The internal nitrogen atoms enable coordination with guests of suitable size that match the well-defined host cavity, leveraging a chelation effect, while the external nitrogen atoms can interact with larger guests that are too big for the cavity. Therefore, the presence of these two distinct types of nitrogen atoms, bestows the macrocycle with the ability to carry out selective complexation based on the structural features of the molecule of interest.

Herein, we present the preparation of novel chiral shape-persistent host based on axially chiral allenic units and pyrazine moieties as recognition sites. This host is employed for the complexation of various halogen-bond donors, with a focus on studying their selectivity and interaction patterns. Characterization techniques, including 2D NMR spectroscopy HMBC ^1^H–^15^N and X-ray crystallography, provide detailed insights into the host–guest interactions yielding a chiral supramolecular assembly via halogen bonding networks. Interestingly, we crystallized a highly unstable conformer of diiodooctafluorobutane in a host–guest complex driven by halogen-bonding interactions. Using the methodology previously established in our research group,^21,22^ host **2** was prepared in a three-step reaction sequence with high overall yields.

## Synthesis and Characterization

The synthesis began with a Sonogashira cross-coupling reaction between 2,6-diiodopyrazine and enantiopure allene (*P*)- or (*M*)-**DEA**,^23,24^ affording the intermediate (*P*_2_)- or (*M*_2_)-**3** with a 81% yield (Scheme 2). Subsequent removal of the acetonide protecting group and an intermolecular ring closure under Breslow conditions yielded the target compound, (*P*_4_)- or (*M*_4_)-**2**, with a 53% global yield. These novel allenophanes showed notable chiroptical responses, with a dissymmetry factor of *g* = 0.005 (Scheme 2, bottom).

**Scheme 2:**
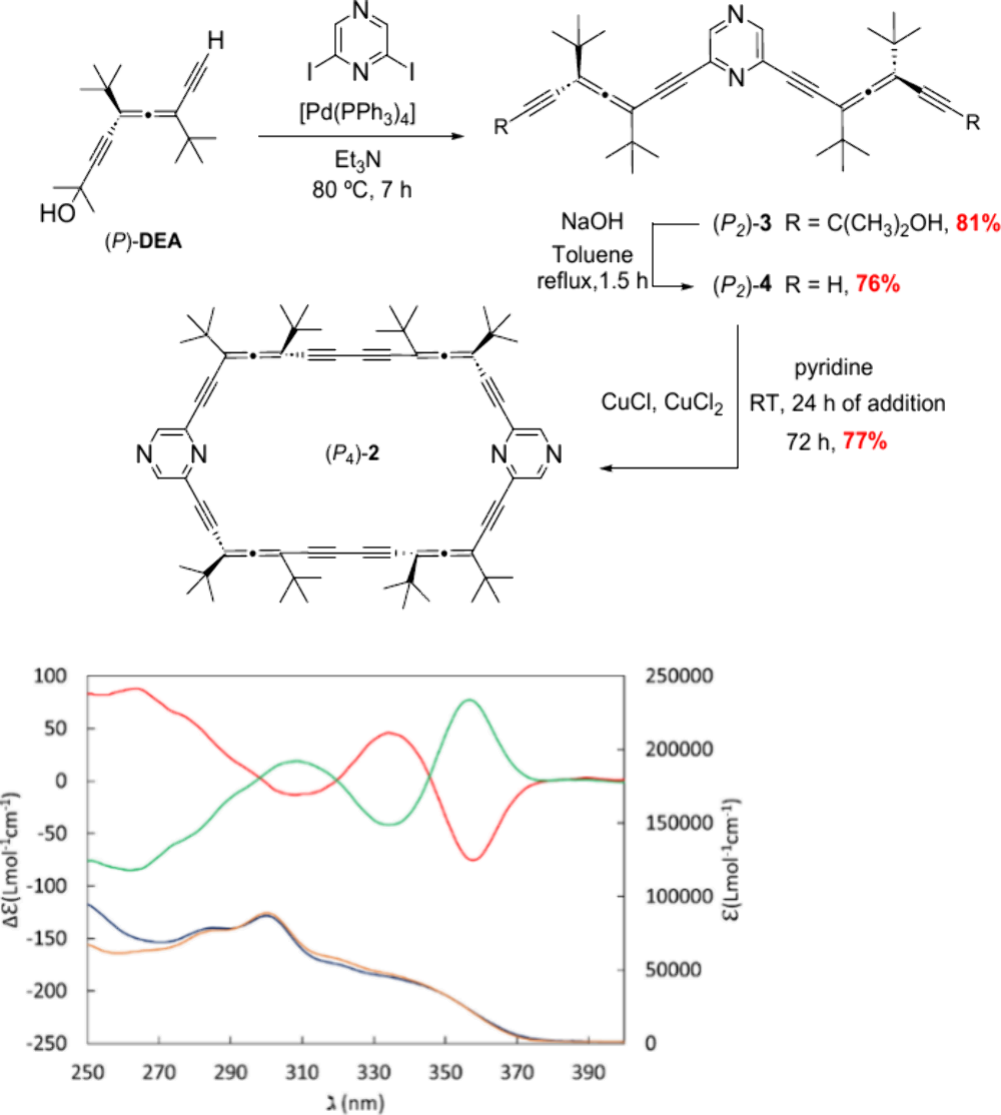
Synthesis of (*P*_4_)- and (*M*_4_)-2; UV–vis (Blue and Orange Lines)–ECD (Red and Green Lines) Spectra of (*P*_4_)-2 and (*M*_4_)-2, Respectively (6.24 × 10^–6^ M, CHCl_3_)

A DFT study at the CAM-B3LYP/6-31g+(d,p) level revealed that allenophane **2** exists as an equilibrium of four conformers—boat, chair, twist and twisted-boat—each displaying unique cavity dimensions and pyrazine-nitrogen orientations, with internal N–N distances ranging from 10.17 to 12.75 Å (Figure S19b in Supporting Information). This level of theory was previously validated as sufficient to explore the conformational space of allenophanes and to reproduce their circular dichroism spectra with excellent accuracy and computational efficiency.^25^ Harmonic frequency calculations confirmed that all conformers are true minima, with no imaginary frequencies. Although [14_2_]-allenophanes **1** and **2** were expected to have similar conformational spaces, compound **1** predominantly adopts chair and twist conformers,^25^ while the presence of a boat conformer in **2** cannot be excluded due to the absence of clear vibronic coupling features.

Notably, X-ray diffraction of crystals of (*P*_4_)-**2**, obtained by slow cooling of a hot ethanol solution, revealed that allenophane **2** adopts a well-defined twisted boat conformation (Figure 1a, CCDC 2440451), previously described computationally. This conformation plays a key role in promoting one-dimensional chain formation along the crystallographic *b*-axis, accompanied by lateral-displacement of the pyrazine rings along the *a*-axis. The packing is characterized by intermolecular distances of 3.20–3.43 Å and dihedral angles of 13.5°–17.4°, in which allenophane **2** acts as a molecular clip, guiding the directional assembly into double chains (Figure 1b and Figure S10b in Supporting Information). This contrasts with the crystal structure previously reported for compound (*P*_4_)-**1**, where the allenophane adopts a *D*_2_-symmetric twist conformation and stacks in a columnar fashion, while (*P*_4_)-**2** assembles into compact discrete chains.^21^

**Figure 1:**
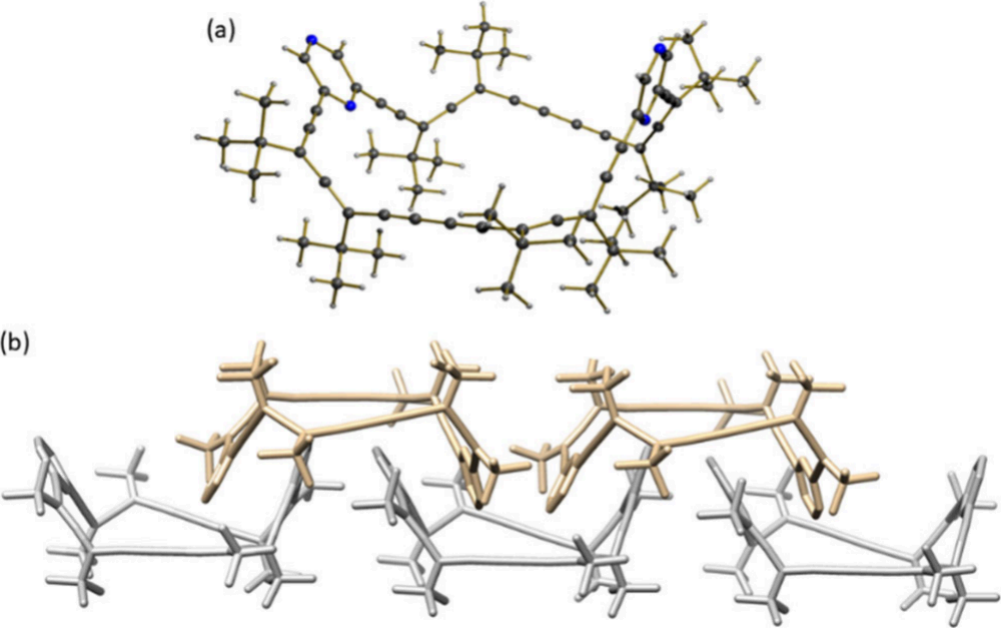
X-ray crystal structure of (*P*_4_)-**2** (a) and self-assembly of the allenophane into chains (b) obtained by X-ray diffraction.

To explore the electron density distribution in allenophane **2**, 2,6-di­(prop-1-yn-1-yl)­pyrazine was used as a model. The electrostatic potential map (B3LYP-D3/aug-cc-pVTZ/aug-cc-pVTZ-PP) highlights electron-rich regions at the nitrogen atoms, with nitrogen 1 showing greater density due to the presence of alkynyl groups in *ortho* position (Figure 2a). In parallel, to assign the two nitrogen atoms in (*P*_4_)-**2** by NMR, a ^1^H–^15^N HMBC experiment was performed.^21^ This technique enhances signal intensity compared to direct ^15^N detection, overcoming limitations related to the isotope’s low natural abundance and gyromagnetic ratio. The resulting spectrum showed two well-defined signals, a singlet at 336 ppm and a doublet (*J* = 11.2 Hz) at 334 ppm. Based on the observed splitting^26^ and GIAO/DFT calculations, these resonances were assigned to the blue and red nitrogen atoms, respectively (Figure 2b).

**Figure 2:**
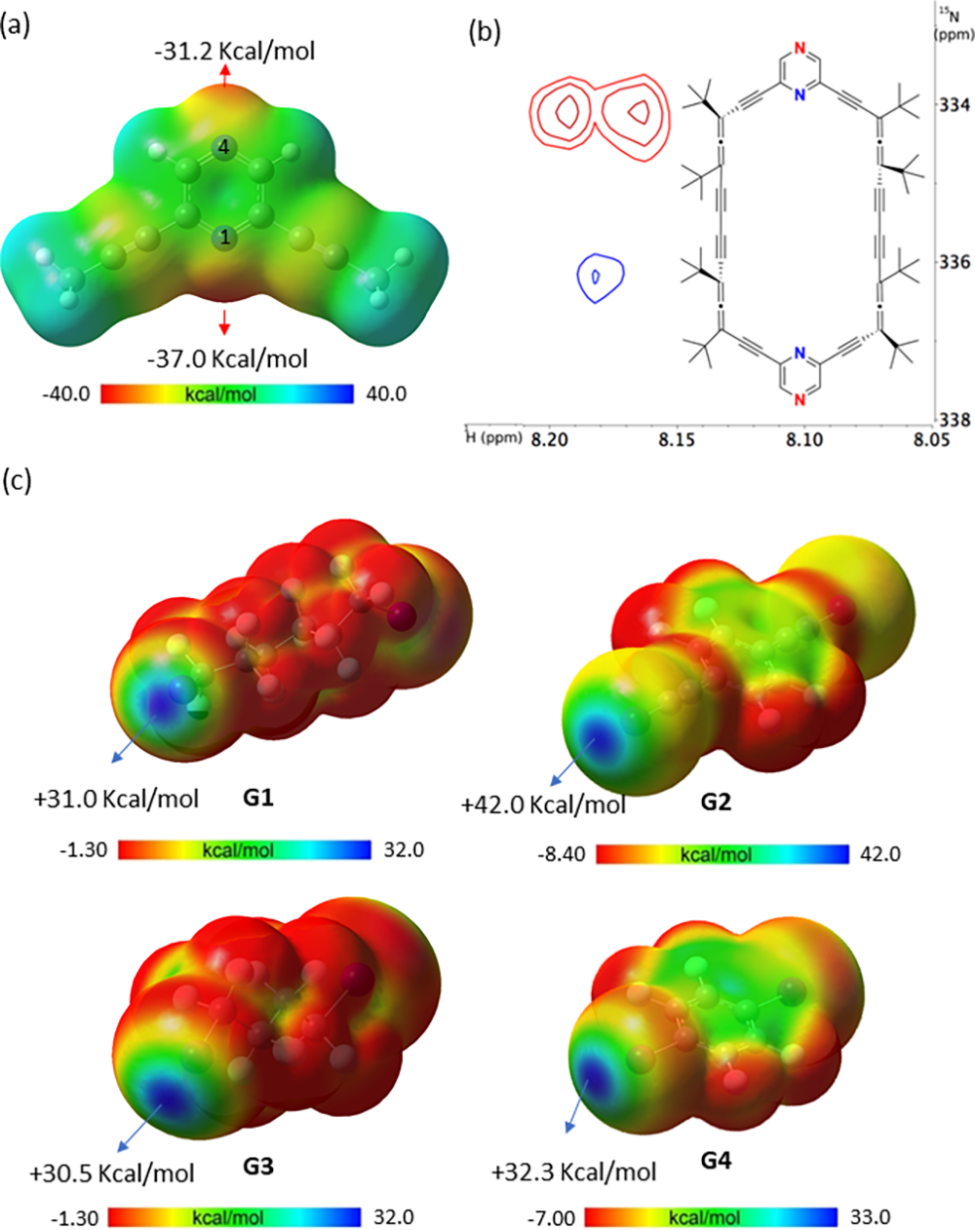
(a) Electrostatic potential (ESP, isovalue = 0.001 au) map of model pyrazine (B3LYP-D3/aug-cc-pVTZ/aug-cc-pVTZ-PP) showing the extrema value V_s,min_ of the electronegative region. (b) ^1^H–^15^N HMBC NMR spectrum of (*P*_4_)**-2** in C_6_D_6_ with signal assignments. (c) Computed ESP (isovalue = 0.001 au) of **G1**, **G2**, **G3** and **G4** (B3LYP-D3/aug-cc-pVTZ/aug-cc-pVTZ-PP), along with the extrema values V_s,max_ of the electropositive area (σ-holes).

## Host–Guest Complexes: Solution

Next, to investigate the halogen bond-accepting ability of (*P*_4_)-**2**, a series of structurally diverse PFCs were selected as guests: 1,6-diiodododecafluorohexane (**G1**), 1,2,4,5-tetrafluoro-3,6-bis­(iodoethynyl)­fluorobenzene (**G2**), 1,4-diiodooctafluorobutane (**G3**), and 1,4-diiodotetrafluorobenzene (**G4**). To better understand the potential σ-hole interactions, we performed electrostatic potential (ESP) calculations on **G1**–**G4** (see Supporting Information for full computational details). As shown in Figure 2c, a strongly electron deficient region is generated for each iodine atom in the prolongation of the C–I bond, with maximum surface potential (V_s,max_) ranging from 30.5 kcal/mol in **G3** to 42.0 kcal/mol in **G2**. Note that the V_s,max_ value for the σ-hole in **G4** is comparable to that reported using a similar computational approach,^27^ whereas for **G2**, it is slightly lower than the value reported in the literature.^28^ These findings suggest that halogen bonding interactions between (*P*_4_)-**2** and the selected guests are likely, given the pronounced σ-hole character observed on the iodine atoms of **G1**–**G4**.

On the other hand, preliminary computational models (see Figures S20 and S21) suggest that both **G1** and **G2** can interact with the internal and external nitrogen atoms of the pyrazine rings in (*P*_4_)-**2** with little selectivity, as chelation within the macrocyclic cavity is not feasible due to the size of the potential guests. In contrast, **G3** and **G4**, which are better sized to fit within the cavity, form well-defined 1:1 inclusion complexes involving the inner nitrogen atoms, supported by near-ideal halogen bond geometries (Figures S22a and S23).

Thus, titration experiments were carried out in deuterated benzene (C_6_D_6_) using ^1^H–^15^N HMBC NMR as the main monitoring technique, with nitrogen atoms serving as sensitive reporters of established halogen interactions. **G1** and **G2** led to similar deshielding effects of both nitrogens indicating nonselective interactions (Figures S26 and S27). Pronounced changes were observed with **G3** and **G4** (Figure 3). Upon addition of **G3,** selective shielding of the inner nitrogen was initially observed (at 20 eq, ΔδN_int_^15^ = −1.3 ppm, ΔδN_ext_^15^ = −0.2 ppm), consistent with complexation occurring within the macrocyclic cavity. At higher guest concentrations, both nitrogen atoms became shielded, indicating additional interactions involving the outer nitrogens. Global 1:1 fitting using BindFit software^29,30^ yielded a binding constant of 1.03 ± 0.04 M^–1^. Titration with **G4** resulted in stronger and more selective binding: only the inner nitrogen was significantly shielded (336 to 330 ppm), while the outer remained essentially unchanged. The association constant for **G4** was the highest among all guests tested, 3.94 ± 0.06 M^–1^, reflecting both a chelation effect and the enhanced halogen bond donor strength of iodine on sp^2^-hybridized carbons,^31^ comparable to other reported halogen bonds.^32^

**Figure 3:**
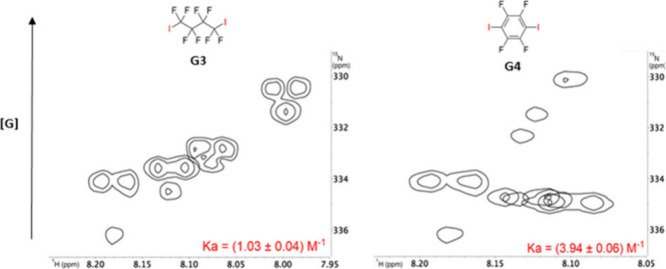
^1^H–^15^N HMBC NMR titration experiments of (*P*_4_)**-2** with **G3** and **G4**, along with the calculated association constants.

^1^H NMR titrations (see Supporting Information) showed a consistent trend across all guests: shielding of the aromatic proton (ΔδH ≈ −0.20 ppm), while the *tert*-butyl signals moved downfield (ΔδH ≈ + 0.05 ppm). An exception was observed for **G4**, where both *tert*-butyl signals collapsed into a single resonance. This distinct behavior arises from the vertical encapsulation of the guest’s aromatic ring, which induces anisotropic ring current effects that differentially influence the *tert*-butyl groups, leading to signal coalescence.

These results highlight the critical role of size complementarity in achieving selective halogen-bonded host–guest complexes. Larger or more flexible guests, like **G1** and **G2**, interact nonselectively with both nitrogen sites. In contrast, guests that better match to the cavity size, such as **G3** and **G4**, show a clear preference for binding through the internal nitrogen atoms, leading to stronger and more well-defined complexes.

## Host–Guest Complexes: Solid State

To explore host–guest interactions beyond solution, single-crystal X-ray diffraction was employed. Slow diffusion of acetonitrile into C_6_D_6_ solutions containing the host–guest mixtures yielded suitable crystals for **G3** and **G4** complexes, enabling detailed structural analysis. Both chiral **G3**@(*P*_4_)-**2** and **G4**@(*P*_4_)-**2** complexes feature halogenated guests accommodated within the macrocyclic cavity, stabilized by directional halogen bonds (Figure 4). For **G3**@(*P*_4_)-**2**, the average N···I distance is 2.95 Å with a C–I···N angle of 169.2°, indicating strong yet slightly distorted halogen bonding. Remarkably, the diiodoperfluorobutane guest adopts a syn-gauche conformation with a dihedral angle of 36° that would typically be inaccessible due to steric and electronic repulsions (Figure 4a, CCDC 2440456). This unusual conformation causes a structural adjustment in the macrocycle (N···N = 13.29 Å).

**Figure 4:**
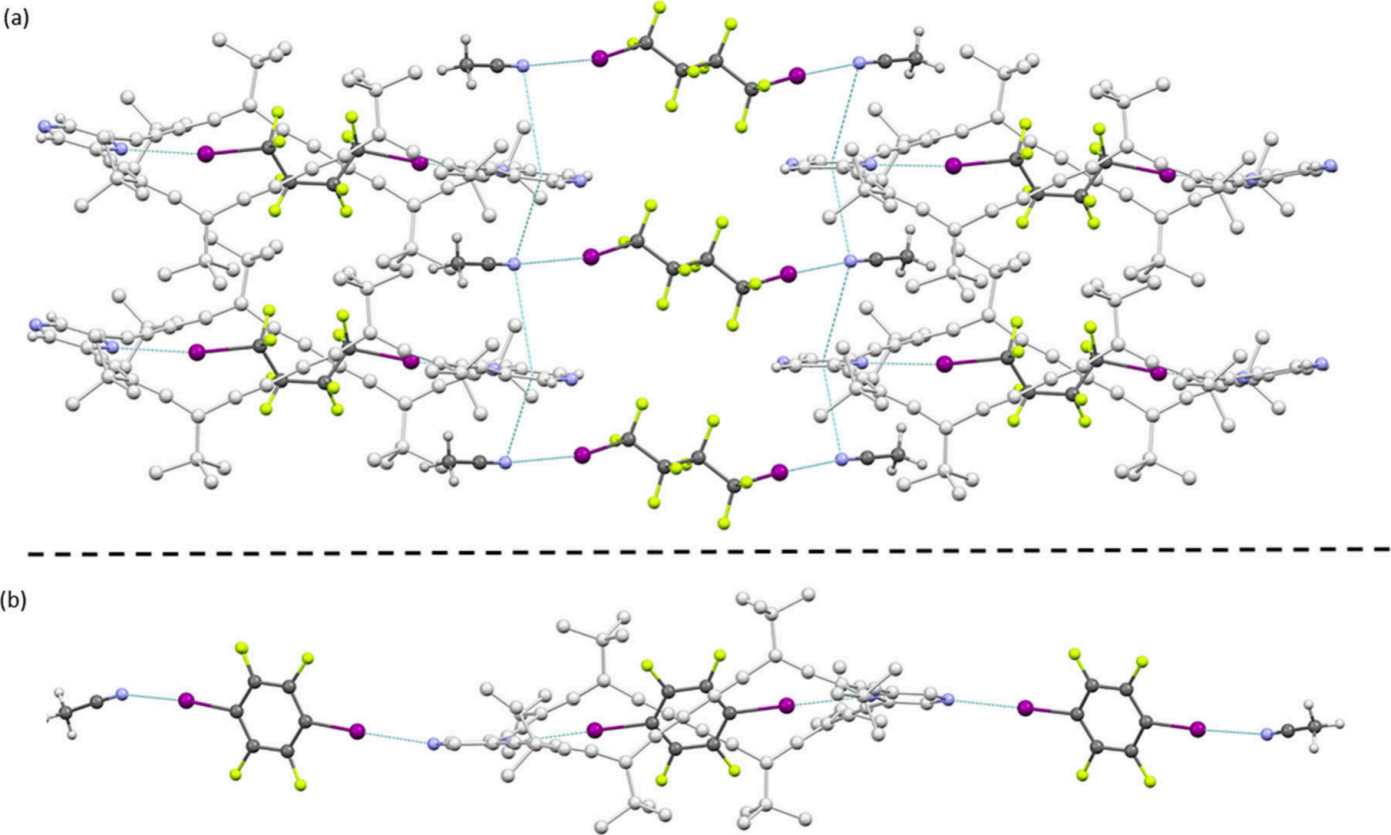
Crystal structure of the **G3**@(*P*_4_)-2 (a) and **G4**@(*P*_4_)-2 (b) complex.

**G4**@(*P*_4_)-**2** exhibits nearly ideal halogen bonding (N···I = 3.07 Å, C–I···N = 176.3°), closely aligning with computational models. These differences reflect how guest rigidity and symmetry shape complex geometry, and they correlate well with solution-phase data: the more rigid, **G4**, forms the strongest and most selective complex, while the conformational flexibility of **G3** induces slightly weaker binding and structural distortion of the host.

Acetonitrile proved essential for crystal formation and assembly stabilization. In the **G3** complex, a second guest molecule interacts via halogen bonds with two acetonitrile molecules (N···I distance of 3.07 Å and a mean C–I···N angle of 172.7°) forming a supramolecular aggregate in which **G3** adopts the expected staggered conformation. These aggregates are further organized into a two-dimensional network through C–N···π interactions^33^ between acetonitrile and pyrazine rings of adjacent allenophanes (Figure 4a). A similar scenario is observed **G4**@(*P*_4_)-**2**, where an additional guest molecule bridges an outer nitrogen atom and an acetonitrile molecule. In both cases, crystallization without acetonitrile failed, highlighting its essential role in maintaining the solid-state architecture. These examples underscore the ability of acetonitrile to effectively compete with the outer pyrazine nitrogen atoms—contrary to what has been reported for pyridine-based systems^34^—and reveal how structure guest and host flexibility, in tandem with solvent participation, govern the formation of halogen-bonded architectures.

## Conclusions

This work reports the synthesis of chiral hosts (*P*_4_)- and (*M*_4_)-**2** via an efficient three-step sequence. Computational and crystallographic analyses revealed an equilibrium among several conformers, with the twisted-boat form prevailing in the solid state. This conformation promotes self-assembly through aromatic interactions between pyrazine rings, forming extended supramolecular chains. Host–guest interactions with various halogen bond donors were studied by ^1^H–^15^N HMBC NMR, showing selective binding influenced by guest size, geometry, and host rigidity. Notably, guests like **G3** and **G4**, which fit the macrocyclic cavity well, showed strong binding. X-ray analysis confirmed halogen bond-driven encapsulation and highlighted how guest structure affects host distortion and packing. Remarkably, the otherwise unstable syn-gauche conformer of **G3** was stabilized and characterized through host–guest interactions. Acetonitrile played a key role in stabilizing these assemblies via secondary halogen and aromatic interactions. Overall, these findings highlight how guest structure, host flexibility, and crystallization conditions guide halogen-bonded architectures, offering design insights for future supramolecular systems with applications in selective recognition, separation, and sensing of halogenated compounds.

## Supplementary Material





## Data Availability

The data underlying this study are available in the published article and its .

## Notes

Experimental procedures, full characterization, X-ray data and computational details (PDF)
FAIR data, including the primary NMR FID files, for compounds **2**–**4** (ZIP)

## †

Departamento de Química Orgánica, Universidade de Santiago de Compostela, 15782 Santiago de Compostela, Spain
