# Supplementary material for: Shape-Matching and Halogen Bonding in Chiral Pyrazine-Allene Hosts: Confining an Unstable Guest Conformation
Source: Org Lett. 2025 Jun 24;27(26):7164–9. doi: 10.1021/acs.orglett.5c02075 (PMC12235685; doi:10.1021/acs.orglett.5c02075)
Supplement: Supplementary file 1 [file ol5c02075_si_001.pdf]

Shape-Matching and Halogen Bonding in Chiral Pyrazine-Allene Hosts: Confining an Unstable Guest Conformation

Víctor Rubio-Pisabarro, Jonathan Álvarez-García and María Magdalena Cid[†]*

Departamento de Química Orgánica, Edificio Ciencias Experimentais, Universidade de Vigo, E-36310, Spain.

[†]Departamento de Química Orgánica, Universidade de Santiago de Compostela, 15782 Santiago de Compostela, Spain.

Table of Contents

1. General methods 3

2. Synthesis 4

3. X-ray data 12

4. Computational details 26

   4.1. Computed ECD vs Experimetal ECD 78

5. Titration procedures 79

6. References 82

1. General methods

All reactions were carried out under nitrogen atmosphere unless otherwise stated. Reactions that required anhydrous conditions were carried out in oven-dried glassware at 120 °C for at least 24 hours. The reaction flasks were further dried by heating and subsequent cooling under a stream of nitrogen. The transfer of solvents or anhydrous solutions was carried out using syringes or cannulas, dried as described and stored in a desiccator with potassium hydroxide. Solvents were dried according to published methods and distilled before use.[1] Triethylamine was freshly distilled from CaH_2 under argon atmosphere. All other reagents were commercial compounds of the highest purity available. Silica gel 60F-254 Merck was used for thin layer chromatography and were visualized by exposure to UV light (254 nm) and revealed by treatment with a solution of phosphomolybdic acid or potassium permanganate. Merck silica gel 60 (230-240 mesh) was used under pressure for flash column chromatography.

^{1}H-NMR spectra were recorded at 25 ^{o}C (unless otherwise stated) on Bruker AMX-400 at 400 MHz with residual protic solvent as internal reference [$CDCl_3$, δ_H = 7.26 ppm]. Chemical shifts (δ) are given in parts per million (ppm) and coupling constants (J) are given in Hertz (Hz). The proton spectra are reported as follows: chemical shift δ (multiplicity, coupling constant *J*, number of protons). The following symbols were used for the description of coupling patterns: multiplet (m), singlet (s), doublet (d), triplet (t). ^{13}C-NMR spectra were recorded on the same spectrometer at 100 MHz at 25 ^{o}C (unless otherwise stated) with residual protic solvent as internal reference [$CDCl_3$, δ = 77.16 ppm]. ECD and UV-vis spectra were recorded on a Jasco J-815 spectropolarimeter using a one-centimetre thick quartz cuvette at 25 °C. The background was always obtained against the solvent. ESI mass spectra were recorded with an APEX3 instrument. Ions were generated using a Combi MALDI-- electrospray ionization (ESI) source. High-resolution mass spectra were taken on a VG Autospec instrument.

Crystallographic data were collected at 100 K using a Bruker D8 Venture diffractometer with a Photon II CMOS detector and Mo-Kα radiation (λ = 0.71073 Å) generated by an Incoatec high brillance microfocus source equipped with Incoatec Helios multilayer optics. The software APEX4[2] was used for collecting frames of data, indexing reflections, and determination of lattice parameters, SAINT[3] for integration of intensity of reflections, and SADABS[4] for scaling and empirical absorption correction. The structure was solved by dualspace algorithm using the program SHELXT.[5] All non-hydrogen atoms were refined with anisotropic displacement parameters by full-matrix least-squares calculations on F^2 using the program SHELXL[6] with OLEX2[7]. Hydrogen atoms were inserted at calculated positions and constrained with isotropic displacement. Drawings were produced with PLATON.[8]

2. Synthesis

(6*P*,6'*P*)-9,9'-(Pyrazine-2,6-diyl)bis(5,7-di-*tert*-butyl-2-methylnona-5,6-dien-3,8-diyn-2-ol), (*P*$_2$)-3

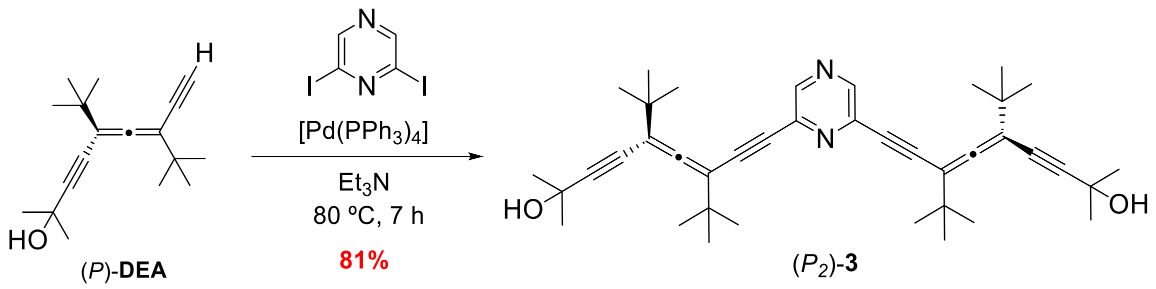

A solution of (*P*)-**DEA** (110 mg, 0.43 mmol) in 4.3 mL of Et_3N was transferred *via* cannula to a Schlenk tube. Commercial 2,6-diiodopyrazine (70 mg, 0.21 mmol) and $Pd(PPh_3)_4$ (25 mg, 0.021 mmol) were added and the reaction mixture was stirred at 80 °C in an oil bath for 7 h. The reaction mixture was then partitioned between saturated aqueous NH_4Cl solution and dichloromethane (DCM). The aqueous phase was extracted with DCM (5 mL x 5), the combined organic phases were dried with Na_2SO_4 and the solvent was removed under reduced pressure. Flash chromatography purification on silica gel (hexane/AcOEt 75:25) afforded (*P*$_2$)-**3** (102 mg, 81%) as an amber oil.

^{1}H NMR (400 MHz, $CDCl_3$) δ (ppm) 8.51 (s, 2H), 1.56 (s, 12H), 1.20 (s, 18H), 1.14 (s, 18H).

^{13}C NMR (100 MHz, $CDCl_3$) δ (ppm) 212.5, 145.4, 140.1, 103.9, 102.4, 98.3, 88.6, 88.2, 75.3, 65.9, 35.7, 31.6, 29.2, 29.0.

HRMS (ESI-TOF) m/z: $[M+H]^+$ calc. for $C_{40}H_{53}N_2O_2$ 593.4102; found 593.4118.

$[\alpha]_D^{23}$= +378 (c= 4.35 E^{-3} g/mL, $CHCl_3$).

UV-vis ($CHCl_3$): λ (ε): 289 (19684 $Lmol^{-1}cm^{-1}$), 343 (19479 $Lmol^{-1}cm^{-1}$) nm.

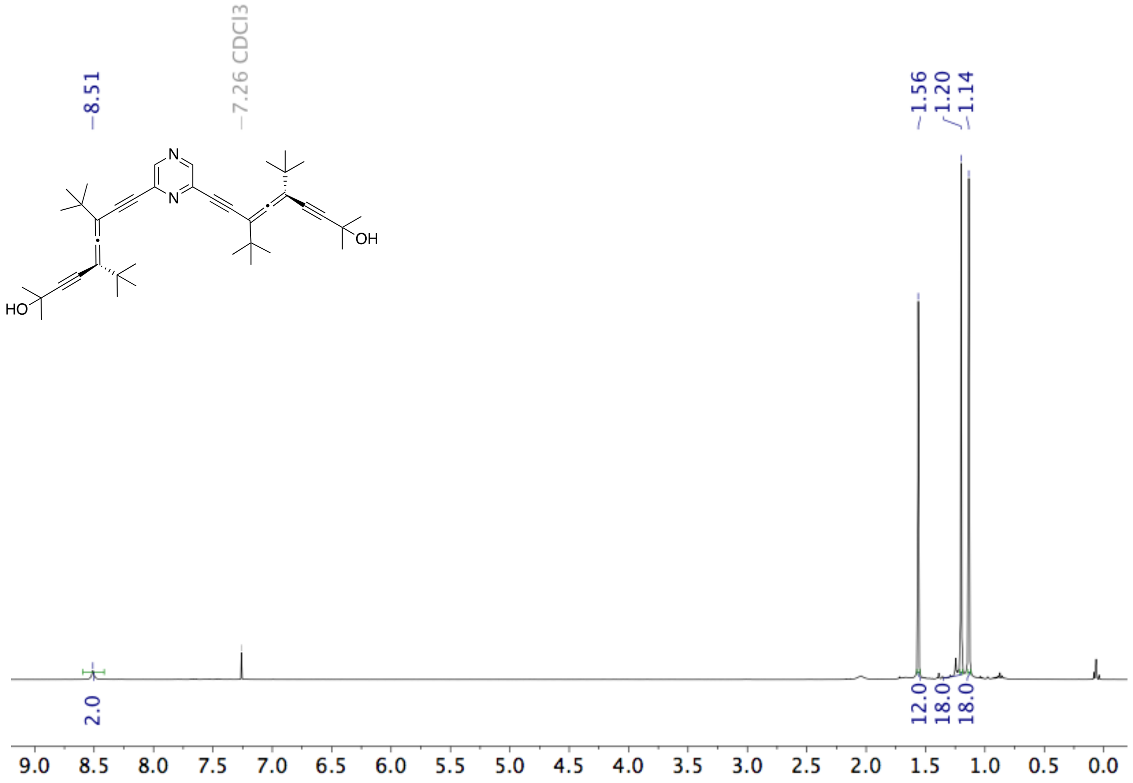

Figure S1.[1]*H NMR (400 MHz) spectrum of (P*$_2$*)-**3** in CDCl*$_3$*.*

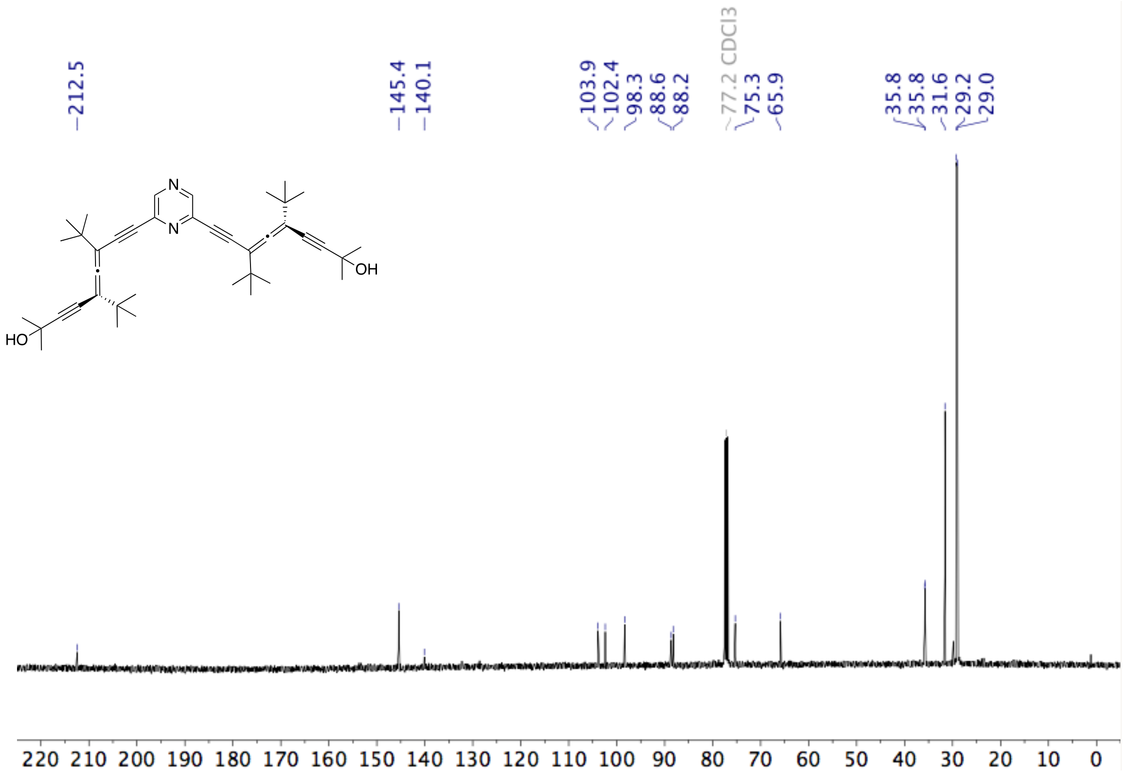

Figure S2.[13]*C NMR (100 MHz) spectrum of (P*$_2$*)-**3** in CDCl*$_3$*.*

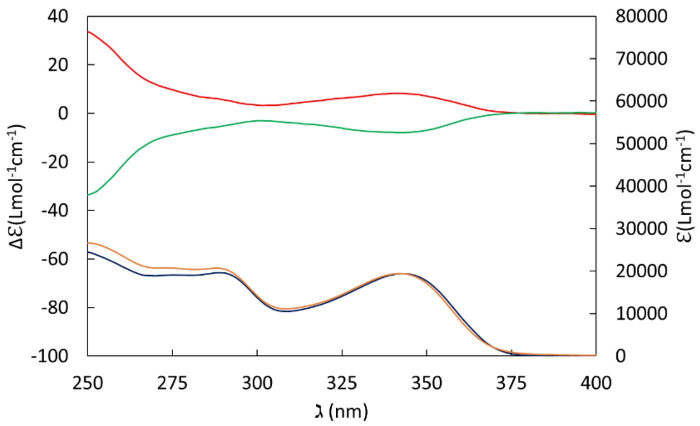

Figure S3. *UV-vis (blue and orange lines) – ECD (red and green lines) spectra of (P$_2$)-**3** and (M$_2$)-**3**, respectively (2.60 x 10^{-5} M in chloroform).*

2,6-Bis((*P*)-3,5-di-*tert*-butylhepta-3,4-dien-1,6-diyn-1-yl)pyrazine, (*P*$_2$)-4

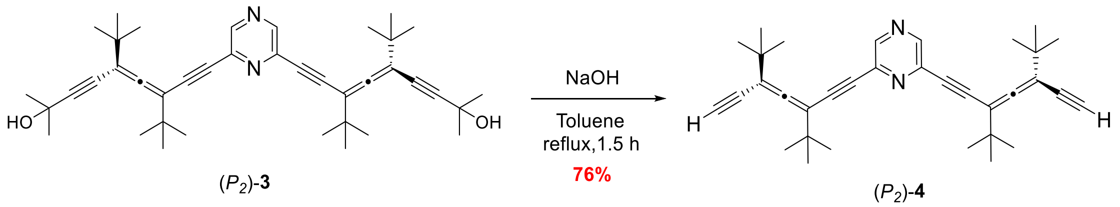

Pulverized NaOH (3.44 g, 86.0 mmol) was placed in a two necked round bottomed flask connected to a reflux condenser and dried with a heat gun under nitrogen flow. Then, a solution of (*P*$_2$)-**3** (102 mg, 0.17 mmol) in 27.5 mL of toluene was transferred *via* cannula and the reaction mixture was refluxed in an oil bath for 90 min. Then, saturated aqueous NH_4Cl solution was added (20 mL), the mixture was extracted with AcOEt (15 mL x 4), the combined organic phases were dried with Na_2SO_4 and the solvent was removed under reduced pressure. Flash chromatography purification on silica gel (hexane/dichloromethane 6:4) afforded (*P*$_2$)-**4** (62 mg, 76%) as an amber oil.

^{1}H NMR (400 MHz, $CDCl_3$) δ (ppm) 8.51 (s, 2H), 3.05 (s, 2H), 1.21 (s, 18H), 1.16 (s, 18H).

^{13}C NMR (100 MHz, $CDCl_3$) δ (ppm) 213.1, 145.6, 140.0, 103.6, 102.9, 88.9, 87.8, 81.6, 77.0, 35.9, 35.6, 29.2, 28.9.

HRMS (ESI-TOF) m/z: $[M+H]^+$ calc. for $C_{34}H_{41}N_2$ 477.3264; found 477.3261.

$[\alpha]_D^{24}$= +354 (c= 1.47 E^{-3} g/mL, $CHCl_3$).

UV-vis ($CHCl_3$): λ (ε): 286 (21377 Lmol^{-1}cm^{-1}), 342 (21227 Lmol^{-1}cm^{-1}) nm.

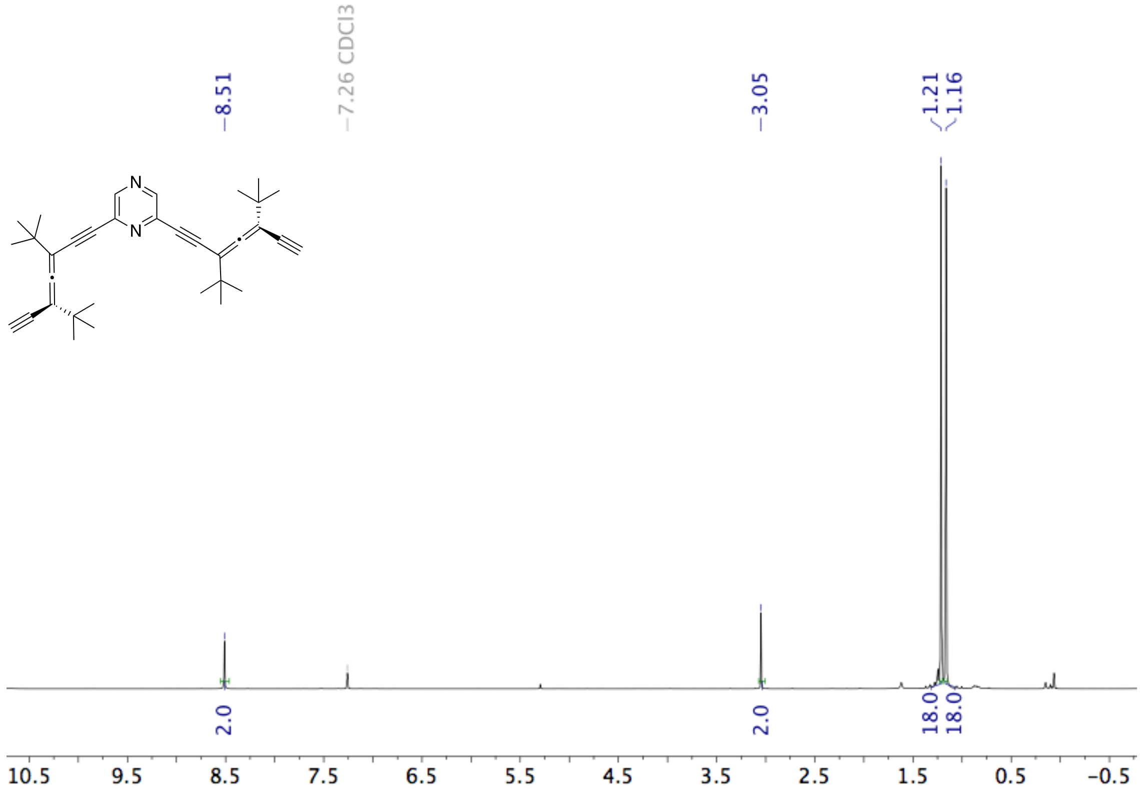

Figure S4. *^{1}H NMR (400 MHz) spectrum of (P_2)-**4** in $CDCl_3$.*

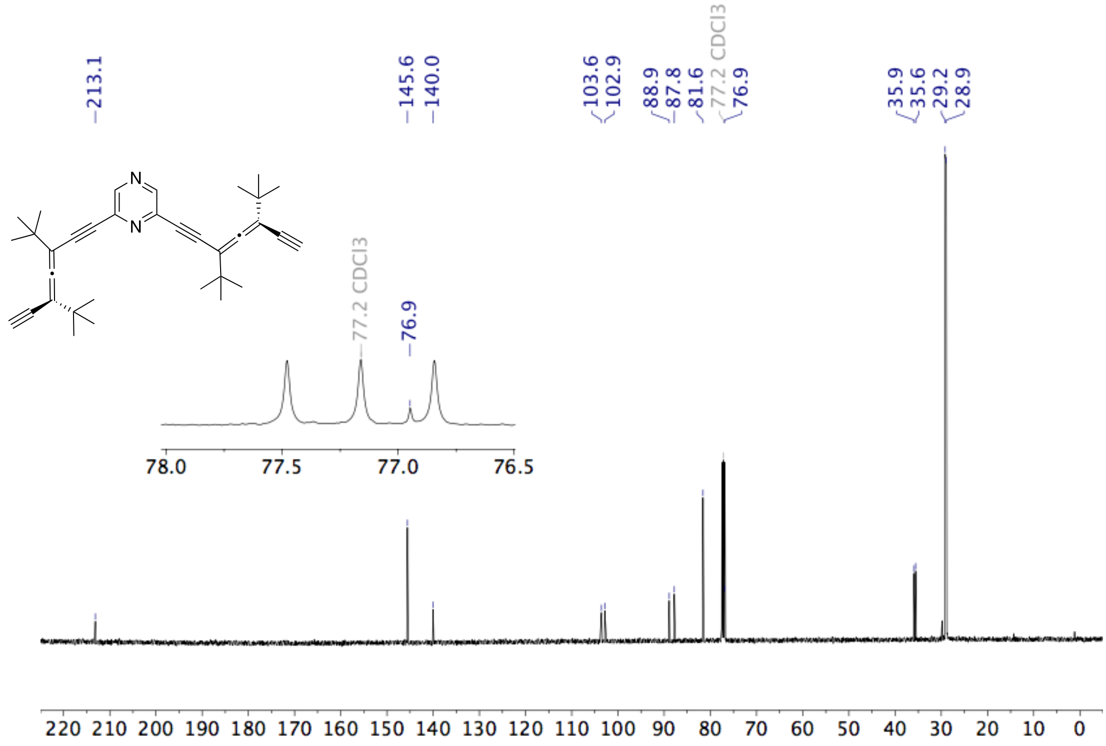

Figure S5. *^{13}C NMR (100 MHz) spectrum of (P_2)-**4** in $CDCl_3$.*

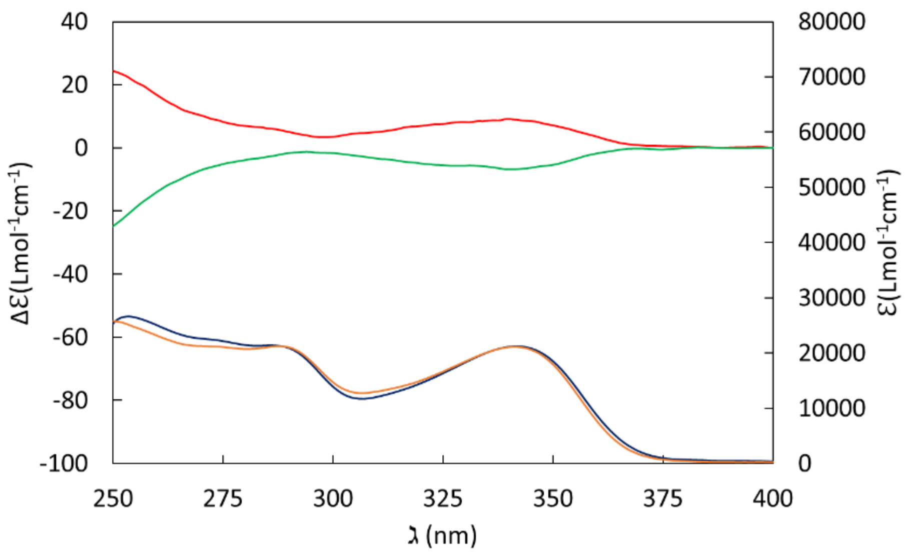

Figure S6. *UV-vis (blue and orange lines) – ECD (red and green lines) spectra of (P*$_2$*)-**4** and (M*$_2$*)-**4**, respectively (2.60 x 10*$^{-5}$ *M in chloroform).*

(*P*,*P*,*P*,*P*)-4,6,11,13,19,21,26,28-Octa-*tert*-butyl 1,16(2,6)dipyrazinecyclotriacontaphane - 4,5,11,12,19,20,26,27-octaen - 2,7,9,10,14,17,22,24,29-octayne, (*P*$_4$)-2

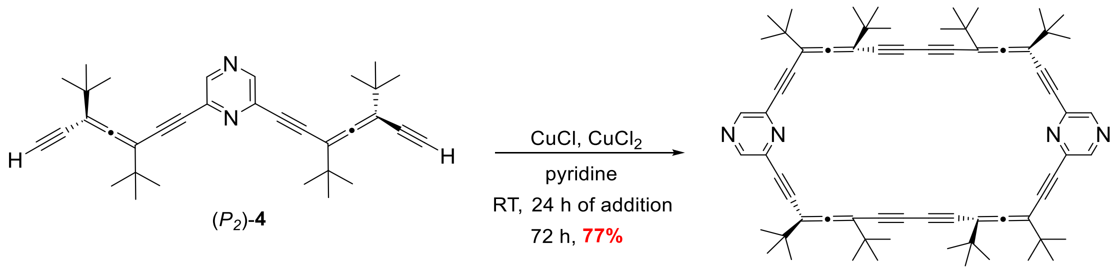

Two solutions were prepared:

Solution *a*: CuCl (218 mg, 2.20 mmol) and $CuCl_2$ (43 mg, 0.32 mmol) were placed in a round-bottomed flask under Ar atmosphere, dissolved in dry pyridine (12 mL) and the solution was degassed by bubbling Ar for 30 min.

Solution *b*: (*P*$_2$)-**4** (14 mg, 0.029 mmol) was dissolved in dry pyridine (16 mL) and the solution was degassed by bubbling Ar for 30 min.

Solution *b* was added to **solution *a*** by means of an automatic syringe (rate: 0.6 mL/h) while stirring. After 72 h, the solvent was removed under reduced pressure, the remaining solid was dissolved in AcOEt (20 mL) and washed with a saturated aqueous KCN solution (20 mL), the aqueous phase was extracted with AcOEt (15 mL x 3), the combined organic phases were dried with Na_2SO_4 and the solvent was removed under reduced pressure. Flash chromatography purification on silica gel (hexane/dichloromethane 1:1) afforded (*P*$_4$)-**2** (11 mg, 77%) as a white solid.

^{1}H NMR (400 MHz, $CDCl_3$) δ (ppm) 8.49 (s, 4H), 1.22 (s, 36H), 1.17 (s, 36H).

^{13}C NMR (100 MHz, $CDCl_3$) δ (ppm) 215.2, 145.3, 140.0, 104.2, 103.2, 89.2, 87.5, 78.3, 75.1, 36.2, 35.8, 29.2, 29.1.

HRMS (ESI-TOF) m/z: $[M+H]^+$ calc. for $C_{68}H_{77}N_4$ 949.6142; found 949.6150.

$[\alpha]_D^{23}$= +250 (c= 1.08 E^{-3} g/mL, $CHCl_3$).

UV-vis ($CHCl_3$): λ (ε): 285 (78516 $Lmol^{-1}cm^{-1}$), 300 (86780 $Lmol^{-1}cm^{-1}$), 346 (37350 $Lmol^{-1}cm^{-1}$) nm.

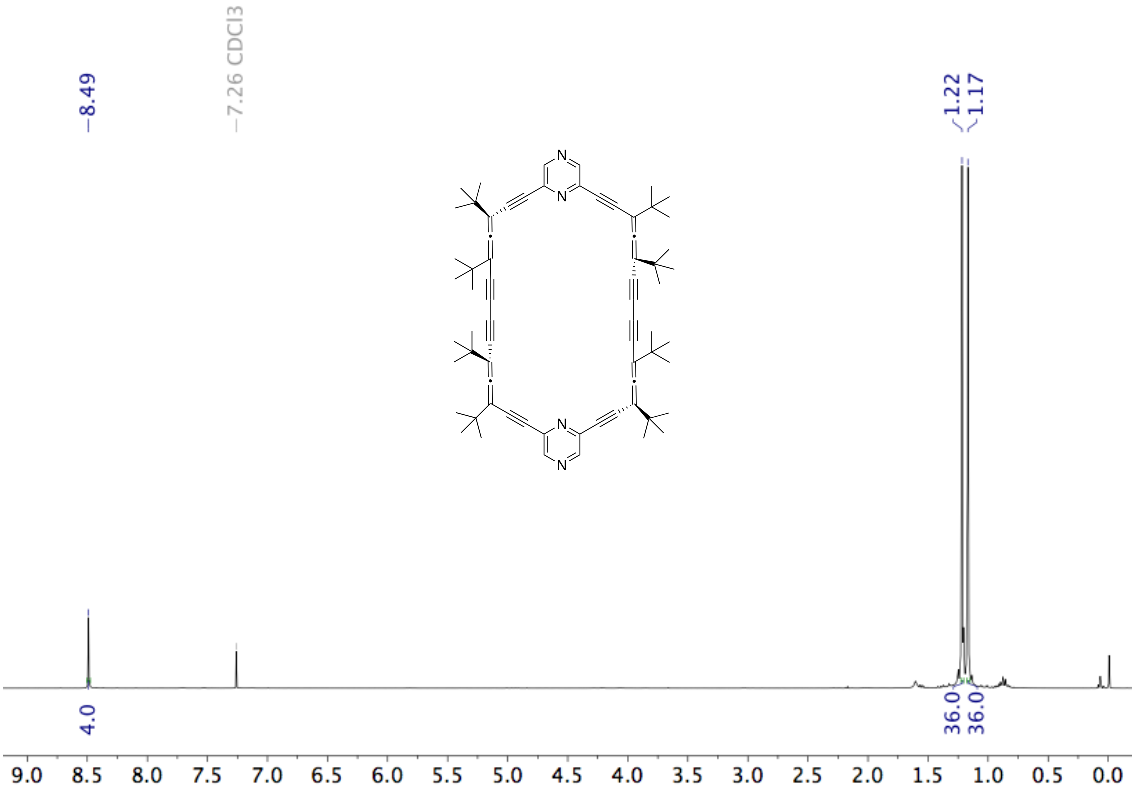

Figure S7.^{1}*H NMR (400 MHz) spectrum of (P_4)-**2** in $CDCl_3$.*

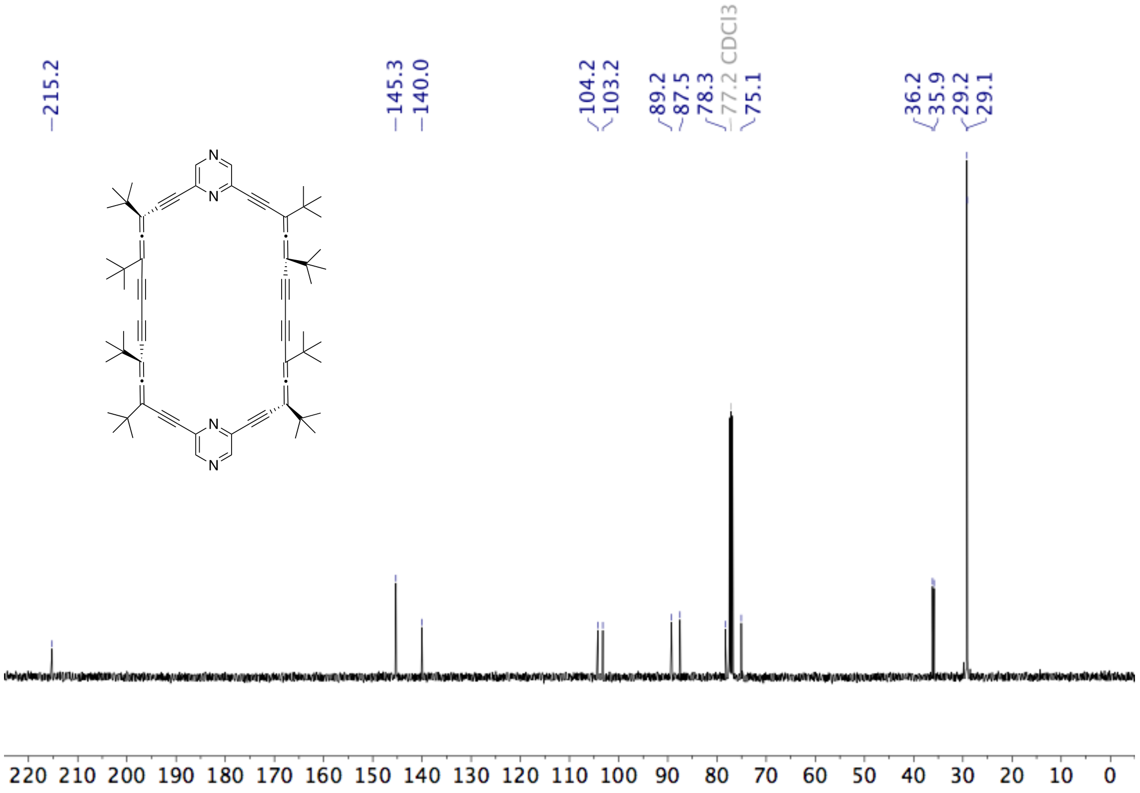

Figure S8.[13]*C NMR (100 MHz) spectrum of (P*$_4$*)-**2** in CDCl*$_3$*.*

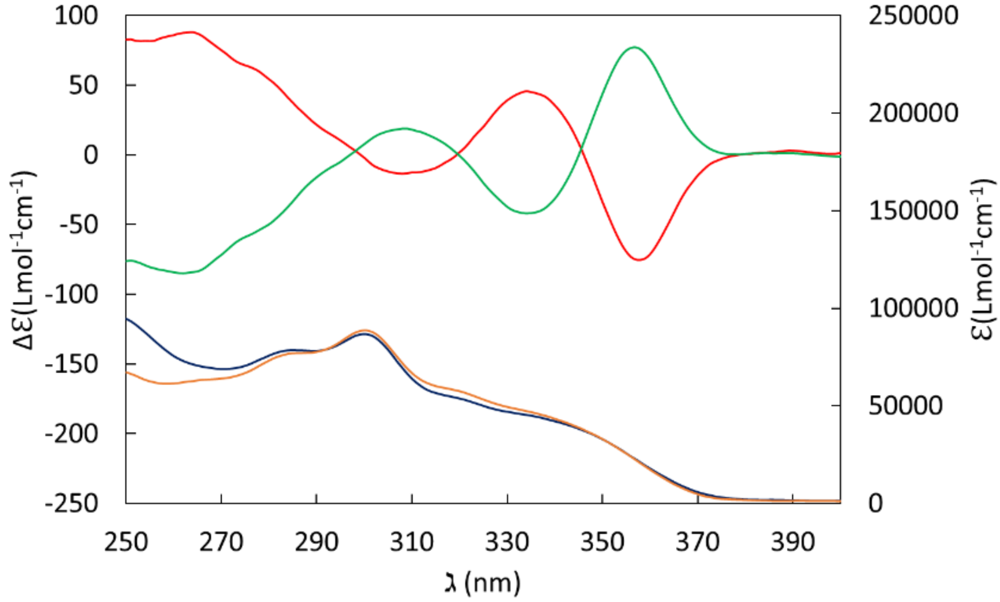

***Figure S9.** UV-vis (blue and orange lines) – ECD (red and green lines) spectra of (P*$_4$*)-**2** and (M*$_4$*)-**2**, respectively (6.24 x 10*$^{-6}$ *M in chloroform).*

1,4-Diethynyl-2,3,5,6-tetrafluorobenzene, 5

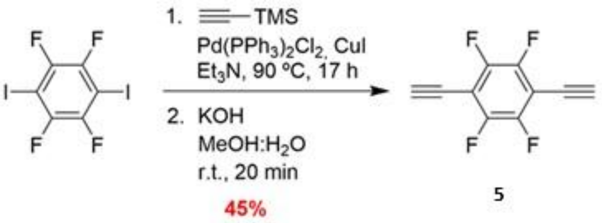

1,4-Diiodotetraflurobenzene (1.0 g, 2.49 mmol), $Pd(PPh_3)_2Cl_2$ (175 mg, 0.25 mmol) and CuI (47 mg, 0.25 mmol) were suspended in Et_3N (25 mL) in a Schlenk tube and the mixture was degassed by bubbling N_2 for 15 min. Then, trimethylsilylacetylene (860 µL, 6.22 mmol) was added dropwise and the mixture was stirred at 90 °C in an oil bath for 17 h. The mixture was then diluted with dichloromethane (25 mL) and filtered through a celite pad. Saturated aqueous NH_4Cl solution was added (25 mL), the aqueous phase was extracted with DCM (15 mL x 5), the combined organic phases were dried with Na_2SO_4 and the solvent was removed under reduced pressure. The product was dissolved in methanol (13.5 mL), KOH (70 µL, 0.5 M solution in water) was added and the reaction was stirred at room temperature for 20 min. The mixture was diluted with water and the aqueous phase was extracted using pentane (15 mL x 5). Then, the combined organic phases were dried with Na_2SO_4 and the solvent was removed under reduced pressure. Flash chromatography purification on silica gel (dichloromethane) afforded **5** (222 mg, 45%).

^{1}H NMR (400 MHz, $CDCl_3$) δ (ppm) 3.45 (s, 2H).

The ^{1}H NMR spectrum is in good agreement with published data.[9]

1,2,4,5-Tetrafluoro-3,6-bis(iodoethynyl)benzene, G2

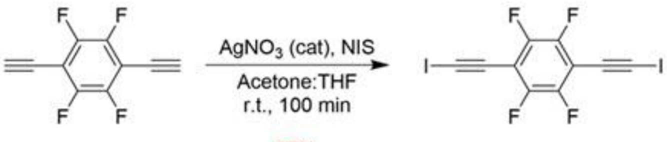

5 G2

$AgNO_3$ (88 mg, 0.52 mmol) was added to a suspension of **5** (222 mg, 1.12 mmol) in a 1:1 mixture of acetone-THF (48 mL). Then, N-iodosuccinimide (554 mg, 2.46 mmol) was added and the mixture was stirred for 100 min. The reaction mixture was filtered through cotton and the solvent was removed under reduced pressure. Flash chromatography purification on silica gel (petroleum ether) afforded **G2** (336 mg, 67%).

^{13}C NMR (100 MHz, $CDCl_3$) δ (ppm) 149.0, 145.7, 78.8, 23.9.

^{19}F NMR (379 MHz, $CDCl_3$) δ (ppm) -136.8.

The ^{13}C and ^{19}F NMR spectra are in good agreement with published data.[10]

3. X-ray data

(*P*$_4$)-2

XRD quality crystals were obtained by slow cooling of a solution of (*P*$_4$)-**2** in ethanol.[1]

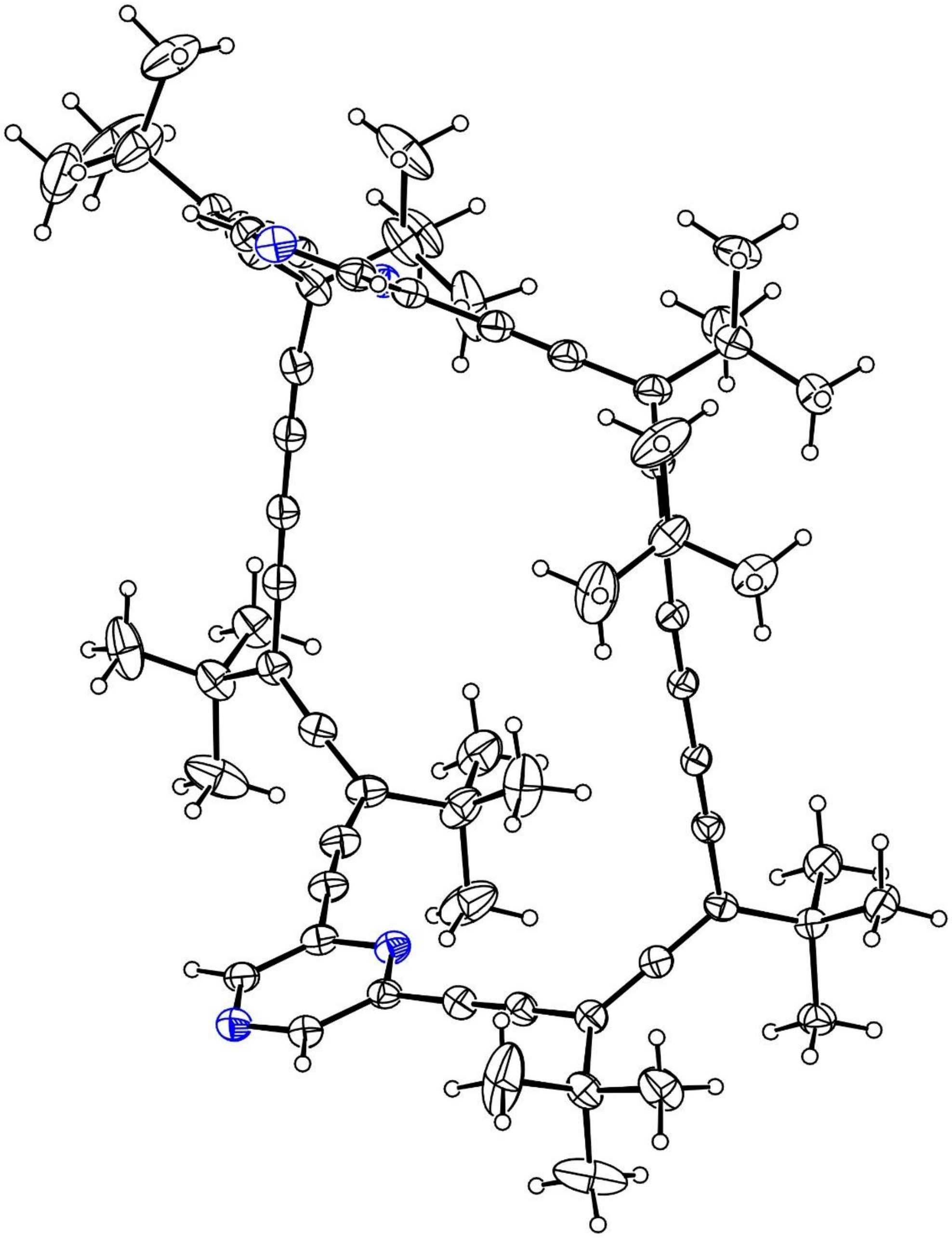

Figure S10a. *Thermal ellipsoid plot for (P$_2$)-**4** x-ray structure (50% probability).*

[1] CCDC 2440451 contains the supplementary crystallographic data for (*P*$_2$)-**4**.

Empirical formula	C68 H76 N4
Formula weight	949.32
Temperature	100.00 K
Wavelength	0.71073 Å
Crystal system	Triclinic
Space group	P1
Unit cell dimensions	a = 13.4132(15) Å α= 75.390(3)°.
	b = 16.0135(17) Å β= 71.564(3)°.
	c = 16.6104(18) Å γ = 72.215(3)°.
Volume	3174.7(6) Å^3
Z	2
Density (calculated)	0.993 Mg/m^3
Absorption coefficient	0.057 mm^{-1}
F(000)	1024
Crystal size	0.093 x 0.071 x 0.044 mm^3
Theta range for data collection	2.043 to 26.370°.
Index ranges	-16<=h<=16, -20<=k<=20, -20<=l<=20
Reflections collected	125722
Independent reflections	25904 [R(int) = 0.0613]
Completeness to theta = 25.242°	99.9%
Absorption correction	Semi-empirical from equivalents
Max. and min. transmission	0.7080 and 0.6521
Refinement method	Full-matrix least-squares on F^2
Data / restraints / parameters	25904 / 33 / 1345
Goodness-of-fit on F^2	1.041
Final R indices [I>2sigma(I)]	R1 = 0.0819, wR2 = 0.2186
R indices (all data)	R1 = 0.0960, wR2 = 0.2327
Absolute structure parameter	-1.3(10)
Extinction coefficient	n/a
Largest diff. peak and hole	0.504 and -0.286 e.Å^{-3}

The structure consists of two macrocyclic molecules, both adopting a boat conformation, although they are crystallographically independent. The molecular nature enables the formation of self-assembled chains through π···π interactions along the *b*-axis (**Figure S10b**). Each pyrazine ring partially interacts with an analogous ring from a neighboring molecule, with only two atoms of each ring overlapping. The distances between the involved atoms range from 3.196(6) to 3.430(6) Å, while the dihedral angles between planes are between 13.5(2)° and 17.4(2)°.

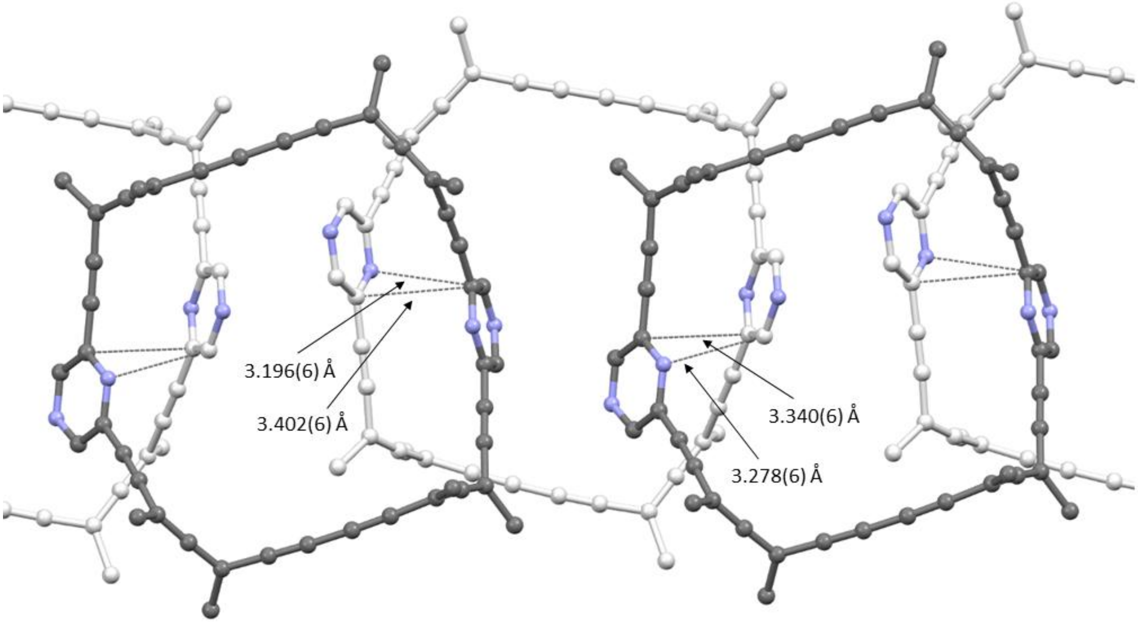

Figure S10b. *Self-assembled chains of (P$_4$)-**2** through π···π interactions along the b-axis.*

1,4-Diiodooctafluorobutane@(P_4)-2

XRD quality crystals were obtained through liquid diffusion layering of acetonitrile into a solution of (P_4)-**2** and 1,4-diiodooctafluorobutane in C_6D_6.[2]

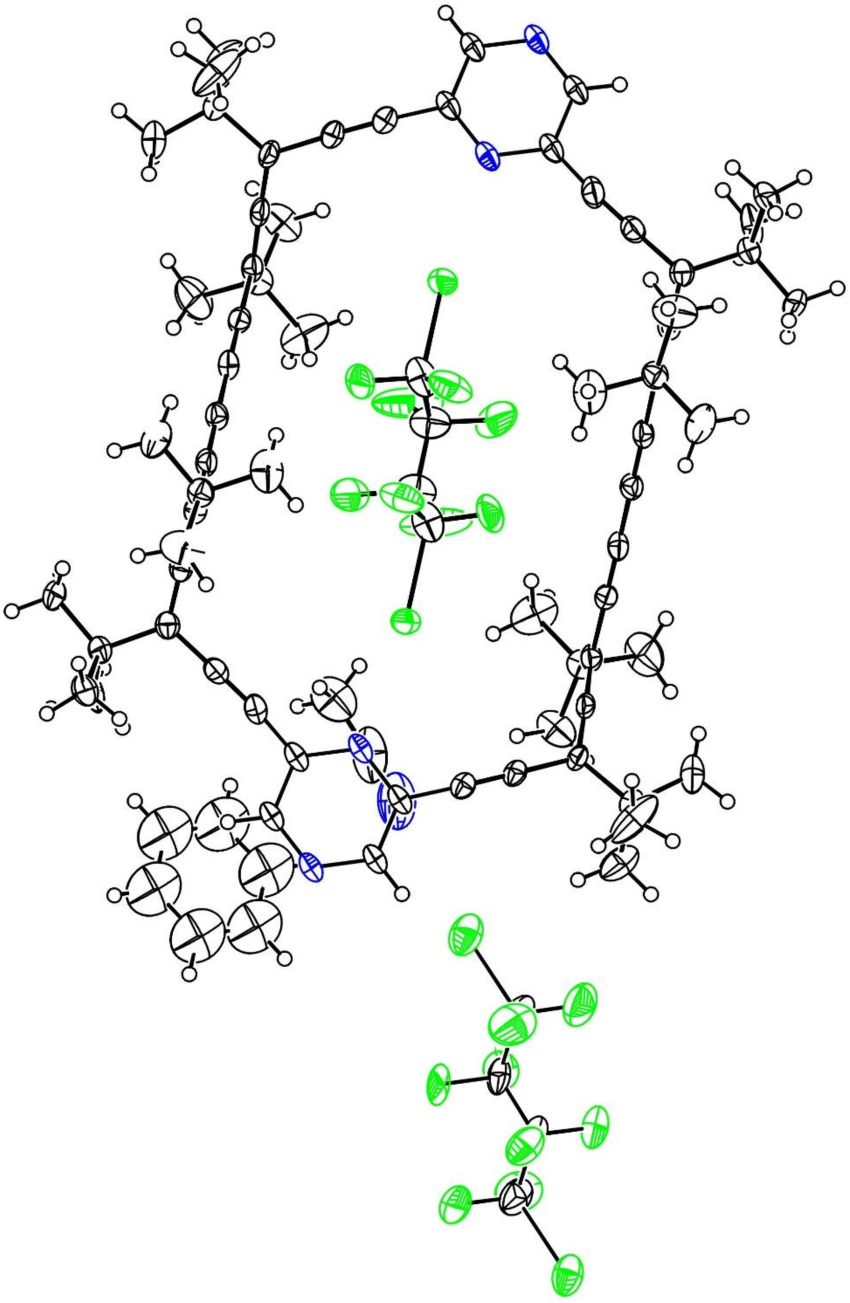

Figure S11a. *Thermal ellipsoid plot for complex 1,4-Diiodooctafluorobutane@(P₄)-**2** x-ray structure (50% probability).*

[2] CCDC 2440456 contains the supplementary crystallographic data for 1,4-Diiodooctafluorobutane@(P_4)-**2**.

Empirical formula	C86 H88 F16 I4 N6	
Formula weight	2017.22	
Temperature	100.00 K	
Wavelength	0.71073 Å	
Crystal system	Monoclinic	
Space group	P 1 2 1	
Unit cell dimensions	a = 16.2293(19) Å	α= 90°.
	b = 6.8997(8) Å	β= 96.261(4)°.
	c = 20.159(2) Å	γ = 90°.
Volume	2243.9(5) $Å^3$	
Z	1	
Density (calculated)	1.493 Mg/m^3	
Absorption coefficient	1.466 mm^{-1}	
F(000)	1002	
Crystal size	0.242 x 0.135 x 0.036 mm^3	
Theta range for data collection	2.033 to 28.348°.	
Index ranges	-21<=h<=21, -9<=k<=9, -26<=l<=26	
Reflections collected	110672	
Independent reflections	11201 [R(int) = 0.0436]	
Completeness to theta = 25.242°	99.9%	
Absorption correction	Semi-empirical from equivalents	
Max. and min. transmission	0.7457 and 0.5896	
Refinement method	Full-matrix least-squares on F^2	
Data / restraints / parameters	11201 / 90 / 537	
Goodness-of-fit on F^2	1.062	
Final R indices [I>2sigma(I)]	R1 = 0.0806, wR2 = 0.1979	
R indices (all data)	R1 = 0.0825, wR2 = 0.1990	
Absolute structure parameter	0.131(9)	
Extinction coefficient	n/a	
Largest diff. peak and hole	1.093 and -1.927 e.$Å^{-3}$	

The structure consists of macrocyclic molecules, 1,4-diiodoperfluorobutane molecules, acetonitrile, and benzene. The molecular nature of these components enables the formation of self-assembled architectures through halogen bond interactions (**Table S1**), C-N···π interactions (**Table S2**), and C-H···π interactions (**Table S3**).

Initially, a halogen-bonded complex is formed, in which a halogenated molecule is encapsulated within the macrocycle (**Table S1**, **Figure S11b**). Additionally, a second halogenated molecule

establishes halogen bonds with two acetonitrile molecules, forming a molecular aggregate (**Table S1, Figure S12**). C-N···π interactions, involving acetonitrile molecules and the pyrazine rings of the macrocycles, link the initial discrete assemblies into a two-dimensional organization (**Table S2a, Figure S13**). Finally, benzene molecules are positioned between the resulting layers, establishing C-H···π interactions with the pyrazine rings, leading to the formation of a three-dimensional arrangement (**Table S2b, Figure S14**).

Table S1. Halogen interaction parameters (Å,°)

**C-I···N**	**d(I···N)**	**θ** [∠(C-I···N)]
C33-I1···N1B	2.914(11)	170.5(5)
C33-I1···N1	2.983(15)	168.4(6)
C35-I48···N3	3.289(18)	169.1(13)
C38-I48···N3_$1	2.850(18)	176.4(11)
$1 2-x,y,-z		

Table 2a. C-N···π interaction parameters (Å,°)

**C-N···π**	**d[N···Cg(J)]**	**d[N···⊥P(J)]**	**γ**	**∠[C-N···Cg(J)]**
C39-N3···Cg(1)	3.56(3)	3.280	22.84	85.3(19)
C39-N3···Cg(2)_$2	3.48(3)	3.390	13.38	90.0(19)

Cg(1): N1B-C2B-C3B-N4B-C5B-C6B; Cg(2): N1-C2-C3-N4-C5-C6; $2 x,1+y,z

Cg(J): Center of gravity of ring J.

d[N···Cg(J)]: Distance of N atom to Cg(J)

d[N···⊥P(J)]: Perpendicular distance of N atom to ring plane J.

γ: Angle between Cg(J)-H vector and ring J vector.

∠[C-N···Cg(J)]: C-N-Cg(J) angle.

Table 2b. C-H···π interaction parameters (Å,°)

**C-H···π**	**d[H···Cg(J)]**	**d[H···⊥P(J)]**	**γ**	**∠[C-H···Cg(J)]**	**d[C···Cg(J)]**
C42-H51···Cg(1)_$2	2.93	2.85	13.84	127	3.58(3)
C45-H54···Cg(2)_$3	2.94	2.89	9.86	134	3.67(3)

Cg(1): N1B-C2B-C3B-N4B-C5B-C6B; Cg(2): N1-C2-C3-N4-C5-C6; $2 x,1+y,z; $3 1-x,y,-z

Cg(J): Center of gravity of ring J.

d[H···Cg(J)]: Distance of H atom to Cg(J)

d[H···⊥P(J)]: Perpendicular distance of H atom to ring plane J.

γ: Angle between Cg(J)-H vector and ring J vector.

∠[C-H···Cg(J)]: C-H-Cg(J) angle.

d[C···Cg(J)]: Distance of C atom to Cg(J).

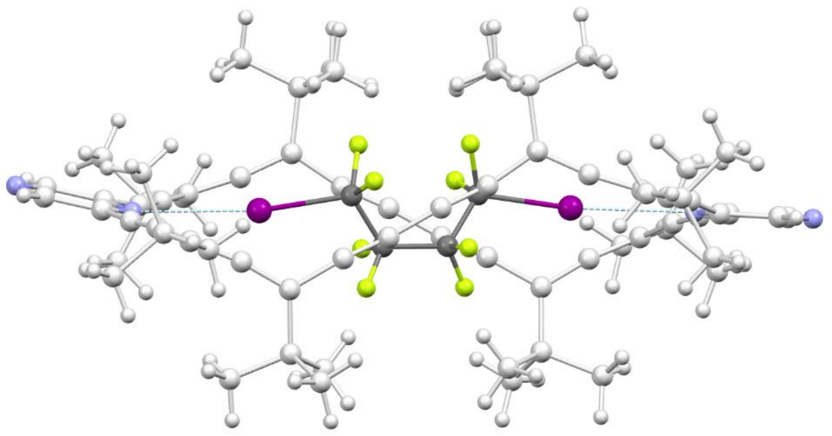

Figure S11b. *Complex 1,4-Diiodooctafluorobutane@(P_4)-**2***

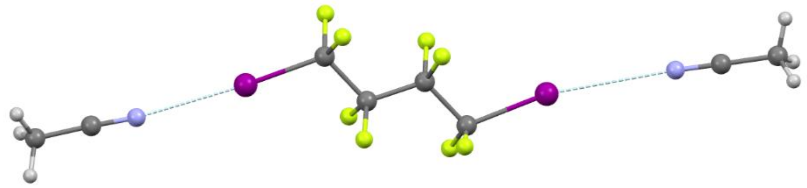

Figure S12. *Aggregate $ICF_2CF_2CF_2CF_2I$ + 2 acetonitrile.*

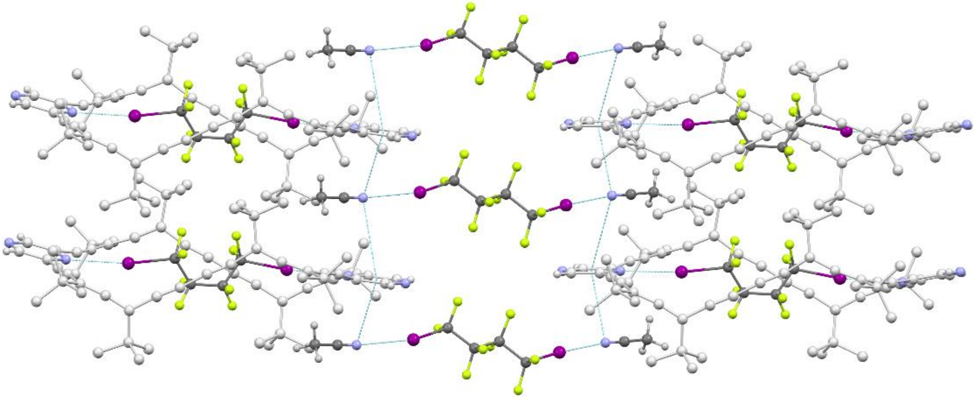

Figure S13. *Representation of the two-dimensional network.*

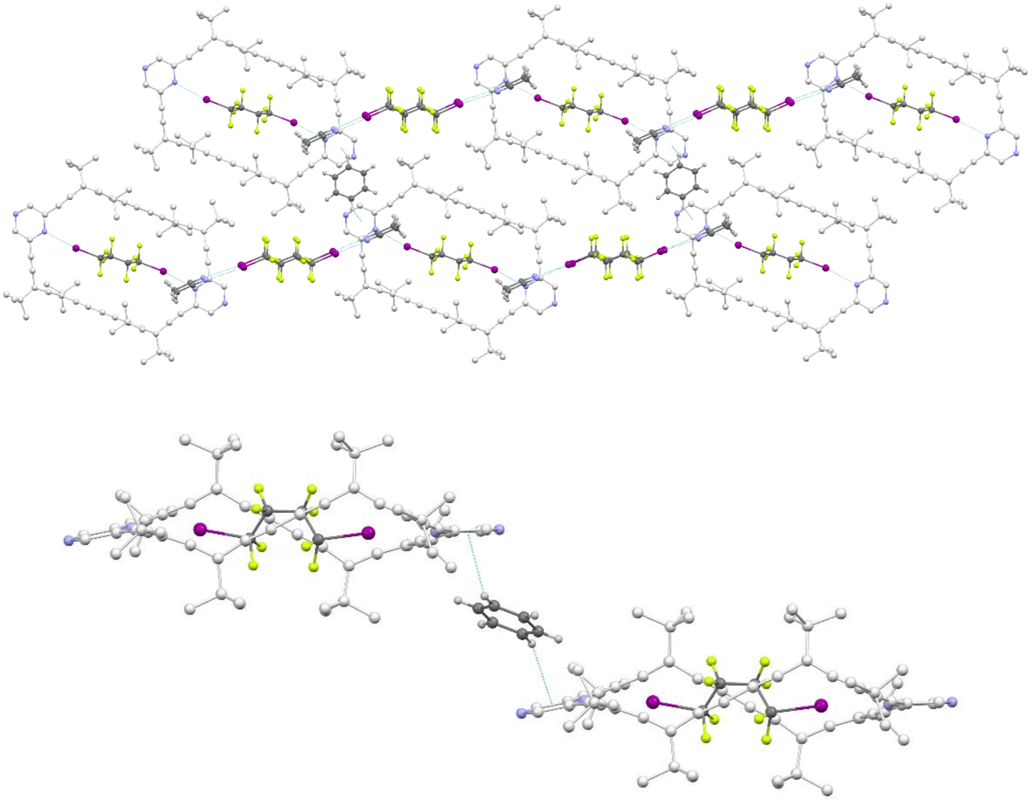

Figure S14. *Representation of the 3D organization and detail of the C-H···π interaction.*

1,4-Diiodotetrafluorobenzene@(*P*$_4$)-2

XRD quality crystals were obtained through liquid diffusion layering of acetonitrile into a solution of (*P*$_2$)-**2** and 1,4-diiodotetrafluorobenzene in C_6D_6.[3]

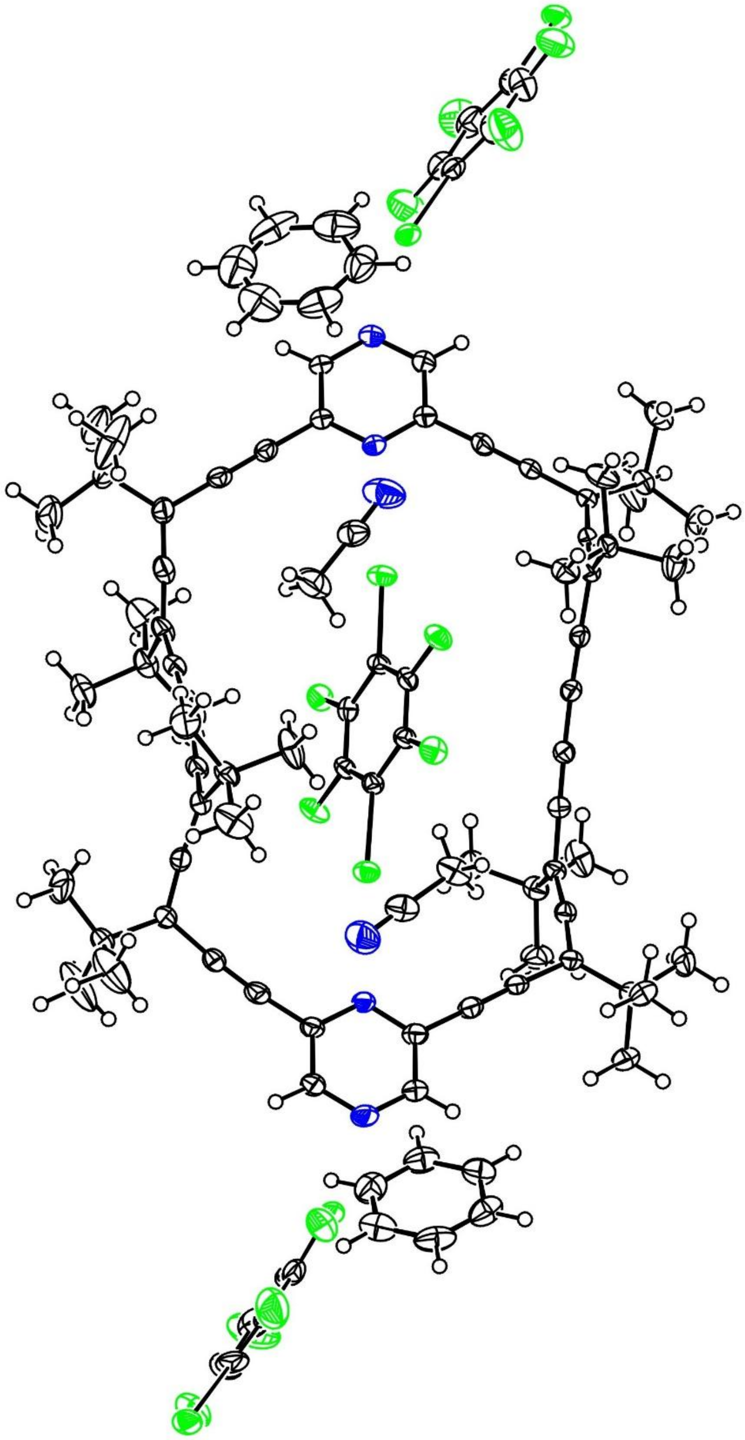

Figure S15a. *Thermal ellipsoid plot for complex 1,4-Diiodotetrafluorobenzene@(P$_4$)-**2** x-ray structure (50% probability).*

[3] CCDC 2440452 contains the supplementary crystallographic data for 1,4-Diiodotetrafluorobenzene @(*P*$_4$)-**2**.

Empirical formula	C102 H94 F12 I6 N6	
Formula weight	2393.23	
Temperature	100.00 K	
Wavelength	0.71073 Å	
Crystal system	Monoclinic	
Space group	C 1 2 1	
Unit cell dimensions	a = 54.418(5) Å	α = 90°.
	b = 7.1436(7) Å	β = 122.872(2)°.
	c = 31.065(3) Å	γ = 90°.
Volume	10142.8(17) $Å^3$	
Z	4	
Density (calculated)	1.567 Mg/m^3	
Absorption coefficient	1.907 mm^{-1}	
F(000)	4696	
Crystal size	0.217 x 0.134 x 0.111 mm^3	
Theta range for data collection	2.003 to 28.314°.	
Index ranges	-72<=h<=72, -9<=k<=9, -41<=l<=41	
Reflections collected	238513	
Independent reflections	25233 [R(int) = 0.0328]	
Completeness to theta = 25.242°	99.7%	
Absorption correction	Semi-empirical from equivalents	
Max. and min. transmission	0.7457 and 0.4853	
Refinement method	Full-matrix least-squares on F^2	
Data / restraints / parameters	25233 / 9 / 1210	
Goodness-of-fit on F^2	1.100	
Final R indices [I>2sigma(I)]	R1 = 0.0357, wR2 = 0.0909	
R indices (all data)	R1 = 0.0368, wR2 = 0.0916	
Absolute structure parameter	0.019(3)	
Extinction coefficient	n/a	
Largest diff. peak and hole	1.038 and -0.817 e.$Å^{-3}$	

The structure consists of macrocyclic molecules, 1,4-diiodotetrafluorobenzene molecules, acetonitrile, and benzene. The molecular nature enables the formation of self-assembled architectures through halogen bond interactions (**Tables S3a** and **S3b**), C-N···π and C-F···π interactions (**Table S4a**), and C-H···π and π···π interactions (**Tables S4b** and **S4c**).

Initially, a halogen-bonded complex is formed, in which a halogenated molecule is encapsulated within the macrocycle (**Table S3a**, **Figure S15b**). Additionally, each "external" nitrogen atom of the pyrazine rings in the macrocycle is linked to another halogenated molecule, which in turn

interacts with an acetonitrile molecule, forming a molecular aggregate (**Table S3a**, **Figure S16**). C-F···F-C and C-F···π interactions involving the "external" halogenated molecules connect the initial discrete assemblies into a two-dimensional organization, further stabilized by C-N···π interactions (**Tables S3b** and **S4a**, **Figure S17**). Finally, benzene molecules are positioned between the resulting layers, establishing C-H···π interactions among them and linking to the pyrazine rings through π···π interactions, ultimately forming a three-dimensional arrangement (**Tables S4b** and **S4c**, **Figure S18**).

Table S3a. Halogen interaction parameters (Å,°)

**C-I···N**	**d(I···N)**	**θ** [∠(C-I···N)]
C73-I1···N1	3.062(5)	177.3(2)
C76-I2···N21	3.084(5)	175.0(2)
C94-I3···N4	2.875(5)	167.5(3)
C97-I4···N7_$1	2.896(8)	169.7(4)
C100-I5···N24	2.904(5)	167.5(2)
C103-I6···N8_$2	3.066(6)	170.8(3)
$1: -1/2+x, 1/2+y, -1+z; $2: 1/2+x, -1/2+y, 1+z		

Table S3b. Halogen interaction parameters (Å,°)

**C-F···F-C**	**d(F···F)**	**θ1**	**θ2**
C101-F9···F12-C105_$3	2.647(12)	149.5(8)	132.9(6)
C102-F10···F11-C104_$3	2.685(13)	135.0(6)	148.1(8)
C105-F12···F9-C101_$4	2.647(12)	132.9(6)	149.5(8)
C104-F11···F10-C102_$4	2.685(13)	148.1(8)	135.0(6)
C99-F8···F5-c95_$3	2.53(2)	132.0(10)	166.0(14)
C98-F7···F6-C96_$3	2.49(2)	171.4(14)	131.0(8)
C95-F5···F8-c99_$4	2.53(2)	166.0(14)	132.0(10)
C96-F6···F7-C98_$4	2.49(2)	131.0(8)	171.4(14)
$3: x, 1+y, z; $4: x, -1+y, z			

Table S4a. C-X···π interaction parameters (Å,°)

C-X···π	d[X···Cg(J)]	d[X···⊥P(J)]	γ	∠[C-X···Cg(J)]
C99-F8···Cg(3)_$1	3.27(2)	3.260	4.49	99.1(11)
C80-N7···Cg(2)	3.194(10)	3.098	14.06	110.3(7)
C78-N8···Cg(1)	3.651(9)	3.393	21.66	102.6(6)
Cg(1): N1-C2-C3-N4-C5-C6; Cg(2): N21-C22-C23-N24-C25-C26; Cg(3): C100-C101-C102-C103-C104-C105<br>$1: -1/2+x, 1/2+y, -1+z				

Cg(J): Center of gravity of ring J.

d[X···Cg(J)]: Distance of X atom to Cg(J)

d[X···⊥P(J)]: Perpendicular distance of X atom to ring plane J.

γ: Angle between Cg(J)-H vector and ring J vector.

∠[C-X···Cg(J)]: C-X-Cg(J) angle.

Table S4b. C-H···π interaction parameters (Å,°)

C-H···π	d[H···Cg(J)]	d[H···⊥P(J)]	γ	∠[C-H···Cg(J)]	d[C···Cg(J)]
C84-H84···Cg(4)_$5	2.79	2.78	5.17	143	3.600(9)
C91-H91···Cg(5)_$6	2.92	2.90	6.72	139	3.682(12)
Cg(4): C82-C83-C84-C85-C86-C87; Cg(5): C88-C89-C90-C91-C92-C93; $5: -1/2-x, 1/2+y, -1-z; $6: 1/2-x, -1/2+y, -z					

Cg(J): Center of gravity of ring J.

d[H···Cg(J)]: Distance of H atom to Cg(J)

d[H···⊥P(J)]: Perpendicular distance of H atom to ring plane J.

γ: Angle between Cg(J)-H vector and ring J vector.

∠[C-H···Cg(J)]: C-H-Cg(J) angle.

d[C···Cg(J)]: Distance of C atom to Cg(J).

Table S4c. π···π interaction parameters (Å,°)

π···π	d[Cg(I)-Cg(J)]	d[Cg(I)···⊥P(J)]	d[⊥P(I)···Cg(J)]	α	β	γ
Cg(1)···Cg(4)	4.043(5)	3.901(3)	3.134(3)	26.9(4)	39.2	15.2
Cg(2)···Cg(5)	3. 842(6)	3.670(3)	3.283(4)	16.7(4)	31.3	17.2
Cg(1): N1-C2-C3-N4-C5-C6; Cg(2): N21-C22-C23-N24-C25-C26; Cg(4): C82-C83-C84-C85-C86-C87; Cg(5): C88-C89-C90-C91-C92-C93						

d[Cg(I)-Cg(J)]: Distance between ring centroids.

d[Cg(I)···⊥P(J)]: Perpendicular distance of Cg(I) on ring J.

d[⊥P(I)···Cg(J)]: Perpendicular distance of Cg(J) on ring I.

α: Dihedral angle between planes I and J.

β: Angle Cg(I)→Cg(J) vector and normal to plane I.

γ: Angle Cg(I)→Cg(J) vector and normal to plane J.

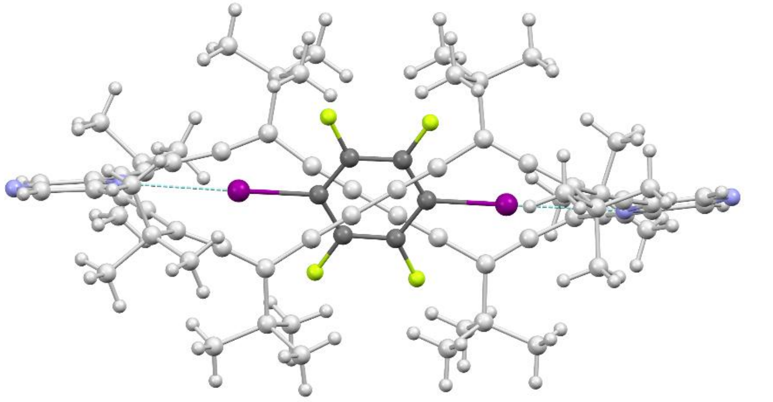

Figure S15b. *Complex 1,4-Diiodotetrafluorobenzene@(P$_4$)-**2***

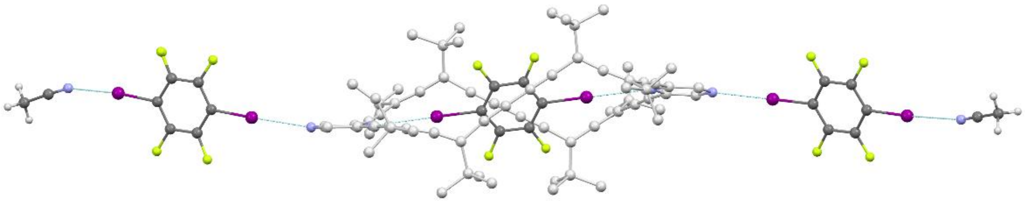

Figure S16. *Aggregate "1,4-Diiodotetrafluorobenzene@(P$_4$)-**2**" + 2 IC$_6$F$_4$I + 2 acetonitrile.*

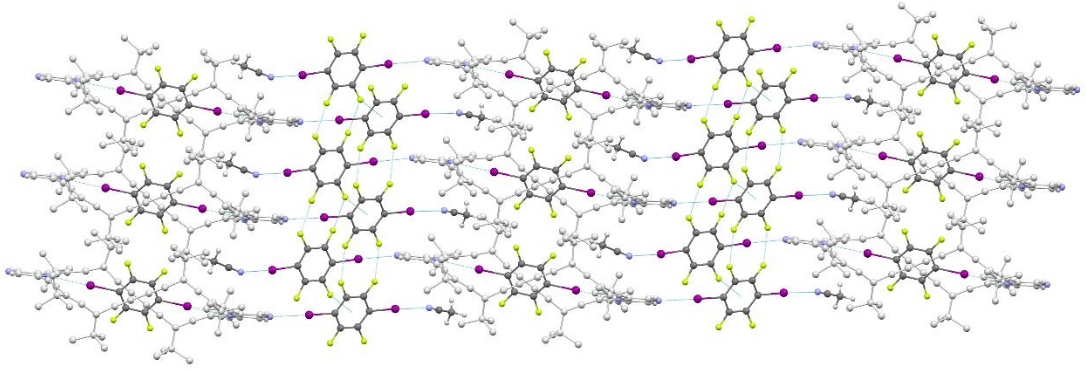

Figure S17. *Representation of the two-dimensional network.*

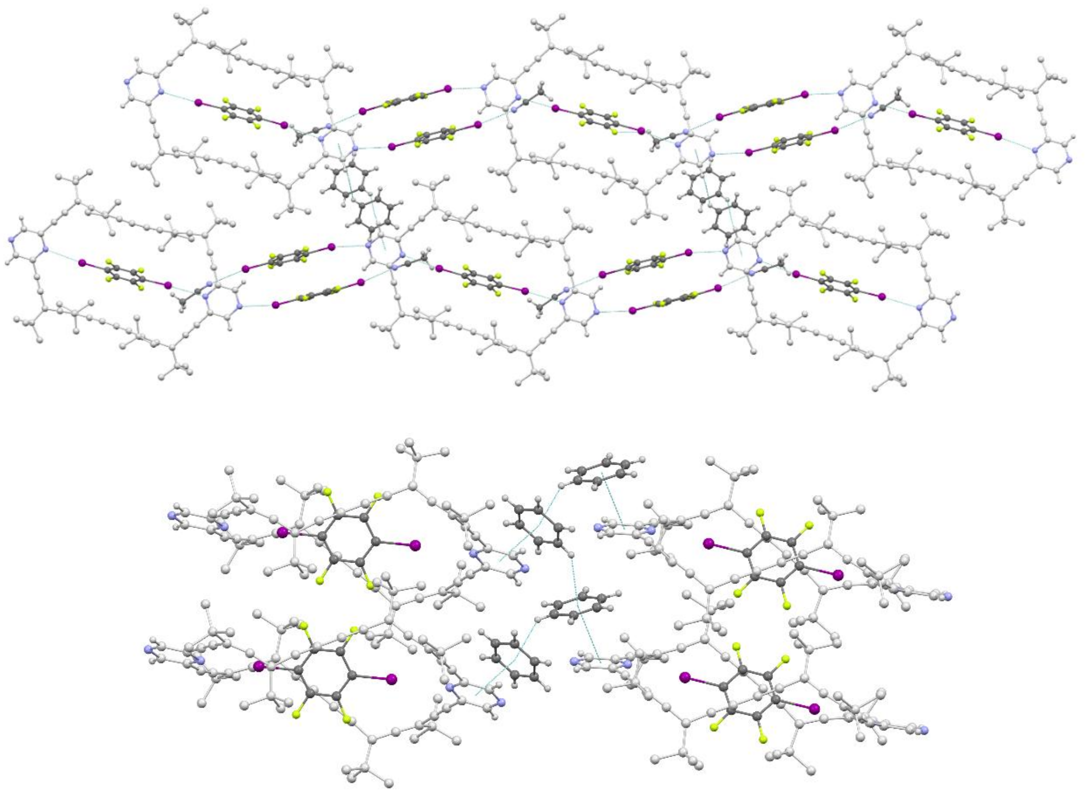

Figure S18. *Representation of the 3D organization and details of the C-H···π and π···π interactions.*

4. Computational details

The geometries and frequencies of the conformers of (*P_4*)-**2** (boat, chair, twist and twisted-boat) were computationally calculated using *Gaussian09*[11] and the CAM-B3LYP[12]method with the 6-31g+(d,p) basis set, including solvation effects using the smd model[13] with chloroform parameters. Geometries of the halogen bond complexes were optimized with the 6-31g+(d,p) basis sets using the LANL08(d) basis set on the iodine atom and solvation effects were taken into account using IEFPCM model[14] with benzene parameters. The structures were characterized as minima in the potential surface by analytical computation of vibrational frequencies, with no imaginary frequencies detected. This level of theory was previously validated as sufficient to explore the conformational space of allenophanes, their complexes and to reproduce their circular dichroism spectra with excellent accuracy and computational efficiency.[15,16] However, to verify that the inclusion of dispersion corrections does not induce significant changes in the optimized geometries, we also recalculated the geometry of one conformer of allenophane (*P_4*)-**2** and one of its host–guest complexes (**Figures S19b** and **S22b**) using B3LYP-D3/6-31G+(d,p), and found only minimal differences. Excited state energies and properties have been computed at TD-DFT CAM-B3LYP 6-31g+(d,p) level, including solvation effects using the SMD model with chloroform parameters. For circular dichroism spectra, the first 50 excited states were computed.

To compute the electrostatic potential maps of the selected guest molecules, three different computational approaches were employed in order to compare with previously reported methods. First, geometry optimizations were carried out using the ωB97X-D functional[17] in combination with the Def2TZVPP[18,19] basis set for all atoms, including pseudopotentials for the heavy iodine atoms, obtained from the Basis Set Exchange library.[20] As alternative strategies, the ωB97X-D functional was also used with the aug-cc-pVTZ[21] basis set for carbon and fluorine atoms, and the aug-cc-pVTZ-PP[18,22] basis set—with corresponding pseudopotentials—for iodine. Additionally, a third approach employing the B3LYP functional completed with D3 Grimme dispersion correction[23], with the same aug-cc-pVTZ/aug-cc-pVTZ-PP basis set combination, was tested. The results obtained from all three methods are summarized in **Table S5**. All computational protocols have been previously validated in the literature, providing reliable results for related systems. [24-27] The electrostatic potential maps were visualized using Gaussview, employing an isosurface level of 0.001 a.u.. The extrema values associated with the σ-hole and/or electronegative regions were calculated using the Multiwfn software.[28,29]

(P_4)-2 in the boat conformation

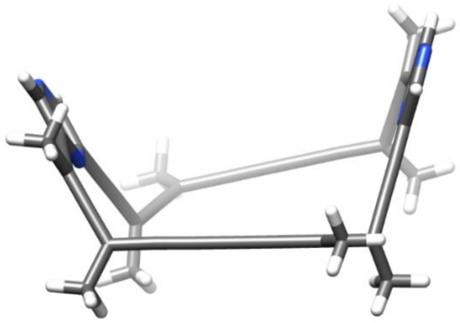

C	2.66722100	-5.34481900	-0.75316600
C	1.41236200	-5.87974900	-0.61882000
C	3.76683600	-4.84730700	-0.87495400
C	0.29415100	-6.33257800	-0.49251300
C	5.27693500	-0.75758700	-0.50045800
C	5.28639000	0.07812100	0.37318600
C	-3.18538700	-5.27568400	1.94514000
C	-3.84269400	-4.26985300	2.08174200
C	4.59668100	3.06442900	2.24290400
C	5.28592000	1.04963700	1.42493000
C	5.98826300	0.81244400	2.61477900
C	5.30583300	2.81934600	3.42755200
C	3.18533700	5.27558900	1.94474600
C	-0.29434400	6.33287600	-0.49263900
C	-1.41253700	5.87997500	-0.61884200
C	-2.66733200	5.34486300	-0.75306300
C	-3.76690100	4.84722600	-0.87475500
C	-5.27687900	0.75743600	-0.50034100
C	-5.28572700	-1.04968100	1.42514500
N	-4.59298100	-2.17862100	1.24114600
C	-5.98799500	-0.81231400	2.61500400
C	-4.59661900	-3.06445200	2.24328500
H	6.54543400	-0.10858800	2.75305900
C	-5.07297100	4.27436400	-1.01090100

C	-5.24586200	1.71211700	-1.57076200
C	-2.39744700	-6.46681600	1.81947800
C	-1.03373800	-6.86032500	-0.39305100
C	5.07291800	-4.27447700	-1.01112300
C	5.24579300	-1.71221600	-1.57092300
C	2.39743900	6.46675800	1.81917700
C	1.03363400	6.86041600	-0.39326700
C	-5.17568000	2.99390200	-1.29105200
C	-1.71754800	-6.67146100	0.71334600
C	5.17565400	-2.99401500	-1.29126300
C	1.71747900	6.67147400	0.71309600
N	4.59306400	2.17849700	1.24085500
C	-5.30569700	-2.81919400	3.42794100
H	-5.30358100	-3.54684100	4.23339200
H	5.30368200	3.54706600	4.23293700
H	-6.54508800	0.10877600	2.75321900
C	3.84261700	4.26973400	2.08129200
C	-5.28618100	-0.07828200	0.37329400
C	-2.38271100	-7.42820100	2.98856200
C	-1.58804600	-7.60294800	-1.59049400
C	-5.27648300	1.17625200	-2.98627300
C	6.26922100	-5.18243800	-0.82223600
C	5.27624400	-1.17628600	-2.98641200
C	1.58792100	7.60308200	-1.59069300
C	2.38291200	7.42815700	2.98825300
C	-6.26929500	5.18226300	-0.82184100
H	-0.96146100	-8.46984800	-1.82031300
H	-2.60650500	-7.94222600	-1.39504100
H	-1.59369300	-6.94906000	-2.46749600
H	-2.00704600	-6.92535000	3.88468500
H	-3.39649500	-7.78108100	3.20022700
H	-1.74640200	-8.28767200	2.77226700
H	-5.23146700	1.99311100	-3.70821400
H	-4.42646200	0.50772400	-3.15138100
H	-6.19287600	0.60251800	-3.15360000

H	-7.19882900	4.62492400	-0.94849200
H	-6.24205300	5.99895500	-1.54932200
H	-6.25277000	5.62297000	0.17935800
H	1.59345200	6.94925800	-2.46774300
H	2.60642200	7.94226000	-1.39528500
H	0.96139200	8.47005100	-1.82040500
H	7.19876600	-4.62513100	-0.94894300
H	6.24187300	-5.99908400	-1.54976400
H	6.25277400	-5.62320700	0.17893800
H	4.42619900	-0.50775600	-3.15139200
H	6.19261300	-0.60253800	-3.15381800
H	5.23114900	-1.99311200	-3.70838700
H	1.74665400	8.28768000	2.77201600
H	2.00730200	6.92534700	3.88442100
H	3.39675000	7.78095200	3.19980400
N	5.99647200	1.69831700	3.60557100
N	-5.99623200	-1.69808900	3.60588200

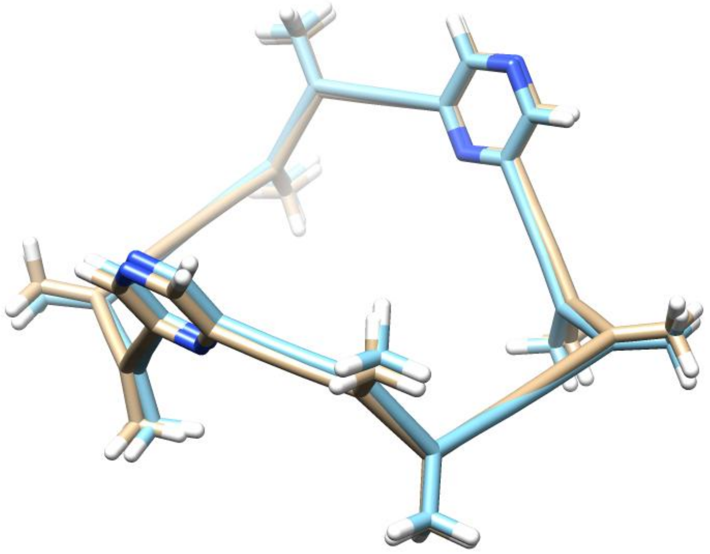

Figure S19b. *Comparison of the calculated geometries of (P_4)-**2** in its boat conformation. The gold structure corresponds to the DFT-optimized geometry at the CAM-B3LYP/6-31G+(d,p) level with SMD solvation (chloroform), and the cyan structure to the DFT-optimized geometry at the B3LYP-D3/6-31G+(d,p) level with SMD solvation (chloroform).*

(P_4)-2 in the twist conformation

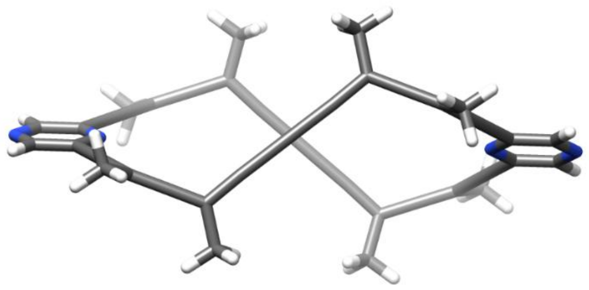

C	-0.43248700	4.36675100	-0.53115900
C	0.43274300	4.36681800	0.53140300
C	-1.19272000	4.35696300	-1.47633000
C	1.19296700	4.35713300	1.47658200
C	-0.94551200	3.28475500	-5.73990000
C	-0.65243600	2.28493400	-6.35376800
C	0.94559100	3.28498600	5.74007800
C	0.65239500	2.28512500	6.35382400
C	0.31130300	-1.09726300	-7.07627800
C	-0.31190800	1.09715600	-7.07614000
C	-0.30914100	1.09222000	-8.47832200
C	0.30818800	-1.09225000	-8.47846000
C	0.94515200	-3.28491200	-5.74023800
C	1.19278800	-4.35717000	-1.47659400
C	0.43272500	-4.36707800	-0.53128900
C	-0.43256900	-4.36712900	0.53122200
C	-1.19280300	-4.35731500	1.47639100
C	-0.94520000	-3.28474200	5.73990700
C	-0.31149100	-1.09709800	7.07602500
N	0.00013400	0.00012500	6.37765600
C	-0.30854400	-1.09216300	8.47820600
C	0.31176800	1.09730700	7.07608400
H	-0.56295700	1.98928500	-9.03420500
C	-2.13306100	-4.32400600	2.55534700
C	-1.28109200	-4.48863200	5.03663000
C	1.28174800	4.48879600	5.03679200
C	2.13338500	4.32402400	2.55540400

C	-2.13312200	4.32376000	-2.55516300
C	-1.28149100	4.48857700	-5.03655000
C	1.28109200	-4.48871000	-5.03682700
C	2.13303400	-4.32375500	-2.55555700
C	-1.70376000	-4.41030600	3.79470300
C	1.70425100	4.41038000	3.79481400
C	-1.70398200	4.41015100	-3.79456900
C	1.70374300	-4.41023300	-3.79490400
N	-0.00021200	-0.00007100	-6.37781200
C	0.30883100	1.09229400	8.47826500
H	0.56259300	1.98932300	9.03422900
H	0.56187200	-1.98928300	-9.03445500
H	-0.56230300	-1.98922400	9.03412100
C	0.65202100	-2.28507900	-6.35406000
C	-0.65212900	-2.28487400	6.35369900
C	1.12991400	5.80093400	5.77637500
C	3.59875300	4.17805800	2.20109800
C	-1.12886200	-5.80074300	5.77618100
C	-3.59847400	4.17760300	-2.20087500
C	-1.12945100	5.80073700	-5.77605300
C	3.59842300	-4.17748000	-2.20146300
C	1.12877700	-5.80091900	-5.77618700
C	-3.59848500	-4.17815600	2.20122800
H	-1.75761900	-5.80512500	6.67166500
H	-1.41560800	-6.63780000	5.13778500
H	-0.09049400	-5.93621900	6.09314600
H	-4.21220200	-4.16143400	3.10319400
H	-3.91811200	-5.01075500	1.56761500
H	-3.75876900	-3.25007200	1.64481900
H	1.75751100	-5.80545700	-6.67168700
H	0.09039400	-5.93639100	-6.09310800
H	1.41549800	-6.63789900	-5.13768100
H	-4.21230200	4.16087200	-3.10276600
H	3.91833400	5.01056400	1.56733900
H	4.21259500	4.16141300	3.10298100

H	1.41679200	6.63792900	5.13795700
H	1.75876800	5.80515600	6.67179200
H	3.75846400	-3.24930700	-1.64513300
H	0.09161300	5.93665600	6.09345300
H	3.91827600	-5.00994100	-1.56778300
H	4.21212700	-4.16067000	-3.10343600
H	-3.75851900	3.24942700	-1.64455200
H	-3.91817100	5.01007200	-1.56712700
H	-1.41619000	6.63773900	-5.13758300
H	-0.09113100	5.93631300	-6.09313300
H	-1.75831200	5.80511300	-6.67146400
H	3.75891000	3.24989800	1.64477900
N	0.00014600	0.00004600	9.16935100
N	-0.00056200	0.00000400	-9.16950600

(*P*$_4$)-2 in the chair conformation

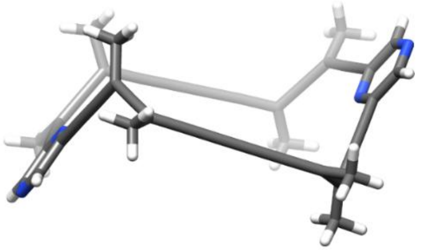

C	-0.66400900	0.18208600	6.33363600
C	0.64936300	-0.20908200	6.33481600
C	-1.82560300	0.53037000	6.30073100
C	1.81108600	-0.55712900	6.30399200
C	-4.73684500	0.83038700	3.05811400
C	-5.19735300	0.06145300	2.24662600
C	4.73121800	-0.84357400	3.06831300
C	5.19364900	-0.07129100	2.26111000
C	-5.92596400	-1.58048100	-0.86170700
C	-5.72748500	-0.86969900	1.29676400
C	-6.52835100	-1.94048000	1.71780000
C	-6.72954200	-2.64575900	-0.43069400
C	-5.31122200	-1.29867600	-3.41371300

C	-1.85972300	0.36315200	-5.55295100
C	-0.66703900	0.14210900	-5.56558600
C	0.67897200	-0.11605600	-5.56471300
C	1.87160000	-0.33730000	-5.55039100
C	5.31841300	1.31261000	-3.39367200
C	5.92820400	1.58297900	-0.83925000
N	5.43050600	0.69010600	0.02328700
C	6.73127900	2.64633100	-0.40255900
C	5.72574400	0.86359100	1.31600200
H	-6.76148000	-2.07555900	2.76913400
C	3.26982500	-0.64617400	-5.53426400
C	4.98254400	1.20119400	-4.78305300
C	4.16767900	-1.79474500	3.98278000
C	3.18599300	-0.95711100	6.27263200
C	-3.20034100	0.93073100	6.26699800
C	-4.17556700	1.77780500	3.97784900
C	-4.97214800	-1.18015500	-4.80172500
C	-3.25807300	0.67156100	-5.53901000
C	4.12946100	0.27665400	-5.16403400
C	3.69331900	-1.37975000	5.13549400
C	-3.70436500	1.35809100	5.13015600
C	-4.11842300	-0.25339700	-5.17582100
N	-5.42999900	-0.69095800	0.00529600
C	6.52604900	1.93251800	1.74272600
H	6.75733500	2.06339400	2.79500000
H	-7.12878000	-3.35985000	-1.14392200
H	7.13195700	3.36313500	-1.11225400
C	-5.60422000	-1.42538000	-2.24727700
C	5.60897800	1.43373400	-2.22603500
C	4.13196200	-3.24442100	3.54813300
C	3.98256300	-0.86676500	7.55630100
C	5.63125500	2.17444200	-5.74404700
C	-4.00061900	0.83532200	7.54799600
C	-4.13860300	3.22921500	3.54915600
C	-3.67233000	2.07162700	-5.94073500

C	-5.61821700	-2.14862000	-5.76928600
C	3.68482300	-2.04414400	-5.94246600
H	-3.54539800	1.45712700	8.32456500
H	-5.02749400	1.16643800	7.38512000
H	-4.01307700	-0.19751300	7.90850600
H	5.00986600	-1.19737600	7.39512200
H	3.99408000	0.16465600	7.92087400
H	3.52502400	-1.49154900	8.32910900
H	6.72008000	2.07425900	-5.70566800
H	5.29443900	1.99010800	-6.76531700
H	5.37756800	3.20212300	-5.46729800
H	5.14678700	-3.60823000	3.36181800
H	3.66707600	-3.86489900	4.31580000
H	3.56354200	-3.34233200	2.61860700
H	-3.56805100	3.33078500	2.62133300
H	-5.15293900	3.59395900	3.36199800
H	-3.67536200	3.84649900	4.32038100
H	-5.36497300	-3.17763900	-5.49714100
H	-6.70716000	-2.04889300	-5.73315000
H	-5.27887100	-1.95898300	-6.78875200
H	-3.34701000	2.28246100	-6.96374400
H	-4.75603700	2.18405400	-5.88169800
H	-3.20272200	2.80562100	-5.27936600
H	4.76832300	-2.15723400	-5.88094700
H	3.21308800	-2.78161700	-5.28650400
H	3.36237600	-2.24923200	-6.96755300
N	7.02531200	2.81395100	0.88223700
N	-7.02585500	-2.81857400	0.85290200

(P_4)-2 in the twisted-boat conformation

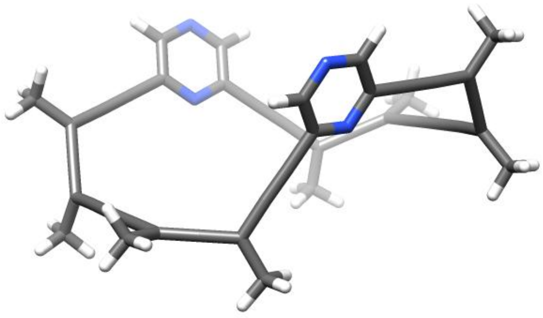

C	2.95721500	-4.62816200	1.00852400
C	3.03120300	-5.33989100	2.21403900
H	2.38663100	-6.19481800	2.39113000
C	4.66569500	-3.94527700	2.94704600
H	5.36367400	-3.65514700	3.72583600
C	4.59721800	-3.22457900	1.74603000
C	5.43636300	-2.08544700	1.53322400
C	6.13001300	-1.11055400	1.35752300
C	6.95445500	0.04957000	1.18520300
C	6.83217500	0.76657300	0.09044100
C	6.69660000	1.48304600	-1.00296100
C	5.73698900	2.54617800	-1.02847800
C	4.92290600	3.44319400	-1.09186000
C	3.98765400	4.44325100	-1.15591600
C	3.14360200	5.31235300	-1.21673300
C	2.16215600	6.35393800	-1.28221200
C	0.92258300	6.03926100	-1.58713700
C	-0.30738100	5.69751200	-1.89727100
C	-1.22593800	5.33846100	-0.85513600
C	-2.02687500	5.01414300	-0.00950500
C	-2.95722100	4.62813900	1.00852100
C	-3.03113700	5.33983500	2.21406200
H	-2.38650000	6.19471000	2.39116300
C	-4.66569200	3.94529600	2.94706500
H	-5.36368500	3.65519200	3.72585200
C	-4.59727500	3.22462300	1.74602900

C	-5.43647400	2.08552700	1.53323300
C	-6.13012700	1.11063000	1.35756700
C	-6.95443100	-0.04958900	1.18524300
C	-6.83212600	-0.76655000	0.09045600
C	-6.69649800	-1.48296400	-1.00297800
C	-5.73694600	-2.54615300	-1.02846800
C	-4.92293900	-3.44323900	-1.09182800
C	-3.98770600	-4.44331400	-1.15590100
C	-3.14367800	-5.31244000	-1.21669100
C	-2.16220300	-6.35399400	-1.28221500
C	-0.92262700	-6.03928600	-1.58708900
C	0.30732000	-5.69748900	-1.89723700
C	1.22588500	-5.33844400	-0.85510400
C	2.02683700	-5.01415000	-0.00947800
N	3.74397200	-3.57198400	0.77639300
N	3.88512600	-4.99636100	3.17297500
N	-3.74403600	3.57200800	0.77638200
N	-3.88505800	4.99633000	3.17300700
C	-0.81349100	5.64408600	-3.32316700
H	-1.15669600	4.63281000	-3.56047300
H	-1.66031000	6.32514600	-3.44957000
H	-0.02396800	5.92431800	-4.02212400
C	-7.92522200	-0.39426900	2.29441500
H	-7.38387000	-0.55739700	3.23108100
H	-8.62848300	0.42920900	2.45067900
H	-8.48622300	-1.29722300	2.04864800
C	-7.50387200	-1.23303300	-2.25935000
H	-6.83619000	-1.00065000	-3.09416400
H	-8.07631200	-2.12696700	-2.52413000
H	-8.19293500	-0.39958400	-2.11427100
C	0.81338300	-5.64391500	-3.32314000
H	1.66024900	-6.32490200	-3.44962400
H	0.02386200	-5.92414500	-4.02210000
H	1.15650600	-4.63259600	-3.56038300
C	7.50409000	1.23321000	-2.25928200

H	6.83649400	1.00065300	-3.09411800
H	8.07637300	2.12723800	-2.52407500
H	8.19329500	0.39989200	-2.11412600
C	7.92541100	0.39403400	2.29429000
H	7.38419800	0.55704200	3.23105700
H	8.62865600	-0.42950200	2.45033400
H	8.48642600	1.29699600	2.04858400
C	2.61208600	7.77031700	-0.99442500
H	3.02720100	7.83530800	0.01572100
H	3.39390200	8.06871500	-1.69910200
H	1.77432300	8.46441000	-1.07850200
C	-2.61215000	-7.77041700	-0.99465900
H	-3.02730200	-7.83555500	0.01546100
H	-3.39394100	-8.06870600	-1.69941000
H	-1.77438700	-8.46450200	-1.07880700

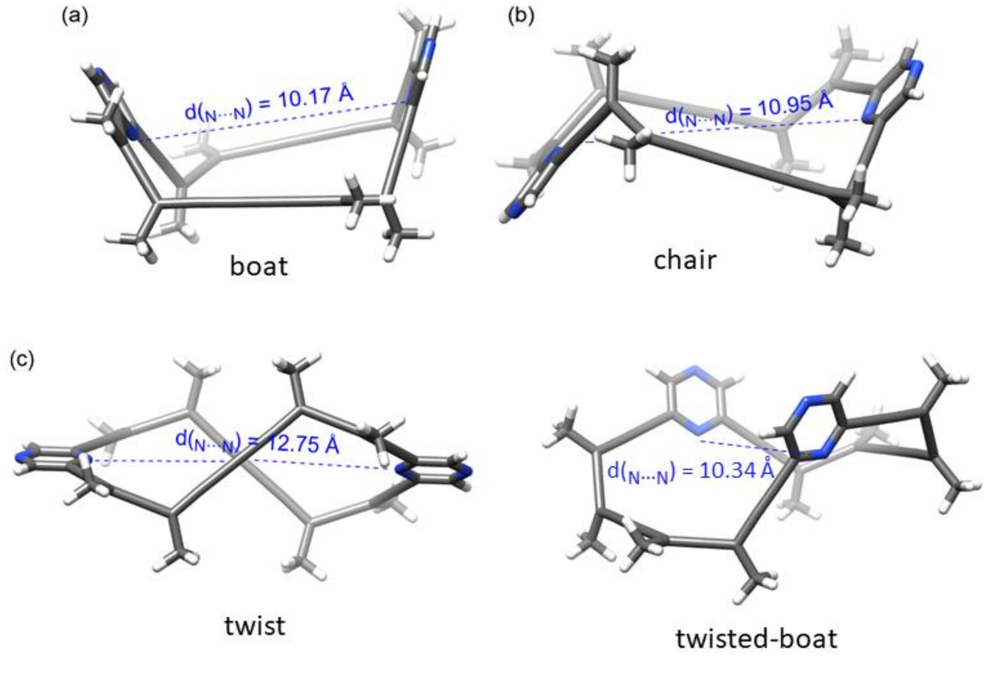

Figure S19b. *Calculated geometries for (P_4)-**2** at the DFT CAM-B3LYP/6-31g+(d,p) smd = chloroform level of theory: (a) boat, (b) chair, (c) twist and twisted-boat (d) conformation. Tert-butyl groups were replaced by methyl groups.*

(P_4)-2 and guest complexes

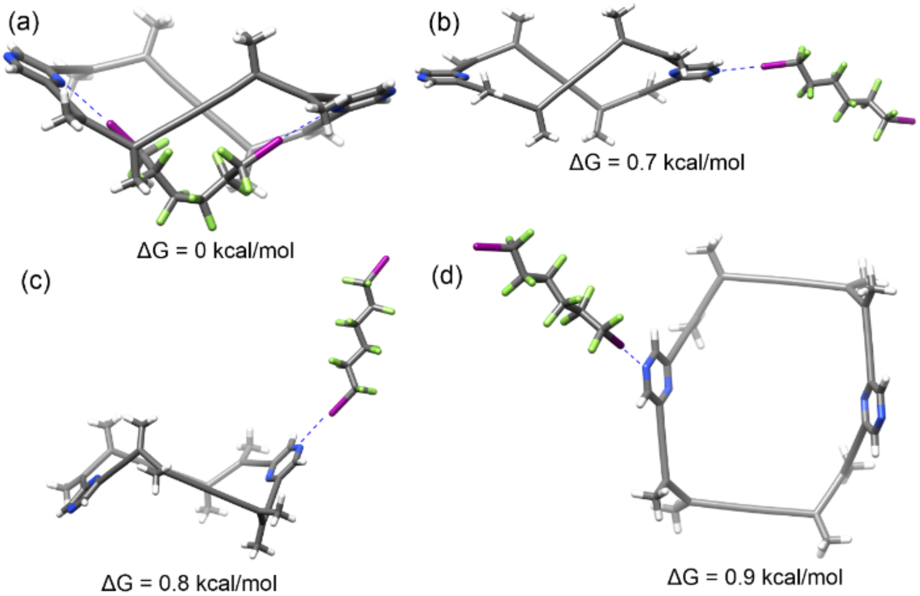

Figure S20. *Geometries of the* ***G1****@(P*$_4$*)-****2*** *halogen bond complex at the DFT CAM-B3LYP/6-31G* level using LANL08(d) basis set on the iodine atoms and their energy difference with respect to the most stable conformer (a). Solvation effects were taken into account using IEFPCM model with benzene parameters. Halogen bond interactions are represented as dashed blue lines.*

G1@(P_4)-**2** (a)

C	-0.89068700	4.55000900	-0.73755300
C	-0.10262700	4.42341500	0.37437100
C	-1.59406400	4.66904400	-1.71628700
C	0.57251700	4.32128300	1.37454600
C	-1.61524800	3.43305600	-5.94848700
C	-1.56369900	2.35516000	-6.48968100
C	-0.11798700	3.19212800	5.60586200
C	-0.52025100	2.22938000	6.21433300
C	-0.95838500	-1.15531400	-6.94666900
C	-1.51223000	1.05842600	-7.08745700

C	-2.01926700	0.83949400	-8.37493800
C	-1.47444900	-1.35090300	-8.23686800
C	0.03255900	-3.19645200	-5.60663900
C	0.76085800	-4.29190500	-1.37323600
C	0.08859400	-4.42437200	-0.37468200
C	-0.69536300	-4.58591900	0.73560700
C	-1.39445900	-4.73570700	1.71318500
C	-1.47168600	-3.50442400	5.94575300
C	-1.47042200	-1.12821900	7.08611100
N	-0.98060500	-0.10324600	6.37629600
C	-1.98163900	-0.93305600	8.37576700
C	-1.01524100	1.10787900	6.94560200
H	-2.44954400	1.66024200	-8.94091500
C	-2.29378400	-4.91747600	2.80884900
C	-1.46010200	-4.78084900	5.29498200
C	0.33571500	4.36677100	4.92545100
C	1.40535300	4.21290500	2.53150800
C	-2.49928000	4.81141400	-2.81292800
C	-1.65884100	4.70917700	-5.29843200
C	0.53829500	-4.34893800	-4.92504900
C	1.59150000	-4.14521800	-2.52754900
C	-1.86335300	-4.85951300	4.04796800
C	0.86891700	4.27645500	3.72830600
C	-2.06584400	4.77116200	-4.05170100
C	1.06267900	-4.23397300	-3.72612500

N	-0.97493500	0.05681100	-6.37865900
C	-1.53456700	1.27934800	8.23793800
H	-1.55624000	2.26607700	8.69090600
H	-1.45440900	-2.33812000	-8.68884300
H	-2.37296600	-1.77254400	8.94252500
C	-0.41246100	-2.25312300	-6.21574200
C	-1.46649100	-2.42549900	6.48733700
C	0.16774600	5.69231100	5.64210800
C	2.89550000	4.04463200	2.31529400
C	-0.97487400	-5.97740900	6.08621300
C	-3.96617300	5.00285200	-2.47939400
C	-1.22521200	5.92507800	-6.09014200
C	3.07144300	-3.90755700	-2.30639200
C	0.43426100	-5.68072100	-5.64221200
C	-3.75085600	-5.17125400	2.47399000
H	-1.59098000	-6.12073600	6.97943500
H	-1.01769400	-6.88266800	5.47825000
H	0.05723600	-5.82173500	6.41472600
H	-4.34215400	-5.28990200	3.38373400
H	-3.84822900	-6.07531600	1.86532500
H	-4.15379600	-4.33490500	1.89521100
H	0.98065200	-5.64824000	-6.59000200
H	-0.61164900	-5.91050600	-5.86792500
H	0.84488900	-6.48201300	-5.02561100
H	-4.56116400	5.09612700	-3.38968300

H	3.29284800	4.90236700	1.76400800
H	3.41623300	3.96266300	3.27097800
H	0.54311000	6.51174200	5.02686800
H	0.71174600	5.68499400	6.59179600
H	3.22380900	-3.00133500	-1.71852700
H	-0.88844100	5.87356000	5.86412500
H	3.50641500	-4.74563400	-1.75326900
H	3.59105500	-3.80184400	-3.26034800
H	-4.33363700	4.15014200	-1.90087500
H	-4.10247000	5.90197600	-1.87088900
H	-1.30610900	6.82781800	-5.48230800
H	-0.18761600	5.81329200	-6.41920600
H	-1.84734200	6.04199400	-6.98304200
H	3.09189300	3.14683100	1.72759900
N	-2.01003000	0.26485100	8.94779300
N	-1.99687400	-0.35910400	-8.94579300
C	2.71284800	0.13461300	-1.68183800
C	3.80451800	0.53058500	-0.63226700
C	3.82317100	-0.35394800	0.63821500
C	2.03582100	1.34031600	-2.35661300
F	3.31587900	-0.61780900	-2.62127000
F	1.76175100	-0.61652600	-1.09234200
F	5.00699400	0.40799100	-1.21655500
F	3.66559400	1.81678800	-0.24320600
F	3.74694300	-1.64525100	0.24869500

F	5.01618200	-0.17438300	1.22712700
F	3.27284900	0.76928600	2.62560900
C	2.70989700	-0.01038600	1.68351500
F	1.72670000	0.69507300	1.09038100
I	0.72468000	0.76263400	-3.95921800
I	0.74752400	-0.73186600	3.95506200
F	1.35127000	2.01290900	-1.41759100
F	3.01763000	2.13823200	-2.82669600
F	1.43882400	-1.95037800	1.41427400
C	2.08825000	-1.24701300	2.35566100
F	3.10491200	-1.99836100	2.82838600

G1@(*P_4*)-**2** (b)

C	-0.49242900	4.51831000	-0.75947400
C	0.32751300	4.52249700	0.33624700
C	-1.20931200	4.50549900	-1.73551700
C	1.04475500	4.51734500	1.31205100
C	-0.85953500	3.32071900	-5.97664000
C	-0.58577400	2.30204600	-6.56390800
C	0.70216500	3.35992800	5.55801100
C	0.43148300	2.34247700	6.14895500
C	0.30245400	-1.10259800	-7.26515000
C	-0.27309000	1.09912800	-7.27283500
C	-0.27939100	1.08431300	-8.67504200
C	0.28870500	-1.10276400	-8.66741200
C	0.90855300	-3.30998300	-5.95394100

C	1.22369100	-4.47831000	-1.70555100
C	0.49350000	-4.50304400	-0.73965800
C	-0.34223300	-4.52197100	0.34390100
C	-1.07430400	-4.53044900	1.30860500
C	-0.85294500	-3.32479400	5.55369200
C	-0.38519300	-1.08102700	6.85702900
N	-0.09920100	0.02372500	6.16071700
C	-0.44964900	-1.06313800	8.25741600
C	0.11999000	1.14220500	6.85958800
H	-0.51834200	1.98517900	-9.23296100
C	-1.98979700	-4.52738200	2.40607900
C	-1.10522600	-4.56235300	4.87844100
C	1.01202600	4.58245000	4.87970300
C	1.94116000	4.49437900	2.42498000
C	-2.10479000	4.47425600	-2.84919700
C	-1.17237500	4.54542600	-5.30314300
C	1.23356500	-4.52713800	-5.27271000
C	2.13324600	-4.43338200	-2.80729400
C	-1.54229000	-4.54859000	3.64028600
C	1.47290600	4.54289300	3.65066400
C	-1.63472400	4.51403700	-4.07473900
C	1.67993000	-4.48430800	-4.03871600
N	0.01965500	0.00202100	-6.56718500
C	0.05036000	1.15176600	8.25992800
H	0.22805800	2.06415200	8.82039900

H	0.51972700	-2.00956600	-9.21902400
H	-0.68230000	-1.96406800	8.81641200
C	0.62580900	-2.29772500	-6.54802900
C	-0.63168800	-2.29522200	6.14415600
C	0.79091400	5.87938100	5.63115000
C	3.42475000	4.40500100	2.12695000
C	-0.85519400	-5.84493800	5.64536900
C	-3.58846400	4.38637000	-2.55088700
C	-0.95251500	5.83941200	-6.06002500
C	3.61106800	-4.31881700	-2.48975700
C	1.04525200	-5.82695900	-6.02810400
C	-3.47016500	-4.49203700	2.08158000
H	-1.47704100	-5.87926800	6.54536600
H	-1.08217100	-6.71413800	5.02576500
H	0.19102300	-5.90058000	5.96122300
H	-4.06622000	-4.48826200	2.99586200
H	-3.74723100	-5.36229600	1.47897000
H	-3.70602500	-3.59557300	1.50057700
H	1.65840700	-5.83342300	-6.93468200
H	0.00028400	-5.94030300	-6.33251000
H	1.32501400	-6.67886900	-5.40596200
H	-4.16710200	4.36351700	-3.47601400
H	3.74221000	5.25945200	1.52165700
H	4.00439000	4.38931000	3.05159600
H	1.06142300	6.73487700	5.00995500

H	1.39427600	5.89960000	6.54405700
H	3.80269100	-3.40844700	-1.91404600
H	-0.25920700	5.97427900	5.92387800
H	3.93716500	-5.17048900	-1.88501400
H	4.20136600	-4.28767600	-3.40724100
H	-3.80365800	3.48084400	-1.97586100
H	-3.90746700	5.24503300	-1.95227400
H	-1.22530100	6.69797400	-5.44395600
H	0.09789800	5.93388300	-6.35186000
H	-1.55428100	5.85341400	-6.97412000
H	3.63937400	3.49544900	1.55820100
N	-0.23277900	0.05102400	8.94708400
N	-0.00033500	-0.01297100	-9.36819400
C	0.64212800	0.10372000	14.77473500
C	0.46929300	0.29094000	16.30327800
C	1.77735600	0.02855900	17.09045900
C	-0.68928900	-0.07042600	14.01830900
F	1.28175400	1.18958900	14.30663700
F	1.39968100	-0.98855000	14.55551200
F	0.07006700	1.55563400	16.53141300
F	-0.46643200	-0.55770900	16.76245800
F	1.95351300	-1.30132400	17.18036300
F	2.81279100	0.56814900	16.42276300
F	1.95877900	1.96290600	18.39455000
C	1.75445100	0.64189100	18.51458500

F	0.54987200	0.42111700	19.07051000
I	-0.44925500	0.01104600	11.87594100
I	2.98857600	1.14130900	21.30044100
F	-1.21626600	-1.25650700	14.36601800
F	-1.52739200	0.90119300	14.42944600
F	2.51260000	-1.22404600	19.71873600
C	2.83045700	0.04475300	19.44702700
F	4.00868500	0.06880600	18.81020200

G1@(P_4)-**2** (c)

C	0.18313900	0.21353400	6.14091800
C	1.33848500	-0.51760700	6.07790700
C	-0.83951100	0.86199600	6.16654500
C	2.35800700	-1.16581400	5.99260300
C	-3.78854200	1.95312900	3.11079500
C	-4.49980200	1.35995400	2.33662000
C	4.81190800	-2.43344900	2.60189100
C	5.38195500	-1.87487100	1.69620900
C	-5.89040400	-0.03342200	-0.66272700
C	-5.34531800	0.62321900	1.44695600
C	-6.40966800	-0.13766300	1.95094500
C	-6.95351500	-0.78680400	-0.14443300
C	-5.40960800	-0.03437600	-3.25619500
C	-1.78375900	0.34732300	-5.70962100
C	-0.72741600	-0.24029600	-5.78502500
C	0.46347200	-0.91202600	-5.84948900

C	1.52319000	-1.49695700	-5.88967200
C	5.45894400	-0.90450000	-4.05560700
C	6.28110200	-0.66630000	-1.56235000
N	5.64205200	-1.35200800	-0.60883300
C	7.34495800	0.19266900	-1.25256900
C	6.05585100	-1.17177800	0.64930000
H	-6.60371500	-0.17112200	3.01908700
C	2.75029800	-2.22995600	-5.92435100
C	5.00845000	-0.99086500	-5.41190200
C	4.10302100	-3.13374000	3.63111200
C	3.56941000	-1.91980600	5.89766000
C	-2.05096000	1.62100200	6.20681500
C	-2.92177800	2.69736600	3.97553700
C	-5.15776600	-0.07367800	-4.66517700
C	-3.00399300	1.08725400	-5.61940000
C	3.88361600	-1.61465600	-5.67757100
C	3.85259000	-2.53543400	4.77240600
C	-2.50155900	2.16612400	5.09967200
C	-4.08499300	0.50867000	-5.14926700
N	-5.08790600	0.67736900	0.13641400
C	7.11742100	-0.30739300	0.95094600
H	7.44529700	-0.16446900	1.97567800
H	-7.59805700	-1.35636400	-0.80787300
H	7.86026100	0.74499100	-2.03209300
C	-5.63303600	-0.02176900	-2.06976400

C	5.84386100	-0.81013000	-2.91512200
C	3.64887900	-4.54707700	3.32819100
C	4.47573400	-1.96676600	7.11036200
C	5.85738800	-0.33546200	-6.48165100
C	-2.76049000	1.75698400	7.53827700
C	-2.50946800	4.08338100	3.52290900
C	-2.98298700	2.53471700	-6.06797000
C	-6.15478600	-0.80677100	-5.53899400
C	2.67802500	-3.71099100	-6.23667400
H	-2.10455100	2.23555400	8.27191200
H	-3.66789800	2.35418600	7.43216500
H	-3.02943700	0.77009900	7.92696300
H	5.36333700	-2.56827100	6.90625300
H	4.78883600	-0.95576000	7.38790400
H	3.94446000	-2.39654700	7.96500000
H	6.86035800	-0.77316800	-6.49492100
H	5.40321500	-0.46350300	-7.46565500
H	5.96367200	0.73464400	-6.27895900
H	4.50942100	-5.18505500	3.10471800
H	3.10673700	-4.96718200	4.17704800
H	2.99441000	-4.55197600	2.45147300
H	-1.99847400	4.02617300	2.55720400
H	-3.39008500	4.72043100	3.39572700
H	-1.83997900	4.54411200	4.25133200
H	-6.23228400	-1.85345700	-5.22919800

H	-7.14803200	-0.35715500	-5.44379300
H	-5.84970100	-0.77141200	-6.58624900
H	-2.69022400	2.60283200	-7.12014800
H	-3.96620000	2.99157600	-5.94246800
H	-2.25262700	3.10002200	-5.48145100
H	3.67381500	-4.15758500	-6.22435200
H	2.05197200	-4.22065300	-5.49828600
H	2.22853800	-3.86989200	-7.22156800
N	7.75470700	0.36507500	-0.00108100
N	-7.21146400	-0.83709600	1.15667900
C	13.39496500	8.33623600	0.10973200
C	12.15381800	7.46560600	0.43574300
C	12.50774600	5.97250100	0.64750400
C	13.14683500	9.84508400	0.32474600
F	14.41604000	7.96439400	0.90223100
F	13.72111700	8.12011500	-1.17419500
F	11.57087800	7.92238400	1.55787400
F	11.28940300	7.57618000	-0.58868900
F	13.41814700	5.60663300	-0.27346900
F	13.04354400	5.84539200	1.87357000
F	10.22815300	5.61679600	1.12977200
C	11.27762000	5.03977700	0.51306800
F	11.00914100	4.91536000	-0.79850900
I	14.76184000	11.04545200	-0.45819100
I	9.90008400	2.26846300	0.63549300

F	12.00227900	10.18223700	-0.28421400
F	13.02468200	10.08083700	1.63401400
F	12.66881400	3.16914300	0.67130500
C	11.49086100	3.64126900	1.12427700
F	11.57570700	3.77551300	2.45857500

G1@(P_4)-**2** (d)

C	3.09012900	-4.56982400	-0.87430100
C	1.98776900	-5.27359300	-0.46974600
C	4.05074400	-3.92696000	-1.23574800
C	1.00235700	-5.87622700	-0.10575900
C	4.81907500	0.36185500	-1.72579800
C	4.77893900	1.33734500	-1.01589400
C	-2.27914800	-5.03765800	2.68379500
C	-3.09494700	-4.15322000	2.78337300
C	3.80308200	4.44204300	0.50039600
C	4.74221700	2.46504200	-0.13736600
C	5.65655100	2.56255500	0.92069000
C	4.72333500	4.53352500	1.55417100
C	1.96376900	6.30437000	0.18742800
C	-2.01238200	6.37656500	-1.65990300
C	-3.03208900	5.73928700	-1.51606900
C	-4.17123100	4.99993500	-1.34319900
C	-5.16505500	4.32473900	-1.19080300
C	-5.85305000	0.13944200	-0.06504200
C	-5.22067800	-1.37147700	2.00317400

N	-4.35162400	-2.37261800	1.83262500
C	-5.78235400	-1.09804600	3.25836600
C	-4.04222600	-3.08995700	2.91777800
H	6.40346300	1.79213500	1.08385900
C	-6.34820000	3.54284700	-1.00273700
C	-6.17204900	0.93155100	-1.21542500
C	-1.29885700	-6.07735100	2.59464800
C	-0.17563800	-6.59129800	0.27718000
C	5.19618600	-3.17936700	-1.65433000
C	4.83160900	-0.76236900	-2.61364500
C	0.95391600	7.31105200	0.05993300
C	-0.80823800	7.11680000	-1.87755800
C	-6.27644600	2.23531300	-1.10603500
C	-0.73779200	-6.34314300	1.43756400
C	5.03085100	-1.96898300	-2.13661800
C	0.07326900	7.22282100	-0.91043900
N	3.81914700	3.40845600	-0.34776500
C	-4.61450300	-2.80663600	4.16628400
H	-4.35448200	-3.40113100	5.03745500
H	4.70728700	5.37777000	2.23625100
H	-6.48647300	-0.27986700	3.37876100
C	2.81276300	5.45635200	0.32104400
C	-5.56439400	-0.56909800	0.86862200
C	-0.94336900	-6.82151100	3.86540300
C	-0.73365700	-7.60574000	-0.70050600

C	-6.35573000	0.20527300	-2.53242700
C	6.55778200	-3.82892600	-1.51707200
C	4.58719800	-0.49208600	-4.08435600
C	-0.59992000	7.74048600	-3.24277100
C	0.94590400	8.43528700	1.07532300
C	-7.63802500	4.27266800	-0.68852000
H	0.00973500	-8.38088600	-0.91027700
H	-1.63250800	-8.07575100	-0.29751600
H	-0.98339800	-7.11844600	-1.64773900
H	-0.56333600	-6.12452800	4.61854000
H	-1.82971900	-7.31047400	4.28143700
H	-0.18150900	-7.57801300	3.66964700
H	-6.58034500	0.91079000	-3.33426400
H	-5.44514400	-0.34483600	-2.78782200
H	-7.17186000	-0.51981400	-2.45717200
H	-8.46109300	3.56616400	-0.56701700
H	-7.88578300	4.97140700	-1.49343200
H	-7.53009700	4.85253700	0.23313700
H	-0.61178900	6.96635600	-4.01578200
H	0.35472800	8.26781200	-3.28525200
H	-1.40644900	8.44581600	-3.46498000
H	7.34340900	-3.15779900	-1.86881500
H	6.59647200	-4.75604100	-2.09700300
H	6.75134500	-4.08331400	-0.47062700
H	3.61428200	-0.01083500	-4.22164300

H	5.35146700	0.18349200	-4.48054600
H	4.60580300	-1.42228900	-4.65476400
H	0.12670100	9.12817700	0.87605000
H	0.82907400	8.03260500	2.08607400
H	1.89121800	8.98581300	1.04306500
N	5.64231000	3.59604600	1.75520000
N	-5.48164900	-1.81582000	4.33416200
C	14.12471500	4.52635300	6.50550800
C	13.01623600	3.79959200	5.70108900
C	11.59140300	4.28340300	6.06952300
C	15.47819200	3.78357900	6.47457900
F	14.27983400	5.74957100	5.97539100
F	13.73863300	4.63768800	7.78896800
F	13.23770500	4.02545100	4.39381200
F	13.08586600	2.47825500	5.93972300
F	11.25131000	3.71358100	7.23824500
F	11.61067400	5.62067100	6.21831100
F	10.70171500	4.79602200	3.97241500
C	10.54048700	3.92929200	4.98713000
F	10.77895400	2.67820800	4.54692800
I	17.07240700	4.98163400	7.30255300
I	7.64400200	3.82789800	3.89337000
F	15.36898100	2.65540900	7.18176100
F	15.76642900	3.47140200	5.20415800
F	8.90032500	3.00016500	6.37172900

C 9.08525400 3.99673000 5.48983900

F 8.92653800 5.16829300 6.13656600

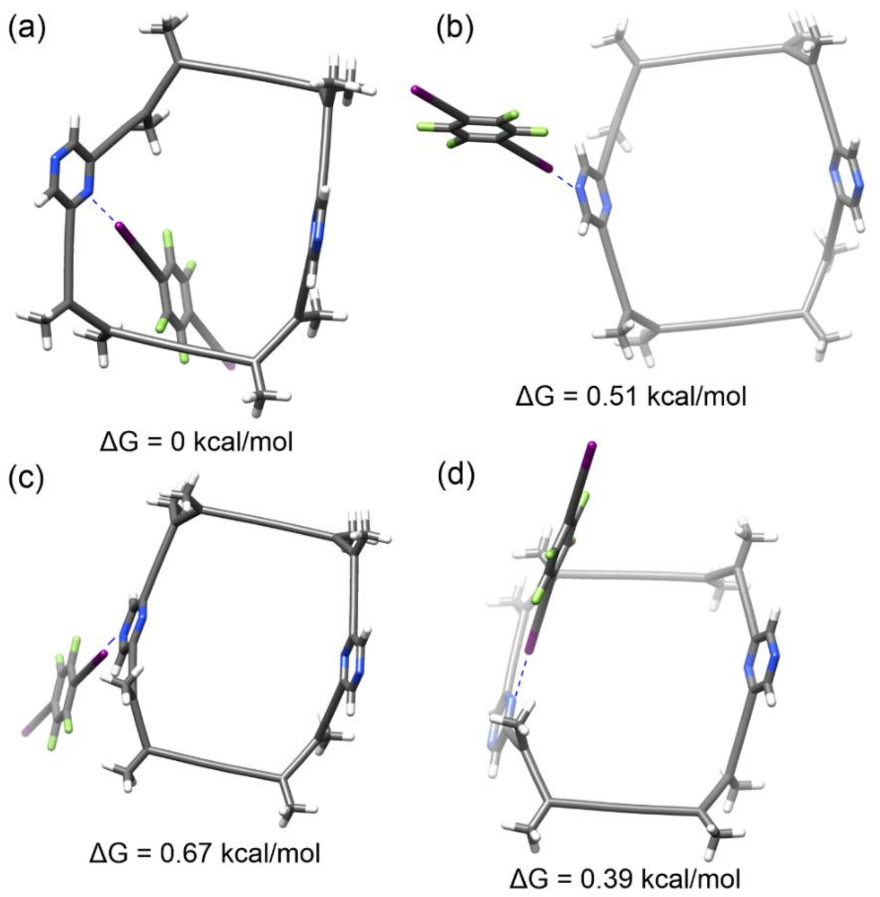

Figure S21. *Geometries of the* ***G2****@(*P_4*)-****2*** *halogen bond complex at the DFT CAM-B3LYP/6-31G* level using LANL08(d) basis set on the iodine atoms and their energy difference with respect to the most stable conformer (a). Solvation effects were taken into account using IEFPCM model with benzene parameters. Halogen bond interactions are represented as dashed blue lines.*

G2@(P_4)-**2** (a)

C 3.02514300 -5.02146500 -0.46775700

C 1.76316800 -5.55031000 -0.51151800

C 4.13141400 -4.52986200 -0.43304100

C 0.63890700 -5.99916400 -0.54380400

C 5.62626600 -0.43776000 0.11869700

C 5.52839200 0.42186400 0.96051100

C -3.01578300 -5.24757100 1.71202300

C	-3.66197800	-4.24465200	1.89948500
C	4.60506100	3.40921000	2.71988800
C	5.40859200	1.40903200	1.99009700
C	6.01234700	1.20455100	3.23904900
C	5.21819100	3.19294700	3.96224300
C	3.17155100	5.58614700	2.31585600
C	-0.25039100	6.55491100	-0.25091500
C	-1.35699600	6.08801700	-0.40622400
C	-2.59850800	5.53548100	-0.57205500
C	-3.68549700	5.02242000	-0.72007700
C	-5.11707800	0.89407400	-0.46416200
C	-5.07582700	-0.98167300	1.38606500
N	-4.41579900	-2.12124400	1.14010500
C	-5.71652200	-0.77139600	2.61509500
C	-4.38783400	-3.03622300	2.11884500
H	6.57826000	0.29814400	3.43366400
C	-4.97517700	4.42603100	-0.88505800
C	-5.09877400	1.87739100	-1.50512300
C	-2.23503400	-6.42721900	1.49350300
C	-0.69303400	-6.51286500	-0.63075300
C	5.44419800	-3.96345300	-0.38634900
C	5.72789600	-1.40998600	-0.92883500
C	2.37081000	6.75971500	2.13835000
C	1.06606800	7.09742900	-0.11555300
C	-5.05608600	3.15248500	-1.19547000

C	-1.46470600	-6.48733900	0.43085600
C	5.60267800	-2.68732400	-0.65418600
C	1.72100200	6.93803800	1.01128900
N	4.70639800	2.51622100	1.72983000
C	-5.04080100	-2.80489700	3.33806800
H	-5.01497600	-3.55564400	4.12217600
H	5.12906200	3.93046000	4.75478900
H	-6.24757000	0.15693700	2.80228300
C	3.83770600	4.59462400	2.49134100
C	-5.10582100	0.02248300	0.37137300
C	-2.32807100	-7.54260700	2.51352600
C	-1.15835100	-7.05319800	-1.96742700
C	-5.10227800	1.37586900	-2.93491200
C	6.59842300	-4.87615500	-0.02635800
C	5.96003400	-0.89606300	-2.33524400
C	1.65428200	7.82651200	-1.30662900
C	2.30221200	7.74616100	3.28599700
C	-6.19505900	5.30241400	-0.68934900
H	-0.52494300	-7.89007500	-2.27756900
H	-2.19309100	-7.39505300	-1.90431600
H	-1.08479100	-6.27761100	-2.73403700
H	-2.03073900	-7.18010000	3.50239400
H	-3.35671300	-7.90892700	2.58819500
H	-1.67768000	-8.37309900	2.23394700
H	-5.05497700	2.21104200	-3.63569600

H	-4.24400800	0.71893300	-3.10458200
H	-6.00843000	0.79536100	-3.13332200
H	-7.11136100	4.72777900	-0.83626700
H	-6.18123500	6.13590300	-1.39820000
H	-6.19928000	5.72478800	0.32002300
H	1.69937300	7.15891200	-2.17225800
H	2.66147800	8.18205400	-1.08217200
H	1.02711100	8.68143200	-1.57735700
H	7.54027300	-4.32448500	-0.02377700
H	6.67081800	-5.69907500	-0.74404600
H	6.44101000	-5.31275300	0.96460200
H	5.15056100	-0.21988200	-2.62600600
H	6.89738600	-0.33360100	-2.38595000
H	6.00320500	-1.72315600	-3.04598600
H	1.65615400	8.58865000	3.03318000
H	1.90967000	7.25700500	4.18272900
H	3.30125800	8.12459400	3.52363000
N	5.91929800	2.09544100	4.21910700
N	-5.70153200	-1.68061000	3.58180900
C	-1.40855600	-3.39179100	-8.09660300
C	-1.91480100	-3.07973300	-6.84871500
C	-1.49639400	-3.76146900	-5.70231300
C	-0.54455400	-4.77074300	-5.87054600
C	-0.03812900	-5.08479500	-7.11735200
C	-0.45743100	-4.40190700	-8.26205600

F	-1.84082500	-2.71294500	-9.15684000
F	-2.81941900	-2.10844400	-6.74644000
F	0.86546900	-6.05658400	-7.22198400
F	-0.10788800	-5.45364800	-4.81124400
C	0.06407500	-4.72213200	-9.54441100
C	-2.01909500	-3.44235100	-4.42154300
C	0.50762600	-4.99404300	-10.63243800
C	-2.46533200	-3.16924100	-3.33229800
I	1.24105700	-5.43942300	-12.42174500
I	-3.21745900	-2.72005500	-1.53176300

G2@(P_4)-**2** (b)

C	2.75638500	-5.36528500	-0.78892500
C	1.50294100	-5.91165500	-0.72172900
C	3.85465000	-4.85839600	-0.84951600
C	0.38623100	-6.37525100	-0.65364600
C	5.33519800	-0.75347000	-0.38482000
C	5.29612700	0.09472100	0.47315700
C	-3.18410500	-5.46902300	1.71989500
C	-3.85371400	-4.47775200	1.88362100
C	4.50296300	3.05928300	2.33156300
C	5.24850500	1.06732900	1.52239600
C	5.93132600	0.84213900	2.72606400
C	5.19487000	2.82251500	3.52812900
C	3.05483500	5.24599700	2.05245600
C	-0.48911100	6.28262200	-0.31626800

C	-1.60396600	5.82281600	-0.42750700
C	-2.85524200	5.27988400	-0.54457200
C	-3.95168400	4.77661200	-0.65049700
C	-5.42628100	0.65437800	-0.42902500
C	-5.35862200	-1.25719900	1.38568000
N	-4.65098400	-2.36407300	1.13927700
C	-6.04735100	-1.08589400	2.59451200
C	-4.62395800	-3.29396200	2.09964200
H	6.50456100	-0.06904500	2.87044900
C	-5.25380900	4.19588700	-0.76604900
C	-5.44012800	1.66578800	-1.44344800
C	-2.38149500	-6.64325500	1.55727400
C	-0.93857200	-6.91289200	-0.62031500
C	5.15933900	-4.27493400	-0.91206400
C	5.36108900	-1.71247300	-1.44885200
C	2.24809700	6.42386600	1.94434400
C	0.83387600	6.81971700	-0.23275700
C	-5.36442700	2.93169200	-1.10434600
C	-1.66271000	-6.78760700	0.46755700
C	5.27703800	-2.99430800	-1.17961500
C	1.54372300	6.63065400	0.85558600
N	4.53576500	2.18062500	1.32423500
C	-5.31924600	-3.11686800	3.30405800
H	-5.29600000	-3.87813000	4.07745000
H	5.16074800	3.54852000	4.33541400

H	-6.61911300	-0.18358500	2.78659300
C	3.72726600	4.25022700	2.17053700
C	-5.39678900	-0.23514700	0.38648600
C	-2.39240300	-7.66566500	2.67504800
C	-1.45449900	-7.59874300	-1.86908600
C	-5.52097200	1.20586600	-2.88487200
C	6.35176800	-5.17515400	-0.66161000
C	5.46685900	-1.18034500	-2.86356600
C	1.36322700	7.57995300	-1.43198400
C	2.23760600	7.38099800	3.11850400
C	-6.45306500	5.07843700	-0.48753900
H	-0.81510900	-8.44840700	-2.12717800
H	-2.47550700	-7.95480400	-1.72075700
H	-1.44096500	-6.90291500	-2.71323500
H	-2.05466800	-7.20880100	3.61026700
H	-3.40639000	-8.04454400	2.83633600
H	-1.73604100	-8.50417400	2.43631100
H	-5.50754500	2.06084800	-3.56295100
H	-4.67459600	0.55344400	-3.11940800
H	-6.43856000	0.63354300	-3.05209400
H	-7.38172000	4.51691900	-0.60446600
H	-6.46468100	5.93097100	-1.17342000
H	-6.40331500	5.47284800	0.53184500
H	1.36199500	6.93641600	-2.31681000
H	2.38166900	7.92738200	-1.24949000

H	0.72591000	8.44320600	-1.64677600
H	7.28304700	-4.61036600	-0.73311100
H	6.37372100	-5.98987600	-1.39186100
H	6.28513500	-5.62351700	0.33431100
H	4.62730300	-0.51200500	-3.07680300
H	6.38927500	-0.60446000	-2.98600700
H	5.46065800	-1.99880100	-3.58551900
H	1.58074800	8.22949000	2.91937300
H	1.88932800	6.86928400	4.02088000
H	3.24762900	7.75340600	3.31578000
N	5.90630600	1.71894300	3.72278000
N	-6.02550500	-2.01724300	3.54199500
C	-11.24351200	-1.58555100	10.03220900
C	-11.96272400	-1.44845000	11.20463500
C	-11.36327700	-0.96421700	12.37017300
C	-10.00966400	-0.62379400	12.30532900
C	-9.29084600	-0.76107000	11.13259600
C	-9.88940300	-1.24547900	9.96617800
F	-11.86111900	-2.05180800	8.94924000
F	-13.25031900	-1.78633200	11.21608900
F	-8.00364400	-0.42277000	11.12347600
F	-9.39400700	-0.15777200	13.38968600
C	-9.15185000	-1.38650700	8.76076300
C	-12.10154000	-0.82425200	13.57621900
C	-8.52672700	-1.50722200	7.73458900

C	-12.72724300	-0.70559400	14.60035000
I	-7.49582500	-1.70799700	6.02529100
I	-13.75381100	-0.51303700	16.28764600

G2@(P_4)-**2** (c)

C	-0.71560700	0.03275300	6.28183300
C	0.61208600	-0.29936500	6.29736600
C	-1.88921500	0.32871200	6.23813800
C	1.78657300	-0.59475400	6.28057000
C	-4.76322100	0.66017800	2.95713300
C	-5.17465700	-0.07654600	2.09389800
C	4.78765200	-0.78415800	3.09296800
C	5.23767000	-0.01669100	2.27713600
C	-5.80464600	-1.61998600	-1.08582400
C	-5.65050600	-0.98646800	1.09900400
C	-6.39898400	-2.11080400	1.47379500
C	-6.55659600	-2.73964300	-0.70316100
C	-5.19393400	-1.24174400	-3.62206200
C	-1.73611900	0.50541600	-5.67710800
C	-0.54318100	0.29653800	-5.68036000
C	0.80305200	0.04946000	-5.66565300
C	1.99538100	-0.16095800	-5.63546400
C	5.40426000	1.44214800	-3.37028800
C	5.97515100	1.66511000	-0.80481700
N	5.47889900	0.75723500	0.04214000
C	6.76248600	2.73261800	-0.34990900

C	5.76279200	0.92672000	1.33728600
H	-6.63063000	-2.30307900	2.51658600
C	3.39290100	-0.46125700	-5.59844400
C	5.09041600	1.36104300	-4.76493800
C	4.24083400	-1.74088200	4.00869500
C	3.17578800	-0.93430200	6.26934500
C	-3.27782200	0.66727200	6.19240800
C	-4.25965200	1.57907000	3.93404600
C	-4.84890200	-1.07398000	-5.00141600
C	-3.13476800	0.80177500	-5.66516700
C	4.24563300	0.44927000	-5.18839500
C	3.72563700	-1.33965600	5.14715600
C	-3.78683700	1.12611800	5.07181200
C	-3.99687400	-0.13420600	-5.34187900
N	-5.35793500	-0.74168300	-0.18200400
C	6.54809400	2.00146900	1.77780300
H	6.76786100	2.12456800	2.83433500
H	-6.91846400	-3.44686200	-1.44283700
H	7.16054100	3.45987500	-1.05178400
C	-5.48492000	-1.40494600	-2.46167500
C	5.67273600	1.53362100	-2.19672800
C	4.26730400	-3.19753400	3.59237700
C	3.94980500	-0.80479400	7.56497500
C	5.75361000	2.35577300	-5.69540100
C	-4.09993300	0.47457100	7.44986300

C	-4.27798500	3.05235400	3.58092600
C	-3.55903600	2.21237500	-6.02100600
C	-5.48332300	-2.01203900	-6.00755300
C	3.82553600	-1.84756000	-6.03185800
H	-3.69141000	1.07731100	8.26666200
H	-5.13845700	0.76469200	7.28074700
H	-4.07127200	-0.57327200	7.76353500
H	4.99370200	-1.08884500	7.42037100
H	3.91054400	0.22654100	7.92850300
H	3.51124100	-1.44572600	8.33592800
H	6.84240400	2.25663900	-5.64604200
H	5.43048000	2.19577200	-6.72547400
H	5.49888600	3.37834300	-5.40044300
H	5.29737200	-3.52824600	3.42784900
H	3.81050800	-3.82631900	4.35857200
H	3.72194900	-3.32923300	2.65311400
H	-3.69597400	3.22828200	2.67136900
H	-5.30237100	3.38683500	3.39057200
H	-3.85548300	3.64752000	4.39222300
H	-5.22998600	-3.05000700	-5.77099400
H	-6.57356900	-1.92074300	-5.98058000
H	-5.13501000	-1.78652500	-7.01687100
H	-3.22695000	2.46480300	-7.03256700
H	-4.64430900	2.31475200	-5.96725100
H	-3.10103500	2.92771200	-5.33149400

H	4.90846200	-1.95645300	-5.95068300
H	3.34569500	-2.60497900	-5.40491600
H	3.52550600	-2.03325000	-7.06767400
N	7.04765800	2.89839300	0.93584400
N	-6.84841600	-2.97614300	0.57110600
C	-11.39631700	-9.87132700	1.80221900
C	-12.11334200	-10.98256100	2.20453500
C	-12.26250400	-11.29812800	3.55748200
C	-11.65978700	-10.44763600	4.48797100
C	-10.94289300	-9.33657600	4.08514800
C	-10.79255600	-9.02024100	2.73206200
F	-11.28351500	-9.61456200	0.50098000
F	-12.66927500	-11.76244100	1.27991400
F	-10.38762200	-8.55830400	5.01131100
F	-11.77355000	-10.70646500	5.78882700
C	-10.05417800	-7.87971700	2.31883200
C	-12.99794300	-12.44110500	3.97205500
C	-9.42515900	-6.91012700	1.96860600
C	-13.62109500	-13.41118900	4.32569700
I	-8.37559900	-5.30135600	1.38955000
I	-14.64270100	-15.00862400	4.91083500

G2@(P_4)-**2** (d)

C	-0.56335400	0.05021700	6.40620900
C	0.74305800	-0.35753800	6.42519500
C	-1.71796400	0.41295500	6.35953700

C	1.89880700	-0.71953000	6.41144400
C	-4.58895900	0.80650200	3.08442500
C	-5.04493100	0.07161500	2.24184100
C	4.87643600	-1.13801700	3.22304700
C	5.36604500	-0.41225500	2.39204100
C	-5.79795600	-1.46703400	-0.91911700
C	-5.56329000	-0.83251400	1.26577000
C	-6.30390500	-1.95616400	1.65868300
C	-6.53994600	-2.58122800	-0.50185800
C	-5.24847800	-1.07036200	-3.46449000
C	-1.70058300	0.54360700	-5.42833000
C	-0.52619500	0.24884200	-5.45251100
C	0.79878100	-0.09436900	-5.46330900
C	1.97332500	-0.38966500	-5.45640800
C	5.53077700	0.98457400	-3.27144300
C	6.16534100	1.18530400	-0.71927300
N	5.62168900	0.32241800	0.14542700
C	7.03523300	2.19762800	-0.28873500
C	5.94033500	0.48288200	1.43360600
H	-6.49599800	-2.14275600	2.71089200
C	3.34687100	-0.78657900	-5.44565300
C	5.18375100	0.91757800	-4.65897300
C	4.27972100	-2.04501700	4.15810800
C	3.26681000	-1.13648500	6.40398900
C	-3.08437500	0.83174700	6.31010000

C	-4.02700800	1.72583100	4.02798900
C	-4.91148900	-0.85570500	-4.83915000
C	-3.07415000	0.93858200	-5.38970500
C	4.26953100	0.06406000	-5.05907300
C	3.79010300	-1.59400500	5.28914600
C	-3.57404700	1.28209700	5.17751800
C	-3.99834900	0.04267600	-5.13137500
N	-5.31743100	-0.58856600	-0.02824900
C	6.80805200	1.50234100	1.84961600
H	7.05503100	1.61887800	2.90086900
H	-6.92770200	-3.28393000	-1.23328400
H	7.47107600	2.88851300	-1.00476400
C	-5.52088500	-1.25199400	-2.30199400
C	5.82827900	1.06464600	-2.10403500
C	4.22469500	-3.50868100	3.77024500
C	4.05087000	-1.02420000	7.69521300
C	5.89387300	1.86002100	-5.60904700
C	-3.90668400	0.73133300	7.57815900
C	-3.96048500	3.18461100	3.62377200
C	-3.39812000	2.39757600	-5.63837200
C	-5.62105200	-1.68837800	-5.88615800
C	3.67338400	-2.20170700	-5.87872300
H	-3.45747300	1.33746300	8.37073600
H	-4.92772500	1.07591100	7.40474200
H	-3.93670400	-0.30473800	7.92875700

H	5.07675900	-1.36925000	7.55439300
H	4.07067200	0.01496800	8.03738900
H	3.57954000	-1.62353300	8.48019200
H	6.97416700	1.68665800	-5.58207400
H	5.53838300	1.71794800	-6.63104700
H	5.71600800	2.89900500	-5.31513400
H	5.23456600	-3.89880800	3.61061900
H	3.73590100	-4.09641400	4.54921000
H	3.67058500	-3.62813800	2.83446700
H	-3.38672000	3.29232600	2.69852400
H	-4.96563100	3.57557200	3.43855600
H	-3.48703200	3.77916400	4.40682500
H	-5.44022000	-2.75370500	-5.71256400
H	-6.70184000	-1.52209100	-5.84042600
H	-5.26681800	-1.43056900	-6.88563200
H	-3.03724700	2.70162200	-6.62570900
H	-4.47534400	2.56588800	-5.58599000
H	-2.90312200	3.02636200	-4.89385700
H	4.74776400	-2.38481900	-5.82031700
H	3.15633200	-2.92076600	-5.23634100
H	3.33837800	-2.37180700	-6.90648300
N	7.35519200	2.35367800	0.98993600
N	-6.79184600	-2.82127100	0.77841200
C	-0.73267600	7.09639700	-4.05955100
C	-1.33753200	5.92524400	-3.64441900

C	-1.90643700	5.80874700	-2.37336900
C	-1.83822200	6.92689400	-1.53718800
C	-1.23298700	8.09890100	-1.95049600
C	-0.66491000	8.21276700	-3.22195200
F	-0.20704600	7.15466400	-5.28110700
F	-1.37372100	4.89009500	-4.48485200
F	-1.19451700	9.13494900	-1.11570400
F	-2.36445400	6.87114500	-0.31570600
C	-0.04215200	9.41718100	-3.64680500
C	-2.52805400	4.60617100	-1.94562500
C	0.48659700	10.43947100	-4.00709000
C	-3.05691000	3.58490400	-1.57483600
I	1.35936400	12.12100100	-4.59773100
I	-3.93499400	1.89850300	-0.94688500

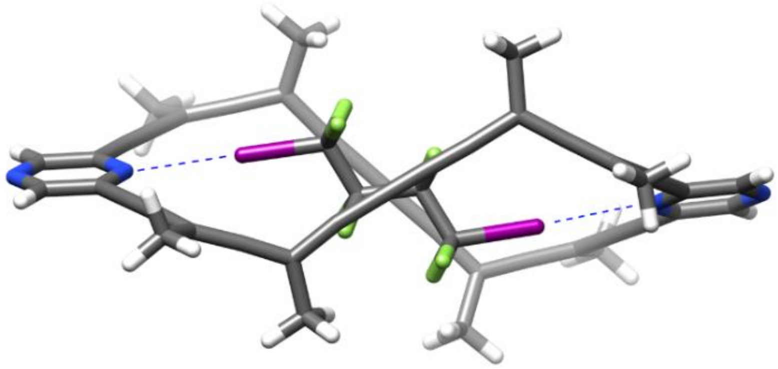

Figure S22a. *Geometry of the **G3**@(P_4)-**2** halogen bond complex at the DFT CAM-B3LYP/6-31G* level using LANL08(d) basis set on the iodine. Solvation effects were taken into account using IEFPCM model with benzene parameters. Halogen bond interactions are represented as dashed blue lines (N···I bond 2.96 Å, C-I···N bond angle of 177.1º).*

G3@(P_4)-**2**

C	-0.08941400	4.11675000	-0.48644000
C	0.68163500	4.21169700	0.64082700
C	-0.78138000	4.02215700	-1.47607300
C	1.33511500	4.28121700	1.65823300
C	-0.90472800	3.03281100	-5.87954900
C	-0.66021800	2.07409200	-6.57271700
C	0.89702400	3.21514500	5.95211400
C	0.67747100	2.20201200	6.57178700
C	0.19809800	-1.31885000	-7.38670200
C	-0.39795000	0.89380800	-7.33409400
C	-0.55498200	0.86971900	-8.72726000
C	0.02908300	-1.31921200	-8.77780300
C	0.92901200	-3.42825700	-5.98891900
C	1.10165100	-4.02360600	-1.60952200
C	0.37471700	-4.01018100	-0.64080800
C	-0.45702900	-3.98631800	0.44677500
C	-1.18255700	-3.95739600	1.41615500
C	-0.96431700	-3.29285700	5.79257900
C	-0.16939200	-1.22972800	7.22545700
N	0.01216300	-0.07835500	6.56635200
C	0.06885400	-1.30981000	8.60470900
C	0.44257200	0.97645500	7.26892200
H	-0.86265300	1.76437200	-9.26038200
C	-2.11431700	-3.89354100	2.49868700

C	-1.39901100	-4.38722200	4.97753700
C	1.14549200	4.43154400	5.23628000
C	2.16833200	4.34222900	2.81817900
C	-1.74748700	3.86540700	-2.51804400
C	-1.23403100	4.17195700	-5.07562100
C	1.33488300	-4.52635900	-5.16362500
C	2.04008200	-4.00298800	-2.68798900
C	-1.74131100	-4.15479200	3.73006300
C	1.64315500	4.38374200	4.02146300
C	-1.45205800	4.02136600	-3.78793500
C	1.67121700	-4.28455300	-3.91614600
N	-0.01348300	-0.20528100	-6.67427800
C	0.67023300	0.87263100	8.64819300
H	1.01466400	1.73358600	9.21328800
H	0.20130500	-2.22445100	-9.35239000
H	-0.08244100	-2.24598600	9.13385500
C	0.59661900	-2.48892500	-6.67174200
C	-0.60497400	-2.36488500	6.47715100
C	0.81906400	5.73542200	5.93634600
C	3.66826700	4.34721000	2.59499900
C	-1.45041000	-5.76469200	5.60514300
C	-3.15086200	3.50109000	-2.06685400
C	-1.34985000	5.51298700	-5.77204100
C	3.46701100	-3.61626300	-2.34928400
C	1.36397500	-5.91043200	-5.77695700

C	-3.53767200	-3.49331200	2.16104400
H	-2.12370600	-5.76742800	6.46808200
H	-1.80075400	-6.50302400	4.88190900
H	-0.45723200	-6.06065000	5.95687800
H	-4.16729300	-3.51475400	3.05239600
H	-3.95559300	-4.16998200	1.40989500
H	-3.54134000	-2.48178900	1.74676700
H	2.04343400	-5.93509900	-6.63471100
H	0.36792800	-6.19087100	-6.13327000
H	1.69462500	-6.64798700	-5.04371800
H	-3.81431000	3.37153000	-2.92377700
H	3.95716600	5.20564500	1.98107800
H	4.20122200	4.39442500	3.54633500
H	1.05378600	6.58555500	5.29359500
H	1.39151200	5.82452000	6.86498600
H	3.48054500	-2.59745000	-1.95305600
H	-0.24355300	5.77210000	6.19513600
H	3.87145000	-4.28411000	-1.58299600
H	4.10172900	-3.66241700	-3.23605200
H	-3.11668000	2.57009300	-1.49425000
H	-3.55425700	4.28165600	-1.41481900
H	-1.62551100	6.29273900	-5.06004600
H	-0.39751900	5.78353000	-6.23845000
H	-2.10692300	5.46985500	-6.56132800
H	3.97029400	3.44015800	2.06325600

N	0.48067700	-0.26306800	9.30916300
N	-0.34094600	-0.22948100	-9.44011500
C	1.04197100	0.17449000	-1.69365900
C	-0.21452100	0.03881100	-0.81113800
C	0.08102500	0.09735400	0.70850600
C	-1.16593600	0.35119700	1.57841100
I	-0.71936100	0.15868100	3.67118200
I	0.59661700	-0.08184100	-3.77723200
F	-1.62214600	1.58926900	1.31892400
F	-2.12368100	-0.52843300	1.20871700
F	0.62998100	-1.08017500	1.05585300
F	0.96320700	1.08251100	0.96009200
F	-1.06848800	1.02952200	-1.14149400
F	-0.80470600	-1.14150400	-1.06717900
F	1.94187900	-0.74353800	-1.27496800
F	1.57418200	1.39346800	-1.50155900

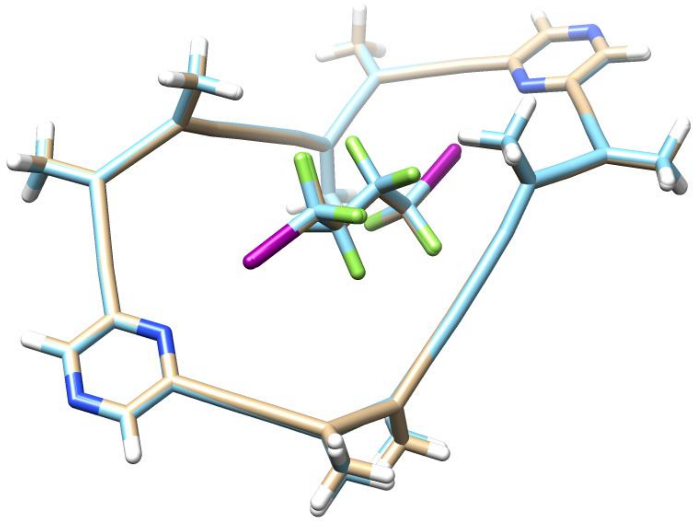

Figure S22b. *Comparison of the calculated geometries of the G3@(P4)-2 halogen-bond complex. The gold structure corresponds to the DFT-optimized geometry at the CAM-B3LYP/6-31G level with the LANL08(d) basis set on iodine; the cyan structure corresponds to the B3LYP-D3/6-31G+(d,p) level with the LANL08(d) basis set on iodine. Solvation effects were included using the IEFPCM model with benzene parameters.*

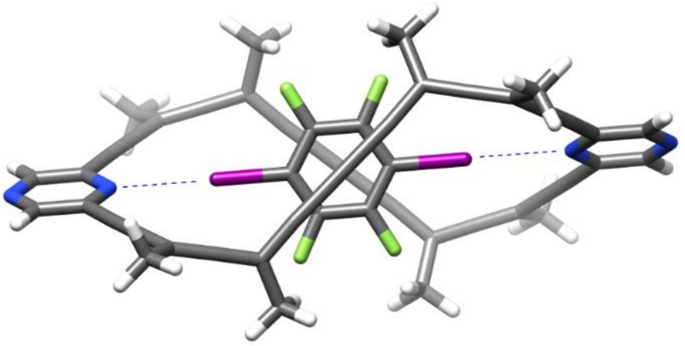

Figure S23. *Geometries of the **G4**@(P$_4$)-**2** halogen bond complex at the DFT CAM-B3LYP/6-31G* level using LANL08(d) basis set on the iodine atoms. Solvation effects were taken into account using IEFPCM model with benzene parameters. Halogen bond interactions are represented as dashed blue lines (N···I bond 3.01 Å, C-I···N bond angle of 180º).*

G4@(P_4)-**2**

C	-0.26855000	3.76595100	-0.54246400
C	0.63603700	3.73415100	0.48518700
C	-1.06125800	3.77111200	-1.45868900
C	1.42671400	3.68353400	1.40178400
C	-1.10152100	3.18181300	-5.82078600
C	-0.75571500	2.25211000	-6.50968000
C	1.41636600	3.06403100	5.75927700
C	1.00930700	2.15446600	6.44154700
C	0.40707500	-1.05862200	-7.26230100
C	-0.34678000	1.10288900	-7.25250700
C	-0.34614800	1.09633500	-8.65423300
C	0.39569500	-1.04315200	-8.66389100
C	1.17187100	-3.14649600	-5.84909200
C	1.16054000	-3.76325000	-1.49049100
C	0.37400500	-3.76408200	-0.56894000
C	-0.52376800	-3.73902000	0.46485500
C	-1.30852500	-3.69433900	1.38683700
C	-1.27156900	-3.10156500	5.74816400
C	-0.37050600	-1.07684100	7.17297800
N	0.07429500	-0.02093900	6.48028800
C	-0.35813700	-1.07120200	8.57465100
C	0.52468100	1.03006800	7.17682400
H	-0.65382000	1.97884000	-9.20727400
C	-2.30041800	-3.57742500	2.41007200
C	-1.76376900	-4.15750600	4.91479800
C	1.90362400	4.12508500	4.92950800
C	2.42494100	3.56014200	2.41809000
C	-2.06707100	3.71751500	-2.47355100
C	-1.51782700	4.26619300	-4.98259200
C	1.59379000	-4.23599100	-5.02039200
C	2.15958900	-3.70292500	-2.51165700
C	-2.02378500	-3.89127400	3.65449700
C	2.15593900	3.86658600	3.66603200
C	-1.78239500	4.01558400	-3.72003000

C	1.86669800	-3.99311600	-3.75808900
N	0.03285600	0.01994200	-6.56282100
C	0.52322700	1.01467100	8.57847000
H	0.88837100	1.87139500	9.13705400
H	0.69903800	-1.92211000	-9.22491100
H	-0.71882500	-1.93185800	9.13007700
C	0.82140800	-2.21255500	-6.52985100
C	-0.86028000	-2.19637000	6.43369700
C	2.10938200	5.48835300	5.55487400
C	3.77702700	3.02283500	1.98980700
C	-1.96549300	-5.52465400	5.53297300
C	-3.44937700	3.26331500	-2.04544200
C	-1.63864300	5.64389800	-5.59841400
C	3.54469400	-3.25155600	-2.08968100
C	1.71064600	-5.60984500	-5.64548600
C	-3.65528900	-3.03801300	1.99331200
H	-2.67637600	-5.46799800	6.36327100
H	-2.34503900	-6.22935100	4.79114400
H	-1.01988500	-5.90662800	5.92989800
H	-4.33874800	-3.01069000	2.84388900
H	-4.08835000	-3.66116700	1.20526200
H	-3.53583300	-2.02482100	1.59979500
H	2.42701300	-5.59408600	-6.47285000
H	0.74426300	-5.92875200	-6.04801100
H	2.04161200	-6.34126400	-4.90626200
H	-4.14054500	3.28028000	-2.89003600
H	4.20558600	3.65066600	1.20300800
H	4.46559400	2.99002900	2.83606000
H	2.48465700	6.19751100	4.81512900
H	2.82520000	5.42644100	6.38054500
H	3.48828300	-2.23484400	-1.69114900
H	1.16625900	5.86807900	5.95978600
H	3.93623000	-3.90463300	-1.30413300
H	4.23027300	-3.26281700	-2.93891400
H	-3.39034500	2.24392000	-1.65420800

H	-3.83571000	3.91107400	-1.25294600
H	-1.96476100	6.37074300	-4.85255400
H	-0.67487200	5.96525800	-6.00522400
H	-2.36037000	5.63326600	-6.42118800
H	3.65476000	2.01212000	1.59083800
N	0.08520300	-0.03066300	9.26968100
N	0.02213000	0.02881600	-9.35216800
C	1.23920000	-0.03584600	0.64826400
C	1.23556700	-0.03421500	-0.73677000
C	0.05009000	0.00343300	-1.45567600
C	-1.13165600	0.03687600	-0.73042800
C	-1.12816200	0.03049200	0.65459900
C	0.05739900	-0.00476300	1.37347200
I	0.06310900	-0.01071900	3.45543900
I	0.04439800	0.00943100	-3.53764200
F	2.42280200	-0.06362600	1.27355100
F	2.41556100	-0.07565700	-1.36822300
F	-2.30853800	0.05454200	1.28606000
F	-2.31485400	0.08207900	-1.35558300

Table S5. $V_{S,max}$ (kcal/mol) on the molecular surfaces associated with σ-holes of Iodine atoms in C-I bonds.

Compound	ωB97X-D/Def2TZVPP	ωB97X-D/ aug-cc-pVTZ/ aug-cc-pVTZ-PP	B3LYP-D3/ aug-cc-pVTZ/ aug-cc-pVTZ-PP
**G1**	+33.0	+31.8	+31.0
**G2**	+39.4	+40.7	+42.0
**G3**	+32.6	+31.3	+30.5
**G4**	+32.8	+32.8	+32.3

4.1. Computed ECD vs Experimetal ECD

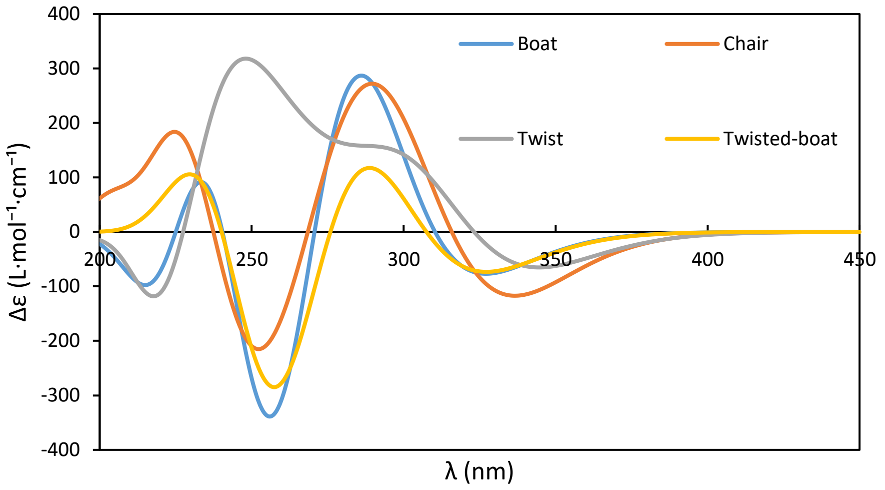

Figure S24. *Computed ECD spectra of (P$_4$)-2 for each of its conformers (TD-DFT at the CAMB3LYP/6-31G+(d,p), smd = chloroform level).*

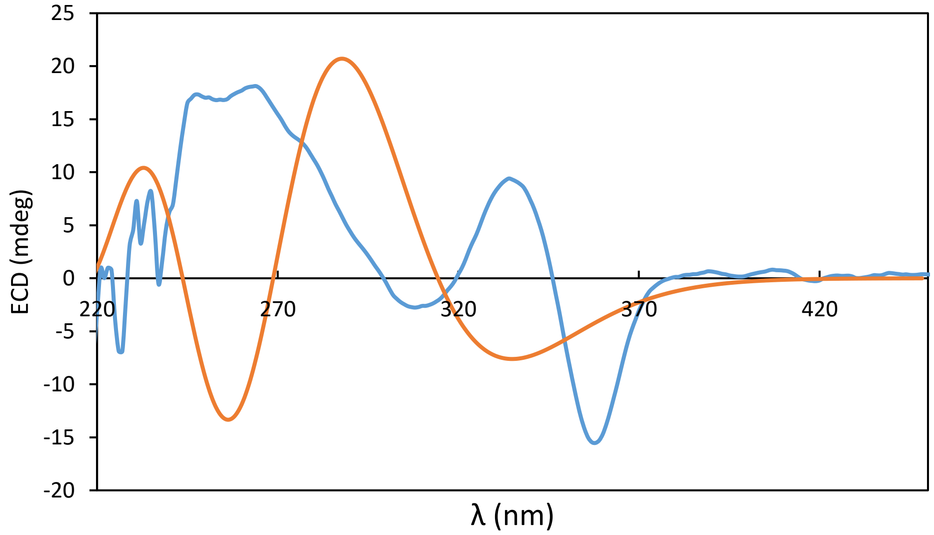

Figure S25. *Comparison between experimental ECD spectra of (P$_4$)-**2** (blue line, 6.24 x 10^{-6} M in chloroform) and calculated ECD spectrum assuming an equal population of the four previously presented conformers (orange line, TD-DFT at the CAMB3LYP/6-31G+(d,p), smd = chloroform).*

5. Titration procedures

The complexation processes between (P_4)-**2** and the halogen bond donors (HB-D) were studied using ^{1}H NMR and ^{1}H-^{15}N gradient-selected-HMBC titrations employing C_6D_6 as solvent. (P_4)-**2** was dissolved in 400 μL of the designated solvent and incremental additions of neat halogen bond donor were made to the sample. The obtained data was analyzed using *BindFit* v0.5 online software package[30] a 1:1 global fitting model (Nelder-Mead method).

G1 (Dodecafluoro-1,6-diiodohexane)

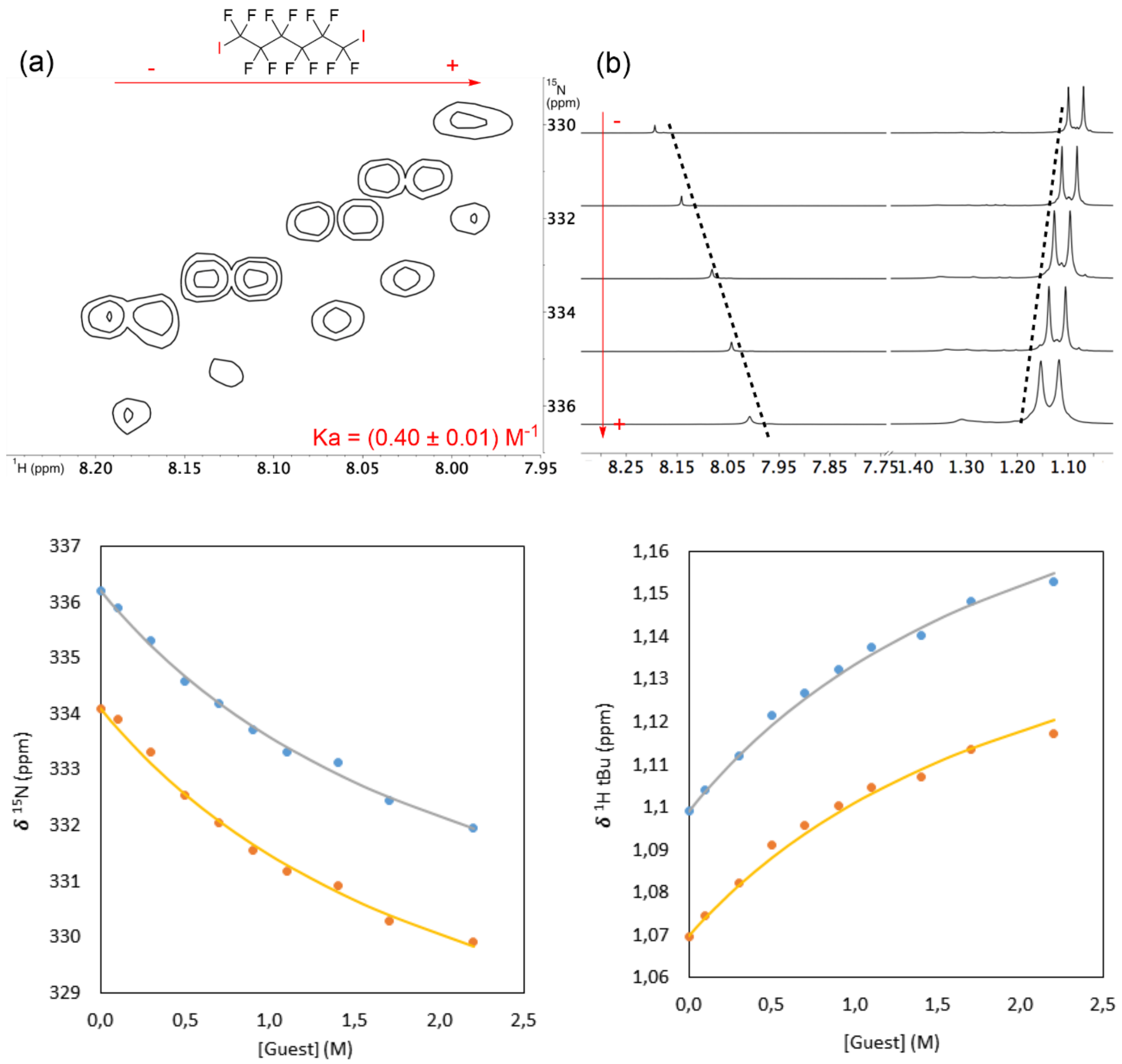

Figure S26. *Top) NMR titration experiments of (P₄)-**2** with **G1**, from 0 to 220 equivalents, and its association constant value (a)* ^{1}H-^{15}N *HMBC and (b)* ^{1}H *NMR. Bottom) Experimental variation of chemical shift (ppm) in the NMR spectrum of (*P_4*)-**2** upon the addition of **G1** (dots) and mathematical fitting to a 1:1 stoichiometric equilibrium (lines).*

G2 (1,2,4,5-tetrafluoro-3,6-bis(iodoethynyl)benzene)

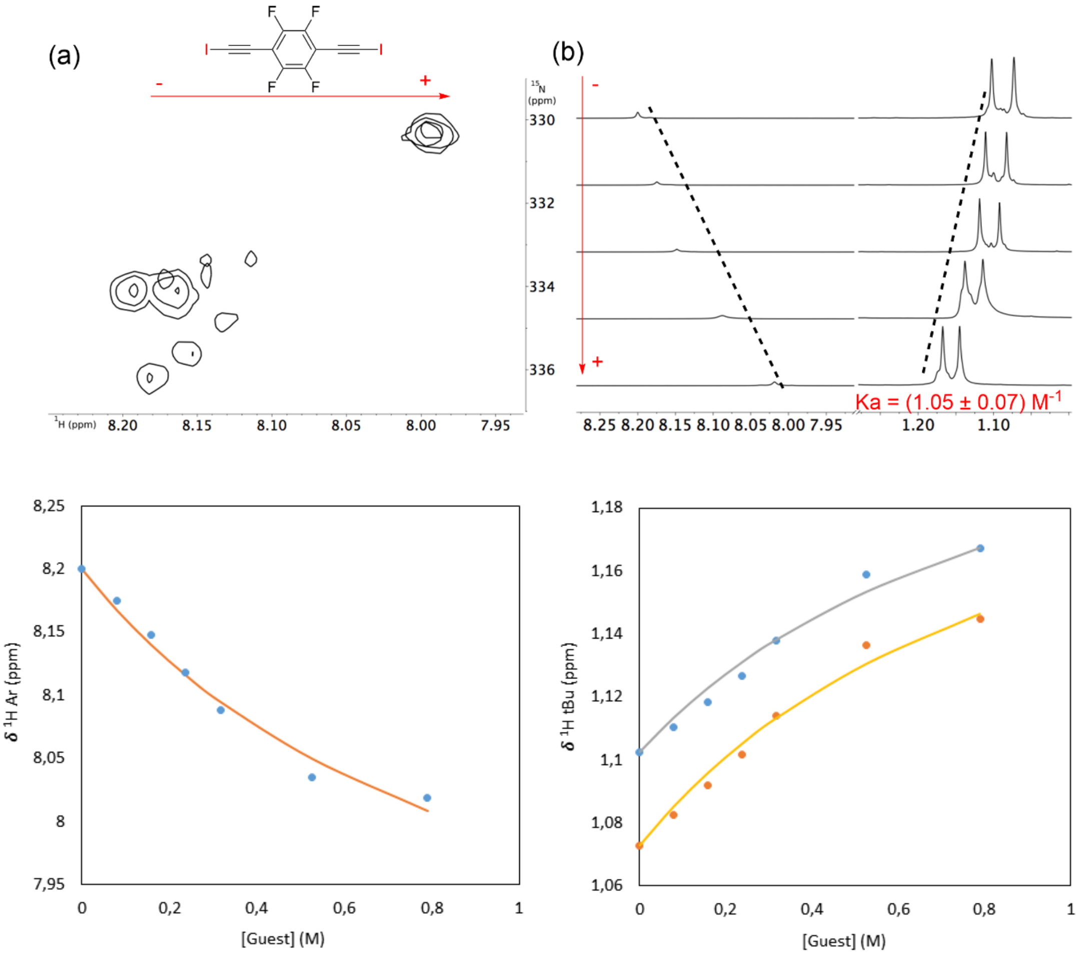

Figure S27. *Top) NMR titration experiments of (P*$_4$*)-**2** with **G2**, from 0 to 75 equivalents, and its association constant value. (a)* ^{1}*H-*^{15}*N HMBC and (b)* ^{1}*H NMR*. *Bottom) Experimental variation of chemical shift (ppm) in the* ^{1}*H-NMR spectrum of (P*$_4$*)-**2** upon the addition of **G2** (dots) and mathematical fitting to a 1:1 stoichiometric equilibrium (lines).*

G3 (Octafluoro-1,4-diiodobutane)

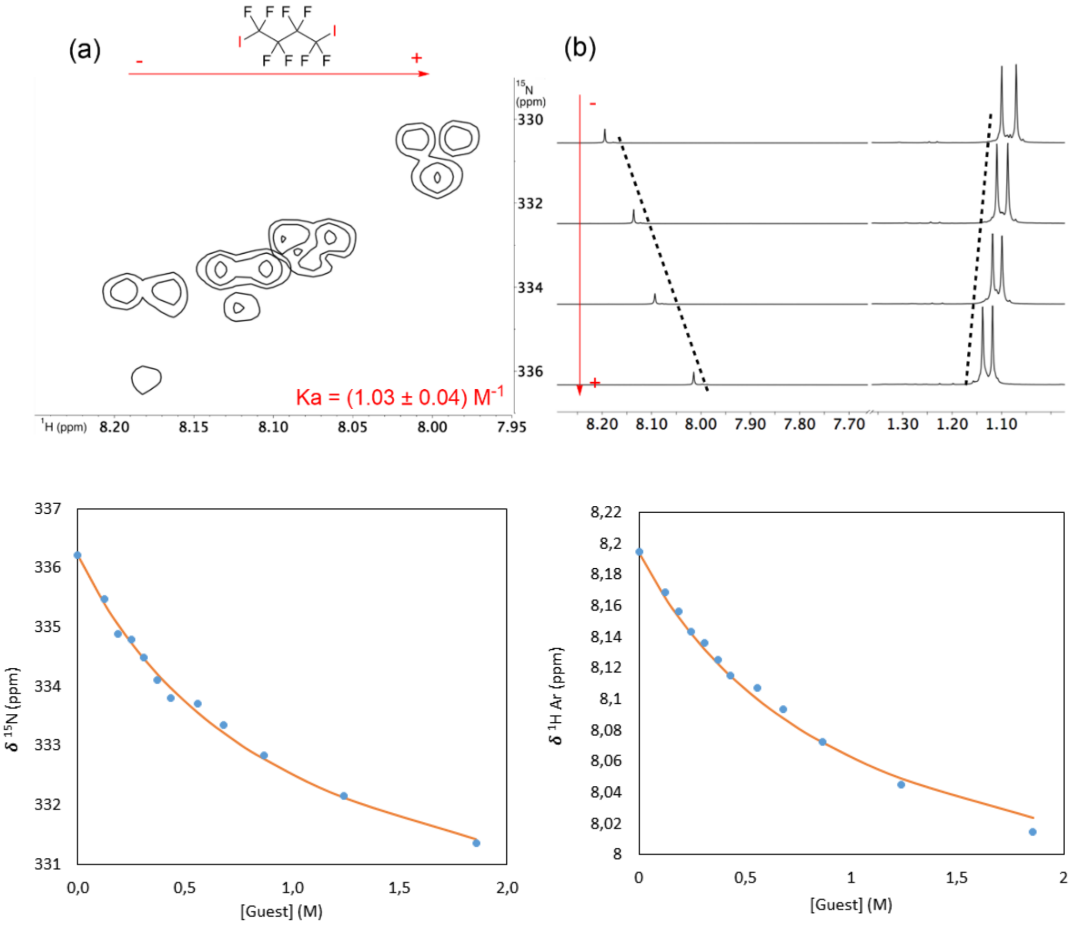

***Figure S28**. Top) NMR titration experiments of (P$_4$)-**2** with **G3**, from 0 to 150 equivalents, and its association constant value. (a) ^{1}H-^{15}N HMBC and (b) ^{1}H NMR. Bottom) Experimental variation of chemical shift (ppm) in the NMR spectrum of (P$_4$)-**2** upon the addition of **G3** (dots) and mathematical fitting to a 1:1 stoichiometric equilibrium (lines).*

G4 (1,2,4,5-tetrafluoro-3,6-diiodobenzene)

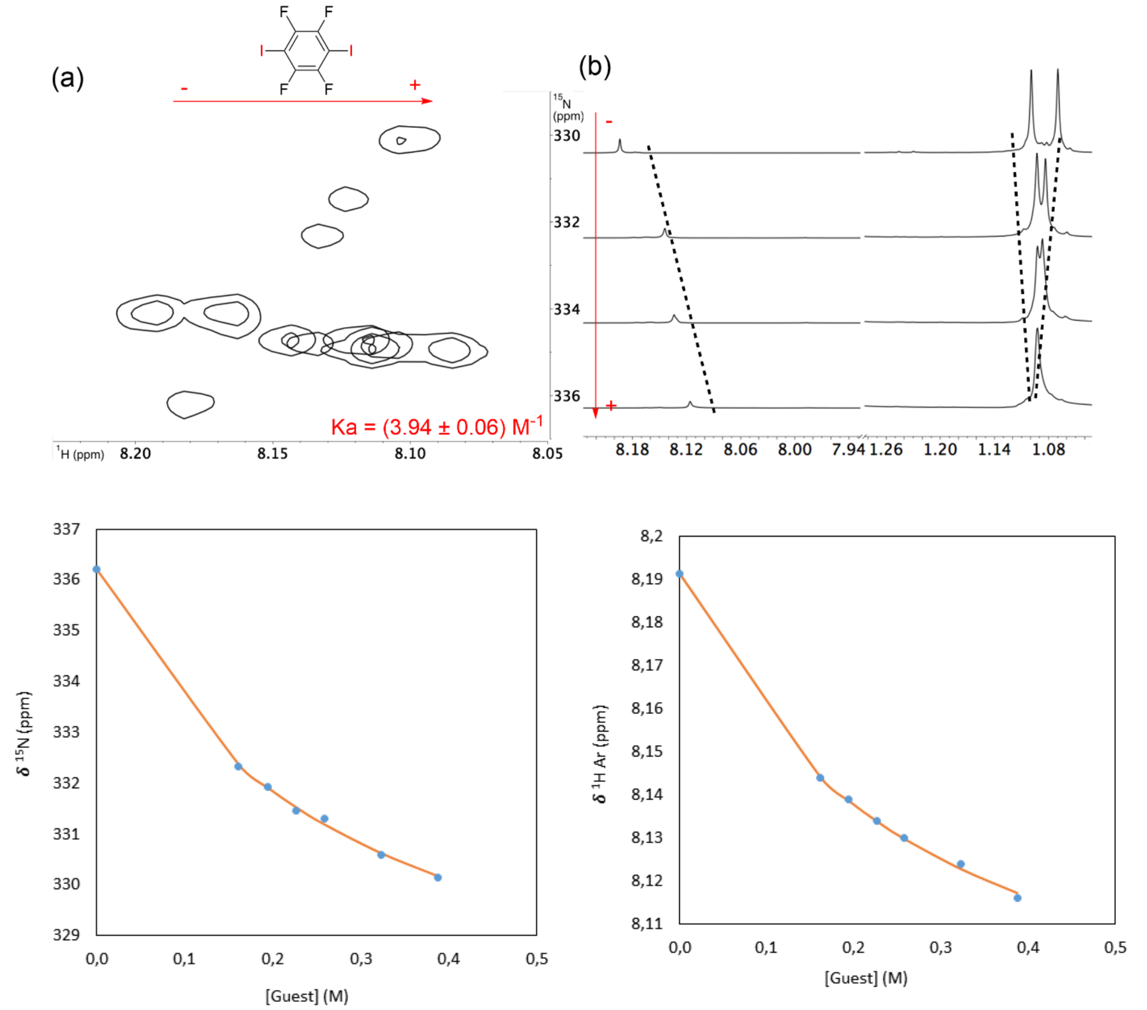

***Figure S29**. Top) NMR titration experiments of (P$_4$)-**2** with **G4**, from 0 to 30 equivalents, and its association constant value. (a) ^{1}H-^{15}N HMBC and (b) ^{1}H NMR. Bottom) Experimental variation of chemical shift (ppm) in the NMR spectrum of (P$_4$)-**2** upon the addition of **G4** (dots) and mathematical fitting to a 1:1 stoichiometric equilibrium (lines).*

6. References

(1) Perrin, D.; Armarego, W. *Purification of Laboratory Chemicals*; Pergamosn Press, 1998.

(2) APEX4 v.2022.1-1 (Bruker AXS Inc., 2022).

(3) SAINT v. 8.40B (Bruker AXS Inc., 2019).

(4) Krause, L.; Herbst-Irmer, R.; Sheldrick, G. M.; Stalke, D. Comparison of Silver and Molybdenum Microfocus X-Ray Sources for Single-Crystal Structure Determination. *J. Appl. Crystallogr.* **2015**, *48* , 3–10.

(5) Sheldrick, G. M. SHELXT - Integrated Space-Group and Crystal-Structure Determination. *Acta Crystallogr. Sect. A Found. Crystallogr.* **2015**, *71* (1), 3–8.

(6) Sheldrick, G. M. Crystal Structure Refinement with SHELXL. *Acta Crystallogr. Sect. C Struct. Chem.* **2015**, *71* , 3–8.

(7) Dolomanov, O. V.; Bourhis, L. J.; Gildea, R. J.; Howard, J. A. K.; Puschmann, H. OLEX2: A Complete Structure Solution, Refinement and Analysis Program. *J. Appl. Crystallogr.* **2009**, *42* , 339–341.

(8) Spek, A. L. Single-Crystal Structure Validation with the Program PLATON. *J. Appl. Crystallogr.* **2003**, *36* , 7–13.

(9) S. M. Budy, M. Khan, X. Chang, S. T. Iacono and D. Y. Son, Semi-fluorinated polyarylenes: Microwave-assisted synthesis and structure–property relationships, *J. Polym. Sci.,* **2020**, 58, 2774-2783.

(10) A. Dhaka, O. Jeannin, I. Jeon, E. Aubert, E. Espinosa and M. Fourmigué, Activating Chalcogen Bonding (ChB) in Alkylseleno/Alkyltelluroacetylenes toward Chalcogen Bonding Directionality Control, *Angew. Chem. Int. Ed.,* **2020**, 59, 23583-23587.

(11) M. J. Frisch, G. W. Trucks, H. B. Schlegel, G. E. Scuseria, M. A. Robb, J. R. Cheeseman, G. Scalmani, V. Barone, B. Mennucci, G. A. Petersson, H. Nakatsuji, M. Caricato, X. Li, H. P. Hratchian, A. F. Izmaylov, J. Bloino, G. Zheng, J. L. Sonnenberg, M. Hada, M. Ehara, K. Toyota, R. Fukuda, J. Hasegawa, M. Ishida, T. Nakajima, Y. Honda, O. Kitao, H. Nakai, T. Vreven, J. A. Montgomery Jr., J. E. Peralta, F. Ogliaro, M. Bearpark, J. J. Heyd, E. Brothers, K. N. Kudin, V. N. Staroverov, R. Kobayashi, J. Normand, K. Raghavachari, A. Rendell, J. C. Burant, S. S. Iyengar, J. Tomasi, M. Cossi, N. Rega, J. M. Millam, M. Klene, J. E. Knox, J. B. Cross, V. Bakken, C. Adamo, J. Jaramillo, R. Gomperts, R. E. Stratmann, O. Yazyev, A. J. Austin, R. Cammi, C. Pomelli, J. W. Ochterski, R. L. Martin, K. Morokuma, V. G. Zakrzewski, G. A. Voth, P. Salvador, J. J. Dannenberg, S. D. Daniels, Ö Farkas, J. B. Foresman, J. V. Ortiz, J. Cioslowski and D. J. Fox, GAUSSIAN 09*,* revision D.01, Gaussian Inc., Wallingford CT, 2016.

(12) Yanai, D. P. Tew and N. C. Handy, A new hybrid exchange–correlation functional using the Coulomb-attenuating method (CAM-B3LYP), *Chem. Phys. Lett.,* **2004**, 393, 51-57.

(13) A. V. Marenich, C. J. Cramer and D. G. Truhlar, Universal Solvation Model Based on Solute Electron Density and on a Continuum Model of the Solvent Defined by the Bulk Dielectric Constant and Atomic Surface Tensions, *J. Phys. Chem. B.,* **2009**, 113, 6378-6396.

(14) B. Mennucci, R. Cammi and J. Tomasi, Excited states and solvatochromic shifts within a nonequilibrium solvation approach: A new formulation of the integral equation formalism method at the self-consistent field, configuration interaction, and multiconfiguration self-consistent field level, *J. Chem. Phys.,***1998**, 109, 2798–2807.

(15) Padula, D.; Lahoz, I. R.; Díaz, C.; Hernández, F. E.; Di Bari, L.; Rizzo, A.; Santoro, F.; Cid, M. M. A Combined Experimental–Computational Investigation to Uncover the Puzzling (Chiro-)Optical Response of Pyridocyclophanes: One- and Two-Photon Spectra. *Chem. Eur. J.* **2015**, *21* (34), 12136–12147.

(16) Álvarez-García, J.; Rubio-Pisabarro, V.; García-Río, L.; Cid, M.M. Deciphering the degree of proton-transfer in pyrido-cyclophanes by chiroptical outcomes in non-aqueous solvents. *Org. Chem. Front.,* **2023**, 10, 5435–5442.

(17) J. Chai, M. Head-Gordon, Long-range corrected hybrid density functionals with damped atom–atom dispersion corrections, *Phys. Chem. Chem. Phys*., **2008**, 10, 6615–6620.

(18) K. A. Peterson; D.Figgen; E. Goll; H. Stoll; M. Dolg*,* Systematically convergent basis sets with relativistic pseudopotentials. II. Small-core pseudopotentials and correlation consistent basis sets for the post-d group 16–18 elements, *J. Chem. Phys.,* **2003***,* 119, 11113–11123.

(19) F. Weigenda, R. Ahlrichs, Balanced basis sets of split valence, triple zeta valence and quadruple zeta valence quality for H to Rn: Design and assessment of accuracy, *Phys. Chem. Chem. Phys.*, **2005**, 7**,** 3297-3305.

(20) B. P. Pritchard, D. Altarawy, B. Didier, T. D. Gibson, T.L. Windus, A New Basis Set Exchange: An Open, Up-to-date Resource for the Molecular Sciences Community, *J. Chem. Inf. Model.* **2019**, *59(11)*, 4814-4820.

(21) R.A. Kendall, T. H. Dunning; Jr., R. J. Harrison, Electron affinities of the first-row atoms revisited. Systematic basis sets and wave functions, *J. Chem. Phys.,* **1992***,* 96, 6796–6806.

(22) K. A. Peterson, B. C. Shepler, D. Figgen, H. Stoll, On the Spectroscopic and Thermochemical Properties of ClO, BrO, IO, and Their Anions, J*. Phys. Chem. A*, **2006**, 110, 51, 13877–13883.

(23) S. Grimme, J. Antony, S. Ehrlich, H. Krieg, A consistent and accurate ab initio parametrization of density functional dispersion correction (DFT-D) for the 94 elements H-Pu, *J. Chem. Phys.* **2010**, 132, 154104.

(24) F. Delano, F. Benner, S. Jang, S. M. Greer, S. Demir, Construction of intermolecular σ-hole interactions in rare earth metallocene complexes using a 2,3,4,5-tetraiodopyrrolyl anion, *Chem. Sci*., **2024**, 15, 13389-13404.

(25) M. Beau, S. Lee, S. Kim, W. S. Han, O. Jeannin, M. Fourmigué, E. Aubert, E. Espinosa, I.R. Jeon, Strong σ-Hole Activation on Icosahedral Carborane Derivatives for a Directional Halide Recognition, *Angew. Chem. Int. Ed*. **2021**, 60, 366 – 370.

(26) M. Breugst, J. J. Koenig, σ-Hole Interactions in Catalysis, *Eur. J. Org. Chem.,* **2020**, 5473–5487.

(27) A. Dhaka, O. Jeannin, I.R. Jeon, E. Aubert, E. Espinosa, M. Fourmigué, Activating Chalcogen Bonding (ChB) in Alkylseleno/Alkyltelluroacetylenes toward Chalcogen Bonding Directionality Control, *Angew. Chem. Int. Ed*. **2020**, 59, 23583 –23587.

(28) T. Lu, F. Chen, Multiwfn: A Multifunctional Wavefunction Analyzer, *J. Comput. Chem*., **2012**, 33, 580-592.

(29) T. Lu, A comprehensive electron wavefunction analysis toolbox for chemists, Multiwfn, *J. Chem. Phys.*, **2024**, 161, 082503.

(30) BindFit v0.5, http://supramolecular.org/
