# Supplementary figures and images for: Shape-Matching and Halogen Bonding in Chiral Pyrazine-Allene Hosts: Confining an Unstable Guest Conformation
Source: Org Lett. 2025 Jun 24;27(26):7164–9. doi: 10.1021/acs.orglett.5c02075 (PMC12235685; doi:10.1021/acs.orglett.5c02075)

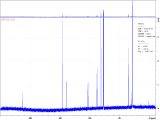

Supplement: Supplementary file 2 [file ol5c02075_si_002.zip › (P4)-4/13C/pdata/1/thumb.png]

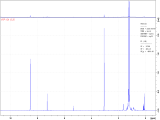

Supplement: Supplementary file 2 [file ol5c02075_si_002.zip › (P4)-4/1H/pdata/1/thumb.png]

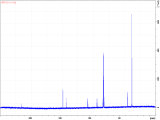

Supplement: Supplementary file 2 [file ol5c02075_si_002.zip › (P2)-2/13C/pdata/1/thumb.png]

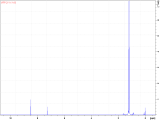

Supplement: Supplementary file 2 [file ol5c02075_si_002.zip › (P2)-2/1H/pdata/1/thumb.png]

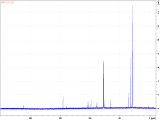

Supplement: Supplementary file 2 [file ol5c02075_si_002.zip › (P2)-3/13C/pdata/1/thumb.png]

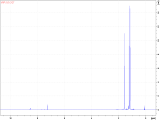

Supplement: Supplementary file 2 [file ol5c02075_si_002.zip › (P2)-3/1H/pdata/1/thumb.png]
